# Natural products targeting mitochondrial homeostasis: a four-dimensional framework integrating dynamics, mitophagy, pharmacokinetics, and translational safety

**DOI:** 10.3389/fphar.2026.1911495

**Published:** 2026-09-18

**Authors:** Zhi-Yun Xiang, Xiu-Hua Yang, Wen-Ying Lan, Xin-Yu Xiao, Qun Huang, Lin-Lin Yang

**Affiliations:** 1 Chengdu Xinjin District Hospital of Traditional Chinese Medicine, Chengdu, China; 2 Department of General Surgery, Deyang Sixth People’s Hospital, Deyang, China; 3 Chengdu University of Traditional Chinese Medicine, Chengdu, China; 4 Hospital of Chengdu University of Traditional Chinese Medicine, Chengdu, China; 5 Affiliated Meishan Hospital of Chengdu University of Traditional Chinese Medicine / Traditional Chinese Medicine Hospital of Meishan, Meishan, Sichuan Province, China

**Keywords:** gut microbiota–mitochondria axis, mitochondrial dynamics, mitohormesis, mitophagy, natural products, pharmacokinetics

## Abstract

**Background:**

Dysregulated mitochondrial dynamics, including fission, fusion, biogenesis, and mitophagy, underlie the pathogenesis of many diseases. Natural products have the potential for multi-target therapy, but insufficient understanding of their mechanisms of action and pharmacokinetic limitations hinder their clinical translation.

**Objective:**

This review proposes a regulatory framework covering the four dimensions of biogenesis, morphology, quality control, and defense, aiming to systematically clarify how natural products regulate mitochondrial function and critically evaluate their translational potential. The framework emphasizes that inter-organelle contact platforms, such as mitochondria–endoplasmic reticulum contact sites (MERCs) and mitochondrial–lysosomal contact sites (MLCs), are key sites through which natural products exert regulatory effects.

**Methods:**

Major scientific databases, including PubMed, Web of Science, and Scopus, were searched, and evidence on mechanisms, pharmacokinetics, and toxicology from preclinical and clinical studies was comprehensively reviewed.

**Results:**

Natural products can selectively regulate key mitochondrial pathways and regulatory nodes, including the AMPK/SIRT1/PGC-1α axis involved in mitochondrial biogenesis, the DRP1 axis involved in mitochondrial fission, and the PINK1/Parkin pathway involved in mitophagy. Representative metabolites include resveratrol, curcumin, and *Coptis chinensis*-derived alkaloids. A key finding of this review is that the intestinal microbiota plays an important role in converting low-bioavailability precursors, such as tannins, into systemically active metabolites, such as urolithin A (UA), thus inducing mitophagy. Although these metabolites usually show strong activity *in vitro*, their low oral bioavailability and rapid metabolism lead to significant pharmacokinetic/pharmacodynamic (PK/PD) mismatch, which remains a major obstacle to their clinical translation.

**Conclusion:**

Natural products are powerful multidimensional regulators of mitochondrial homeostasis. In order to fully realize their therapeutic potential, strategies must be adopted to overcome PK/PD mismatch, including the development of targeted delivery systems, in-depth analysis of microbiome–metabolite interactions, and the integration of artificial intelligence and CRISPR-based technological innovation to support precision therapy.

## 1 Introduction

Mitochondria form a dynamic and interconnected network. Their morphology and function are controlled by strictly regulated processes, which are collectively called mitochondrial dynamics. These processes enable mitochondria to adapt to fluctuations in energy demand and environmental stress (Chen W. et al., 2023). Against this background, natural products with diverse structures and multiple biological activities have become potential candidate regulators for the restoration of mitochondrial function in disease states (Guo et al., 2023; Rahmani et al., 2023).

The global significance of mitochondrial homeostasis in disease pathogenesis is underscored by the escalating burden of conditions rooted in mitochondrial dysfunction. Mitochondrial disorders collectively affect approximately one in 5,000 individuals worldwide, with pathogenic variants in mitochondrial DNA (mtDNA) or nuclear-encoded mitochondrial genes implicated in more than 250 distinct diseases (Wen et al., 2025). Beyond primary mitochondrial diseases, secondary mitochondrial dysfunction serves as a convergent pathogenic mechanism across major non-communicable diseases that dominate the global health landscape. Cardiovascular diseases, the leading cause of mortality worldwide, accounting for 17.9 million deaths annually, involve profound mitochondrial bioenergetic failure and aberrant fission–fusion dynamics during ischemia–reperfusion injury and chronic heart failure (Javadov et al., 2018; Tong et al., 2020). Neurodegenerative diseases, including Alzheimer and Parkinson diseases, which afflict more than 50 million people globally and constitute a mounting socioeconomic challenge amid aging populations, are characterized by early and progressive disruption of mitochondrial quality control networks (Nakamura et al., 2021; Wang H. et al., 2024). Metabolic syndromes, notably type 2 diabetes and non-alcoholic fatty liver disease (NAFLD), affecting more than 460 million and 25% of the global adult population, respectively, exhibit intrinsic links between insulin resistance and impaired mitochondrial biogenesis and dynamics (Gawlowski et al., 2012; John et al., 2022). Furthermore, the Warburg effect and chemoresistance in cancer, which claimed nearly 10 million lives in 2020, exploit remodeled mitochondrial dynamics to sustain proliferative advantage and evade therapeutic pressure (Chen W. et al., 2023; Li F. et al., 2023). This pervasive involvement of mitochondrial dysfunction across diverse disease categories, not merely as a downstream consequence but often as an initiating or amplifying pathogenic driver, highlights an urgent unmet medical need for targeted interventions that can restore mitochondrial homeostasis at the network level. Natural products, by virtue of their evolutionarily optimized polypharmacological profiles, represent promising candidates for addressing this pathophysiological complexity.

However, the clinical translation of natural products faces a fundamental paradox that pervades the entire research field and critically constrains the physiological credibility of all mechanistic conclusions. A wide range of natural products exhibit potent bioactivity under *in vitro* culture conditions at concentrations between 5 µm and 50 µM. Yet human systemic drug exposure after oral administration often falls one or more orders of magnitude below such thresholds. The root causes include extremely low oral bioavailability, rapid first-pass metabolism, and extensive phase II conjugation reactions. We term this universal contradiction the Core Paradox, which is not a minor isolated pharmacokinetic defect but a systemic challenge that undermines the translational value of all *in vitro* experimental outcomes. Multiple representative compounds illustrate this paradox clearly. Curcumin suppresses DRP1 activity and activates the SIRT3/OPA1 pathway at concentrations from 5 µM to 50 µM in hepatic and neuronal cell models; even with an 8 g oral dose, its maximum plasma concentration in humans only reaches approximately 1.7 µM. EGCG requires concentrations higher than 10 µM to trigger SIRT1-mediated PGC-1α deacetylation, yet its plasma concentration in humans only registers at the low-nanomolar level after oral intake. Resveratrol exerts functional effects at 10–50 µM in podocyte and neuronal models; its oral bioavailability stands at roughly 6%, and conjugated metabolites dominate circulating levels while free aglycone concentrations remain negligible. Berberine activates AMP-activated protein kinase (AMPK) across multiple cell types at 1–25 μM, but its oral bioavailability only ranges from 0.37% to 1%. Quercetin generates therapeutic effects at 5–20 µM under inflammatory conditions; the maximum plasma concentration of its aglycone form in humans is only approximately 7.6 µM, and its penetration across the blood–brain barrier remains limited. This widespread imbalance between pharmacokinetic and pharmacodynamic indicators raises a critical research question: if human systemic drug levels cannot meet the effective concentrations observed in cell experiments, can the mechanisms summarized from *in vitro* work reflect real physiological processes *in vivo*, or do these findings merely derive from artificial culture environments that mislead translational expectations?

Three mutually supportive theoretical perspectives proposed in this review can partially resolve the Core Paradox. The first perspective centers on mitohormesis, introduced in Section 4.5.2. This theory states that low concentrations reachable in human bodies can initiate adaptive cellular defense responses rather than directly binding to target proteins, providing a reasonable mechanistic explanation for natural product efficacy even when systemic exposure stays below *in vitro* thresholds. The second perspective focuses on the gut microbiota–mitochondria axis, elaborated in Section 4.6.4. Intestinal microbes can convert poorly absorbable precursor substances such as ellagitannins into urolithin A and other metabolites with superior pharmacokinetic and pharmacodynamic profiles. The third perspective highlights inter-organelle contact platforms, including mitochondria–endoplasmic reticulum contact sites (MERCs) and mitochondrial–lysosomal contact sites (MLCs). These structural microdomains can accumulate natural products to localized concentrations sufficient to trigger coordinated intracellular signaling cascades. The three analytical frameworks are not independent of each other; they serve as consistent interpretive tools running through all subsequent chapters to explain how natural products can deliver therapeutic benefits despite unfavorable systemic pharmacokinetic properties. As a uniform analytical rule for this manuscript, every discussion of specific natural products in later mechanistic sections will compare their documented *in vitro* effective concentrations against measurable human systemic exposure data and cite this section’s Core Paradox for sustained critical evaluation of translational prospects.

Apart from the Core Paradox-related challenge, existing research suffers another major limitation. Most previous publications investigate only single chemical classes of natural products or isolated mitochondrial functional processes, which prevents comprehensive systematic analysis of their regulatory networks (Guo et al., 2023). As mechanistic research deepens, scholars increasingly recognize that most natural products do not simply activate or inhibit a single mitochondrial pathway; instead, they remodel the entire mitochondrial functional network through coordinated multi-target regulation (Chen Q. R. et al., 2023). To address this research gap, this review constructs a four-dimensional regulatory framework covering mitochondrial biogenesis, morphological homeostasis, quality control, and antioxidant defense. The framework breaks the analytical constraints of single-pathway research and captures integrated characteristics of the full mitochondrial life cycle.

From this integrated analytical viewpoint, the current review establishes a unified conceptual system that links molecular mechanisms, *in vivo* physiological relevance, and clinical translational potential. The overall chapter arrangement follows this logical sequence: We first outline the molecular basis of mitochondrial dynamics (Section 3). Then, we analyze how natural products reshape mitochondrial function within our four-dimensional framework (Section 4) and critically evaluate the pharmacokinetic and safety barriers that constitute the Core Paradox of natural products therapy (Section 5). Finally, we discuss technology-driven strategies to overcome these challenges (Section 6).

## 2 Materials and methods

### 2.1 Search strategy

The Preferred Reporting Items for Systematic Reviews and Meta-Analyses (PRISMA) 2020 statement was fully adopted to guide the implementation and standardized reporting of this systematic review. We retrieved literature from four authoritative academic databases: PubMed, Web of Science, Scopus, and the China National Knowledge Infrastructure (CNKI), with the retrieval time frame spanning from the year 2000 to January 2026. Standardized subject headings combined with free-text keywords were adopted for literature searching. Core retrieval terms, including “natural products”, “mitochondrial homeostasis”, “mitochondrial dynamics”, “mitochondrial biogenesis”, “mitophagy, oxidative defense”, and “gut microbiota–mitochondria axis”, were paired with the linking operator “AND” for combined retrieval to capture all relevant preclinical research records on natural products regulating mitochondrial function. After electronic database retrieval, we additionally manually reviewed the reference lists of all retrieved full-text articles to supplement potentially overlooked relevant studies.

### 2.2 Eligibility criteria and study selection

Eligible literature consisted of peer-reviewed original articles written in either English or Chinese that were published between 2000 and 2026. Preclinical *in vitro* cellular or *in vivo* animal studies should center on the modulatory impacts of natural products covering four core mitochondrial homeostatic modules encompassing mitochondrial biogenesis, fission–fusion equilibrium, mitophagy, and oxidative defense. Qualified studies shall implement single interventions with natural product monomers or crude extracts such as polyphenols, flavonoids, alkaloids, and ginsenosides, while providing fully explicit experimental information involving the chemical identity of target natural products, intervention concentration or dosage, cell/animal model classification, and administration routes. All included works were required to establish blank controls, solvent vehicle controls, or untreated mitochondrial dysfunction model groups and report at least one core biomarker linked to mitochondrial homeostasis such as PGC-1α, DRP1, MFN1/2, PINK1/Parkin, reactive oxygen species (ROS), MERCs and MLCs, or complete pharmacokinetic and toxicological data of tested natural compounds.

Studies were ruled out if they were non-peer-reviewed resources such as conference abstracts, preprints, and grey literature, and articles with no direct relevance to pure computational simulation research or compound combination intervention experiments unable to isolate the independent efficacy of individual natural products were also excluded. Duplicate publications and works carrying redundant experimental datasets were not eligible for inclusion. Studies without definite experimental parameters, including unstated natural product dosages, undefined cell or animal models, and undocumented administration regimens, as well as manuscripts that merely detect peripheral non-mitochondrial indicators without any mitochondrial-associated detection readouts, were also excluded.

### 2.3 Data extraction

A unified standardized data extraction table was pre-designed and tested prior to formal data collection to guarantee complete, consistent, and accurate information sorting from all eligible studies. Extracted information covered basic publication information, tested natural product categories and chemical structures, cell/animal model construction schemes, specific intervention protocols including compound concentration, dosage, and administration route, setting forms of control groups, core detection indicators corresponding to the four-dimensional mitochondrial regulatory framework, related signaling pathway results, and recorded pharmacokinetic and toxicological experimental outcomes. For data only displayed in image graphs without numerical values, professional image quantification tools were used to extract original data. When key experimental data were missing in original articles, corresponding authors were contacted to obtain supplementary raw data whenever possible.

### 2.4 Study screening procedure

Two independent researchers completed the two-round literature screening process separately. The first round screened titles and abstracts to exclude obviously irrelevant records; the second round carried out full-text reading of remaining articles to judge whether they fully meet the preset inclusion standards. Any inconsistent screening judgments generated by the two researchers were resolved through in-depth discussion; if consensus could not be reached, a third senior researcher participated in evaluation to finalize the screening result.

### 2.5 Data analysis

This review adopted systematic qualitative narrative analysis to comprehensively organize and interpret existing preclinical evidence. We systematically sorted and summarized the molecular regulatory mechanisms of various natural products targeting mitochondrial biogenesis, morphological balance, mitophagy, and oxidative defense, focused on analyzing the core pharmacokinetic paradox between *in vitro* effective concentration and *in vivo* systemic exposure, and collated the toxicity characteristics and corresponding mitigation strategies of different types of natural products. The gut microbiota–mitochondria axis, the mitohormesis effect, and inter-organelle contact platforms (MERCs and MLCs) were integrated to interpret the internal logical connection of multidimensional mitochondrial regulation networks. The translational bottlenecks and innovative therapeutic strategies for natural product mitochondrial regulators were summarized.

### 2.6 Screening flow overview

A total of 10,916 original records were initially retrieved from all target databases. After automatic and manual removal of duplicate documents, 5,093 unique citations remained. Preliminary screening based on titles and abstracts excluded 3,823 irrelevant records, leaving 1,270 full-text articles for detailed eligibility assessment. Upon full-text evaluation against predefined criteria, 210 qualified original preclinical studies were ultimately included for comprehensive systematic analysis. The complete PRISMA screening flowchart is shown in Figure 1. All included literature adopted English or Chinese peer-reviewed original research forms.

**FIGURE 1:**
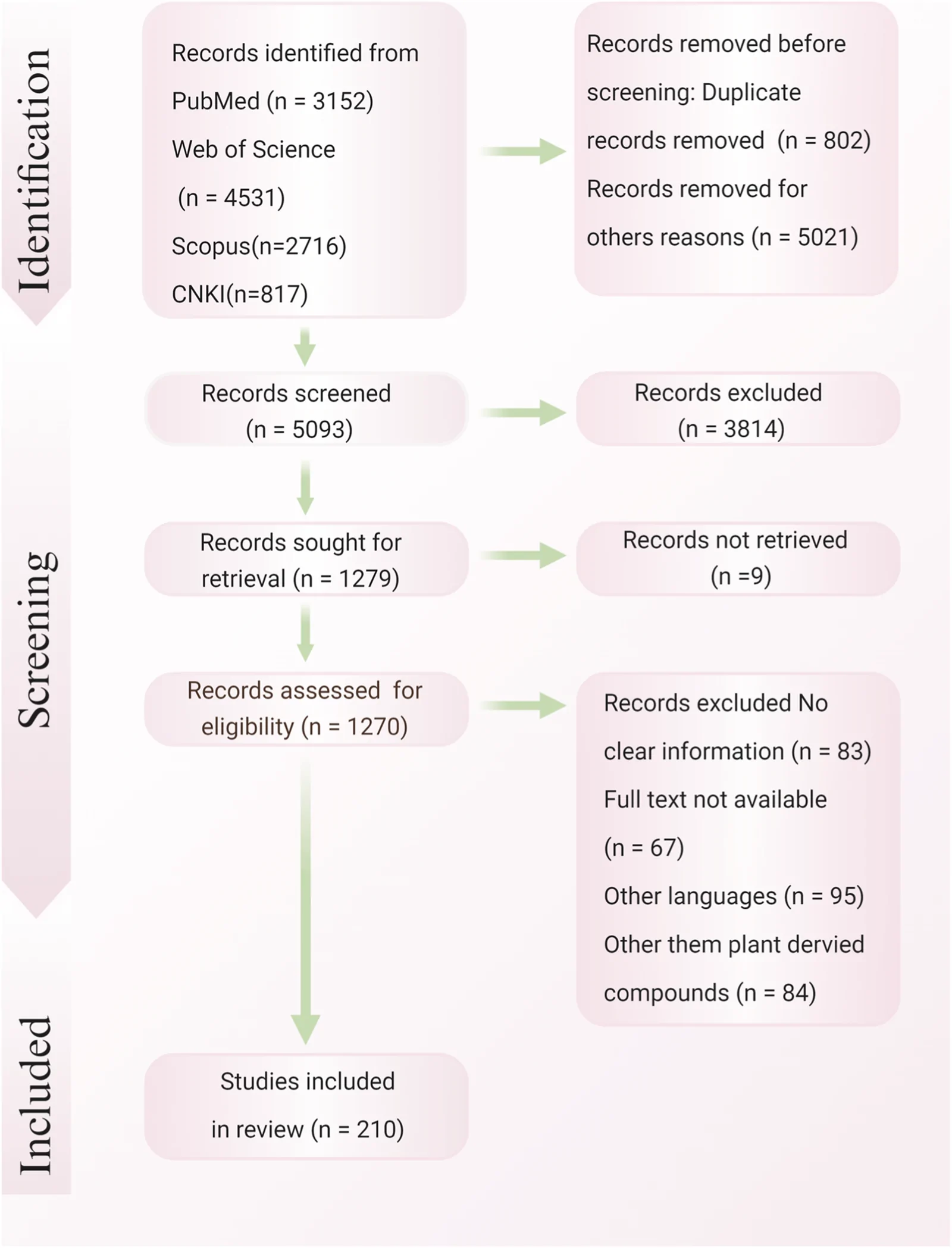
PRISMA flowchart for study selection process.

## 3 Molecular foundations of mitochondrial dynamics and their dysregulation in disease

The homeostatic coordination of mitochondrial dynamics has drawn increasing interest as a target for emerging precision therapeutic strategies. These dynamic processes represent actionable therapeutic targets amenable to pharmacological inhibition (e.g., DRP1 inhibitors such as Mdivi-1) (Marx et al., 2024), precision genome editing (e.g., MFN2-guided tools) (Adhikary et al., 2023), and AI-assisted quantitative morphological analysis via single-cell live imaging (Johnson et al., 2020) (Figure 2). The relevance of these methods in neurodegenerative diseases, metabolic syndromes, and ischemic injury is becoming increasingly important (Johnson et al., 2020). Despite significant progress, the field still faces the formidable challenge of decoding this highly complex regulatory network and translating this knowledge into effective, safe, and clinically feasible interventions. The main challenges include clarifying the specific role of mitochondrial dynamics in health and disease; determining the therapeutic potential of natural products as regulators of these pathways; analyzing regulatory layers involving non-coding RNA and inter-organelle communication, including MERCs and MLCs; and overcoming bottlenecks in delivery and safety. In short, these efforts will clarify the core role of mitochondrial dynamics in physiological processes and lay the foundation for mechanism-based therapeutic interventions.

**FIGURE 2:**
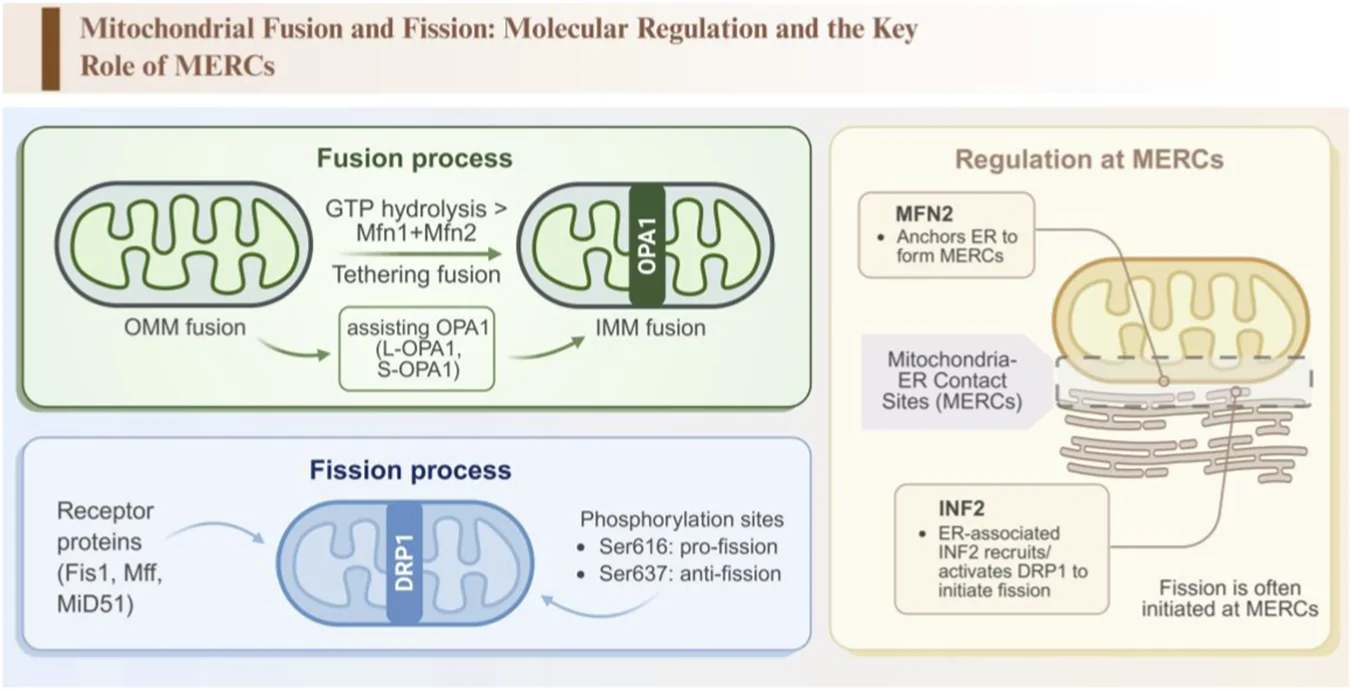
Orchestrating mitochondrial dynamics: The molecular regulation of fusion and fission and the central role of MERCs. The diagram shows the molecular mechanisms of mitochondrial fusion and fission, focusing on the regulation at MERCs. The fusion process includes outer mitochondrial membrane (OMM) fusion driven by GTP hydrolysis and mediated by mitofusins MFN1 and MFN2, followed by inner mitochondrial membrane (IMM) fusion mediated by OPA1 isoforms L-OPA1 and S-OPA1. The mitochondrial fission process is initiated by receptor proteins (FIS1, MFF, and MiD51) and involves a DRP1 contractile ring-like structure. The activity of DRP1 is regulated by phosphorylation at specific sites (Ser616 promotes fission, while Ser637 inhibits fission). At MERCs, MFN2 anchors the endoplasmic reticulum to the mitochondria to form contact sites, and the ER-associated INF2 recruits and activates DRP1 at these sites, thus initiating fission.

### 3.1 The fission–fusion mechanism: regulators of mitochondrial form and function

#### 3.1.1 Mitochondrial fusion regulation

Mitochondrial fusion refers to the coordinated fusion of the mitochondrial outer membrane (OMM) and the inner mitochondrial membrane (IMM) to form a larger and more complete organelle (Javadov et al., 2018). OMM fusion is mainly mediated by mitochondrial fusion proteins mitofusin 1 (MFN1) and mitofusin 2 (MFN2) (Chan, 2012). Deletion or downregulation of either protein will impair fusion, leading to mitochondrial dysfunction and severe cellular defects. MFN2 also serves as a key tethering protein for MERCs, establishing an intrinsic mechanistic link between mitochondrial fusion and organelle contact sites (Chen et al., 2003; Wang H. et al., 2024). Therefore, natural products that can upregulate MFN2, such as resveratrol and paeonol, may simultaneously enhance mitochondrial fusion and stabilize endoplasmic reticulum–mitochondria communication. IMM fusion is regulated by another key GTPase, optic atrophy 1 (OPA1). OPA1 exists as membrane-anchored long forms (L-OPA1) and soluble short forms (S-OPA1). L-OPA1 faces the intermembrane space. The balance between L-OPA1 and S-OPA1 is regulated by the stress-activated protease OMA1 and the constitutively active protease YME1L, which determine the efficiency of mitochondrial fusion. Although OPA1-defective cells can complete OMM fusion, IMM fusion is blocked, resulting in mitochondrial fragmentation and fusion defects (Javadov et al., 2018; Wang H. et al., 2024).

#### 3.1.2 Mitochondrial fission regulation

In contrast, mitochondrial fission divides a single mitochondrion into two daughter mitochondria through the coordinated contraction of the IMM and the OMM. This process is crucial for the distribution of organelles, the regulation of organelle copy numbers, and the selective removal of damaged mitochondria. The initiation of fission depends on the physical remodeling of the MERCs. High-resolution imaging shows that the endoplasmic reticulum (ER) actively wraps around mitochondria and applies mechanical force at a specific site to form a deep constriction with an average diameter of approximately 300 nm; this process occurs before DRP1 is recruited. This mechanical constriction is driven by ER-associated inverted formin 2 (INF2)-mediated actin polymerization, which forms actin filaments at the endoplasmic reticulum–mitochondrial interface. INF2 also helps to establish stable MERCs (Rowland and Voeltz, 2012; Chakrabarti et al., 2018). Therefore, MERCs are a key platform for coordinating mitochondrial fission activation. These sites promote the local transfer of Ca^2+^ from the ER to mitochondria, further stimulating fission machinery. The mitochondrial outer membrane E3 ligase MAPL catalyzes the SUMOylation of DRP1 (Ma et al., 2020), which further stabilizes these contact sites and prolongs the exchange of calcium and lipids (Prudent and McBride, 2017).

The fission process is mainly regulated by the cytosolic GTPase DRP1. After activation, DRP1 is recruited to the OMM through receptor proteins including FIS1, MFF, MiD49, and MiD51 (Chan, 2012; Logan, 2003). In these MERCs, key proteins such as INF2 and actin nucleator Spire1C produce actin filaments and promote DRP1 assembly. DRP1 forms a ring-like or spiral structure around the mitochondria; then GTP hydrolysis drives membrane constriction and scission. Although these receptors can independently recruit DRP1, there is evidence that MiD49/51 and MFF work together in the DRP1 complex to regulate fission kinetics (Zerihun et al., 2023). This spatial coordination ensures the precise fission of mitochondria under metabolic stress or calcium signaling (Rowland and Voeltz, 2012).

DRP1 activity is strictly regulated by post-translational modifications (PTMs), with phosphorylation as the main regulatory mechanism. The phosphorylation of Ser616 (usually driven by kinases such as CDK1/5 or ERK1/2) usually promotes fission (Chang et al., 2021; Ma et al., 2020), while phosphorylation at Ser637 (usually mediated by PKA or AMPK) usually inhibits the activity of DRP1, thus achieving bidirectional regulation (Zerihun et al., 2023; Gawlowski et al., 2012; Xia et al., 2024; Adhikary et al., 2023). Natural products usually target these upstream kinases (e.g., sanggenon C inhibits ERK1/2), thus indirectly regulating DRP1 activity.

In addition to phosphorylation, DRP1 is also regulated by a variety of modifications, such as S-nitrosylation (Nakamura et al., 2021), O-GlcNAcylation (Gawlowski et al., 2012), and ubiquitination. These regulatory layers may converge to form a refined PTM code, which dynamically regulates DRP1-mediated fission and provides multiple entry points for natural products intervention.

### 3.2 Mitophagy: the selective clearance of damaged mitochondria

Mitophagy is a specialized form of autophagy that maintains mitochondrial quality by identifying, isolating, and degrading dysfunctional, severely damaged, or excessive mitochondria, thus maintaining the integrity of the mitochondrial network. This process is closely related to mitochondrial dynamics, including fusion and fission, and is central to the intracellular mitochondrial quality control (MQC) system. Fusion maintains the integrity and functional complementarity of the network, while fission separates damaged fragments from the network to achieve selective removal. When local damage occurs, such as a sharp drop in mitochondrial membrane potential (ΔΨm) or excessive accumulation of reactive oxygen species (ROS), ER wrapping and INF2-mediated actin polymerization initiate fission at MERCs and form a constriction site. Subsequently, DRP1-mediated fission severs and separates the damaged mitochondrial region or the entire organelle so that it can be recognized and removed by the autophagic machinery (Rowland and Voeltz, 2012; Chakrabarti et al., 2018; Ul Fatima and Ananthanarayanan, 2023; Onishi et al., 2021). Therefore, the appropriate mitochondrial fission mechanism is a prerequisite for efficient and accurate mitophagy (Ryu et al., 2024; Chan, 2012). In addition, recent studies have shown that MLCs can be used as platforms to initiate mitophagy after fission. Lysosomes are connected to mitochondria through MLCs to promote efficient lipid and signal exchange, thus coordinating the final stage of degradation (Wong et al., 2018) (Figure 3).

**FIGURE 3:**
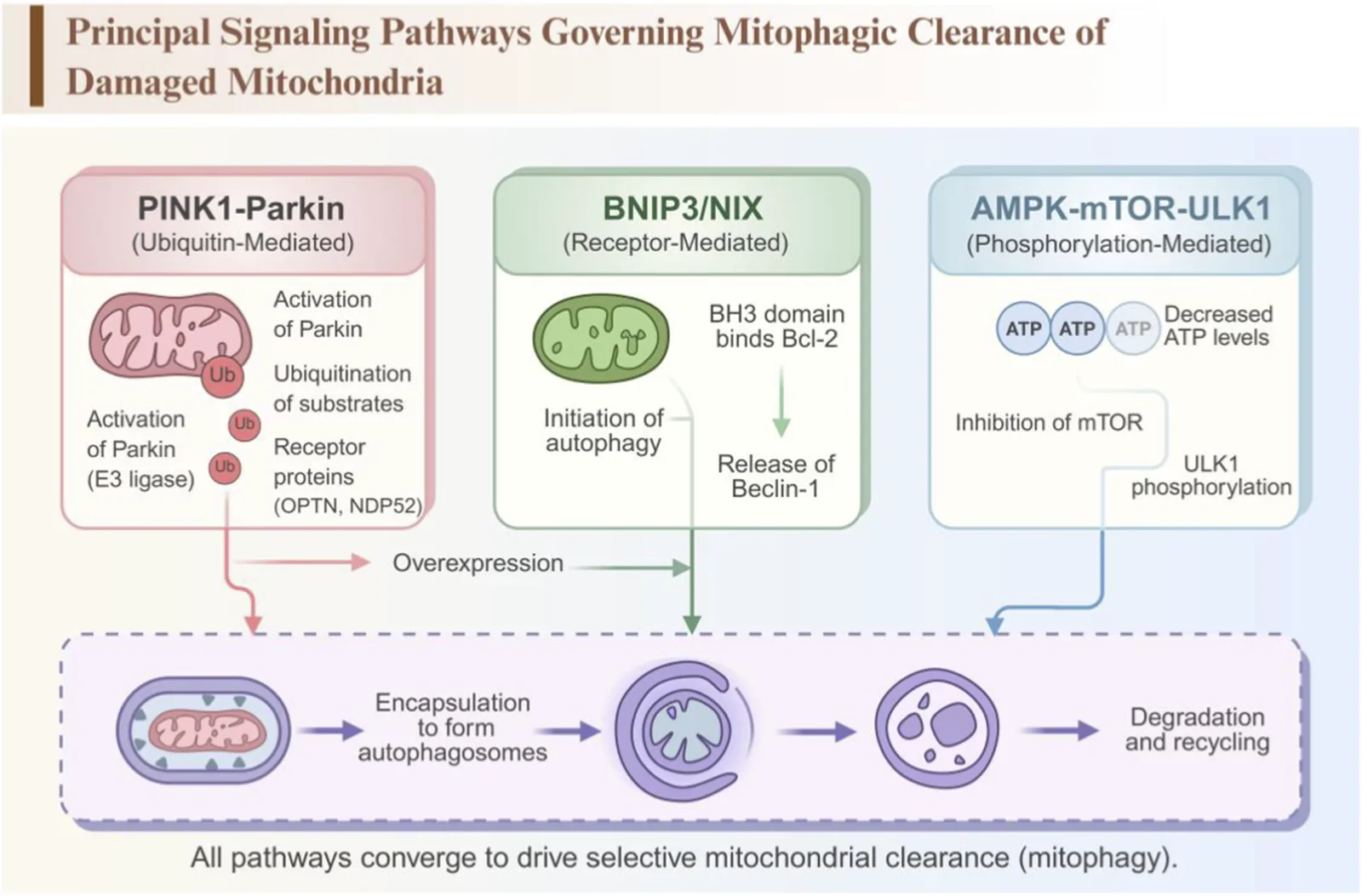
Mitochondrial quality control: mapping the core signaling pathways for the mitophagic clearance of damaged organelles. The schematic diagram shows the three core mitophagy pathways. The PINK1/Parkin pathway (ubiquitin-mediated, left) involves the activation of Parkin (E3 ligase) and substrate ubiquitination, thereby recruiting receptor proteins (OPTN, NDP52). The BNIP3/NIX pathway (receptor-mediated, center) involves binding of its BH3 domain to Bcl-2, thereby releasing Beclin-1 and initiating autophagy. The AMPK/mTOR/ULK1 pathway (phosphorylation-mediated, right) is triggered by ATP depletion, resulting in mTOR inhibition and ULK1 activation. All pathways eventually converge to drive selective mitochondrial clearance (mitophagy), including sequestration of mitochondria within autophagosomes, followed by degradation and recycling.

In terms of mechanism, mitophagy is a coordinated cascade reaction. Among the relevant pathways, the most extensively studied is the pathway triggered by the PINK1/Parkin axis (Chan, 2012; Nunnari and Suomalainen, 2012). In healthy mitochondria, when the mitochondrial membrane potential (ΔΨm) remains stable, PINK1 is rapidly imported across the OMM and IMM and cleaved by mitochondrial proteases. However, after depolarization, the import of PINK1 is blocked, resulting in its accumulation and stable presence on the OMM. Stabilized PINK1 then recruits and activates the E3 ubiquitin ligase Parkin, thus driving the polyubiquitination of OMM substrates. These ubiquitin chains act as mitophagy signals. Subsequently, the phagophore engulfs the target organelle, forms autophagosomes, and fuses with lysosomes for degradation (Wang H. et al., 2024; Onishi et al., 2021; Pickles et al., 2018). In addition to the PINK1/Parkin pathway, other pathways, including BNIP3/NIX-mediated receptor-dependent mitophagy, also regulate mitochondrial recognition for clearance (Qi et al., 2025). Upstream, the AMPK/mTORC1/ULK1 axis acts as a central regulatory hub for autophagy activation under energy stress: activation of AMPK during ATP depletion promotes autophagy through direct phosphorylation of ULK1, while relieving mTOR-mediated inhibition (Yang et al., 2024). Natural products such as berberine usually use this pathway to induce mitophagy. Selective autophagy receptors, such as OPTN and NDP52, can identify ubiquitinated outer mitochondrial membrane proteins and connect damaged mitochondria to LC3 through their LC3-interacting region (LIR) to ensure targeted engulfment (Nunnari and Suomalainen, 2012; Qi et al., 2025). Note that lysosomes are linked to mitochondria through MLCs.

### 3.3 Dysregulated mitochondrial dynamics in disease pathogenesis

Once the delicate dynamic balance of mitochondria is disrupted, energy deficiency, oxidative stress, and apoptosis ensue, thereby making this imbalance a core pathogenic driver of many diseases. The multi-target pharmacological properties of natural products are particularly well suited to addressing this complexity (Chen W. et al., 2023). The molecular characteristics of dynamic imbalance will vary according to disease context, which provides an opportunity for precise intervention. However, a major limitation of current natural products research is that it often uses general disease models (such as Aβ-induced neurotoxicity) while ignoring stage-specific dynamic defects. For example, patients with early and advanced Alzheimer disease (AD) may show different DRP1 phosphorylation patterns, but natural products are rarely evaluated across disease progression, which limits their translational value. This highlights the need to develop a model that can more accurately reflect the temporal progression of human disease.

In neurodegenerative diseases (NDs), dynamic defects usually present disease-specific forms. In AD, abnormal accumulation of β-amyloid (Aβ) is a characteristic feature (Nakamura et al., 2021). Aβ oligomers abnormally activate DRP1 through kinases such as CDK5 and CaMKII, increasing Ser616 phosphorylation and exacerbating fission and network fragmentation. ATP generation collapses, oxidative damage intensifies (Tapias, 2019), and neuronal apoptosis is ultimately promoted (Cai and Tammineni, 2016). In Parkinson disease, impairment of the PINK1/Parkin pathway compromises mitophagy (Wang H. et al., 2024; Pickles et al., 2018). After the PINK1/Parkin pathway is activated, the damaged mitochondria are anchored on MERCs by BAX-BAK oligomers, and the LC3-II phagophore/autophagic structures are formed at these sites. The process relies on the PI3P microdomains formed at MERCs; the absence of MFN2 disrupts the positioning of PI3P (Hsieh et al., 2019). Salvianolic acid A is a phenolic acid derived from *Salvia miltiorrhiza*. It promotes the generation of PI3P by enhancing the activity of VPS34 kinase and accelerating autophagosome assembly at MERCs (Du et al., 2021). Persistent dysfunctional mitochondria enhance reactive oxygen species production and release pro-apoptotic factors (Dorn and Kitsis, 2015; Chan, 2012). Activated DRP1 further promotes pathological α-synuclein aggregation, forming a vicious cycle of dopaminergic neuron loss. In Huntington disease (HD), mutant huntingtin (mHTT) directly inhibits MFN2, leading to severe network fragmentation and abnormal mitochondrial calcium handling, eventually resulting in neuronal apoptosis (Seegren et al., 2023; Li R. et al., 2023). MERC disruption is also considered to be associated with calcium-handling abnormalities.

In cardiovascular diseases (CVDs), dynamic homeostasis is crucial to meet the high energy demands of cardiomyocytes. During myocardial ischemia–reperfusion (I/R) injury, acute DRP1-dependent fission surges occur during reperfusion, leading to mitochondrial fragmentation that predisposes to cytochrome c release and activation of the apoptotic cascade (Javadov et al., 2018). At the same time, ROS bursts and calcium overload damage MFN2 and OPA1, leading to collapse of the mitochondrial membrane potential and expansion of the infarct area. Conversely, maintaining or enhancing MFN2- and OPA1-mediated fusion can reduce reperfusion injury (Tong et al., 2020). A prominent feature of chronic heart failure (CHF) is the downregulation of MFN2 expression, which leads to network fragmentation, impaired energy coupling, and persistent energy deficiency, thus weakening cardiac compensatory reserve.

In metabolic disorders, mitochondrial abnormalities directly lead to insulin resistance in type 2 diabetes (T2D) (Gawlowski et al., 2012). Defects in mitochondrial dynamics impair insulin-stimulated GLUT4 translocation, and disruption of mitochondria-ER contacts will further aggravate insulin resistance (Reddy, 2017). In non-alcoholic fatty liver disease (NAFLD), excessive DRP1 activation exacerbates lipotoxicity and promotes the progression of lipid toxicity and fibrosis of hepatocytes (John et al., 2022). In cancer, tumor cells exploit remodeled mitochondrial dynamics to promote disease progression: many tumors enhance the Warburg effect through mitochondrial fission (through DRP1 upregulation and MFN2 silencing) and maintain proliferation under hypoxia and therapeutic pressure (Chen W. et al., 2023; Wang S. F. et al., 2023). Mitochondrial fragmentation also promotes chemotherapy resistance by enhancing autophagic clearance and strengthening NF-κB-dependent pro-survival signaling pathways (Li F. et al., 2023). However, major knowledge gaps remain regarding the specific alterations and regulatory mechanisms of mitochondrial dynamics across different tumor types and treatment stages.

Many genetic diseases are caused by mutations in core mitochondrial dynamics genes, such as MFN2, OPA1, DNM1L, and GDAP1. Charcot–Marie–Tooth disease type 2A is a common peripheral neuropathy, usually caused by mutations in the MFN2 gene, resulting in impaired axonal energy supply and compromised myelin sheath integrity (Adhikary et al., 2023; Chan, 2012). Autosomal dominant optic atrophy is caused by OPA1 gene defects, leading to mitochondrial cristae structural abnormalities, impaired ATP synthesis, and progressive loss of retinal ganglion cells (Griparic et al., 2004; Chan, 2012). Although these diseases are of different types, they share common pathogenic mechanisms, including reduced oxidative phosphorylation (OXPHOS) efficiency caused by mitochondrial fragmentation (Chen W. et al., 2023), a self-amplifying cycle of damage between ROS and mitochondrial DNA (mtDNA), mitochondrial outer membrane permeabilization (MOMP) accompanied by cytochrome c release, and calcium overload associated with abnormal ER–mitochondria contacts (Wen et al., 2025). Therefore, intervention in mitochondrial dynamics-regulatory nodes, such as DRP1 phosphorylation status and MFN2/OPA1 expression levels, provides a promising strategy for the treatment of various diseases. This cross-disease disorder model highlights the potential of natural products with multifaceted and multi-target activities as mitochondrial dynamics regulators. The following section will systematically summarize the regulatory mechanisms of natural products and their translational relevance and application significance in different disease contexts.

## 4 A four-dimensional framework for natural product-mediated regulation of mitochondrial homeostasis

After explaining the core mechanisms (Section 3) and the pathological consequences caused by their dysfunction, we now explore how natural products exert therapeutic effects through coordinated system-level regulation (Figure 4). Natural products do not act on isolated nodes but function within a regulatory framework that integrates four dimensions: basic construction (mitochondrial biogenesis), morphological regulation (fission–fusion balance), quality control (mitophagy), and defense mechanisms (oxidative stress response). Importantly, these dimensions are highly interdependent and usually physically converge at organelle contact sites, such as MERCs and MLCs, to form a dynamic continuum that maintains mitochondrial homeostasis. For example, biogenesis provides the basis for morphological remodeling, while fission is a prerequisite for selectively removing damaged organelles through mitophagy.

**FIGURE 4:**
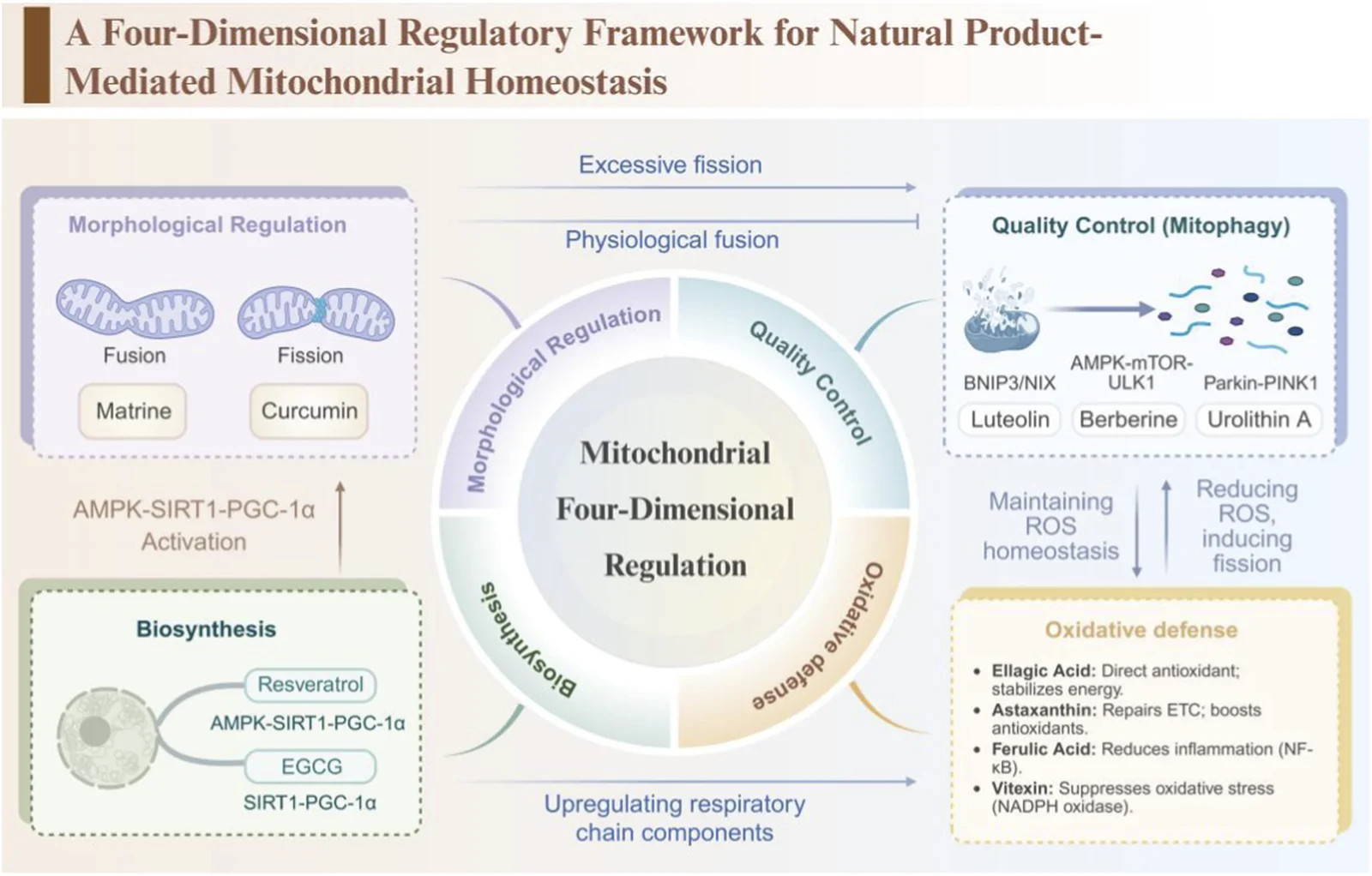
Multifaceted perspective on how natural products mediate mitochondrial homeostasis: a four-dimensional regulatory framework. This schematic presents a four-dimensional regulatory framework for mitochondrial homeostasis, comprising morphological regulation, quality control (mitophagy), oxidative defense, and biogenesis. The morphological regulation part describes the balance between fusion (mediated by matrine) and fission (mediated by curcumin), disruption of which leads to excessive fission or impaired fusion. The quality control section elaborates on the key mitophagy pathways (BNIP3/NIX and PINK1/Parkin) and their regulatory metabolites (luteolin, berberine, and urolithin A). The oxidative defense part mainly focuses on antioxidants that maintain ROS homeostasis: tannic acid (a direct antioxidant that can stabilize the bioenergetic status), astaxanthin (supports the function of the electron transport chain and enhances antioxidant defense), ferulic acid (inhibits NF-κB-mediated inflammation), and vitexin (inhibits NADPH oxidase to suppress oxidative stress). Biogenesis focuses on the AMPK/SIRT1/PGC-1α axis (activated by resveratrol and EGCG) to upregulate respiratory chain metabolites.

A core and often overlooked challenge is that the significant therapeutic effects of natural product interventions *in vitro* are often limited by unfavorable PK properties, which constitute a major obstacle to clinical translation. The concentrations required to achieve mitochondrial regulation *in vitro* are usually higher than the systemic concentrations achievable *in vivo*; therefore, their physiological relevance must be carefully evaluated. In addition, the dose-dependent characteristics of natural products are usually manifested as mitohormesis (see Section 4.5.2), which requires careful dose optimization to avoid toxicity and maximize adaptive responses. The concept of mitohormesis proposes that many natural products may induce mild mitochondrial stress at low, physiologically achievable concentrations, thereby triggering beneficial adaptive responses. This may help explain why therapeutic effects can still be observed *in vivo* despite an obvious pharmacokinetic/pharmacodynamic (PK/PD) disconnect. The following sections will explain how different chemical categories such as polyphenols, alkaloids, flavonoids, and glycosides reshape mitochondrial function, with a focus on their multidimensional synergistic effects.

### 4.1 Foundational construction: activation of mitochondrial biogenesis

This section focuses on the biogenesis dimension of our four-dimensional framework, which acts as the foundational prerequisite for subsequent morphological remodeling (Section 4.5) and mitophagy-based quality control (Section 4.6). Mitochondrial biogenesis is the basis of the dynamic network and makes subsequent morphological reshaping and quality control possible (Figure 5). Natural products usually activate this program through the AMPK/SIRT1/PGC-1α axis. This highly coordinated process involves dual-genome control, including the synthesis of mitochondrial membranes, the replication of mtDNA, and the import of nuclear-encoded proteins. AMP-activated protein kinase (AMPK), a cellular energy sensor, and the NAD^+^-dependent deacetylase SIRT1, a redox-state integrator, converge on peroxisome proliferator-activated receptor gamma coactivator 1-alpha (PGC-1α), which is activated by phosphorylation and deacetylation. Activated PGC-1α coactivates peroxisome proliferator-activated receptor gamma (PPARγ) and drives the activity of nuclear respiratory factors NRF1/2, thereby inducing mitochondrial transcription factor A (TFAM) and other nuclear-encoded mitochondrial genes (Kühlbrandt, 2015). Therefore, pharmacological regulation of the AMPK/SIRT1/PGC-1α pathway is an attractive strategy to restore mitochondrial biogenesis in diseases associated with organelle dysfunction.

**FIGURE 5:**
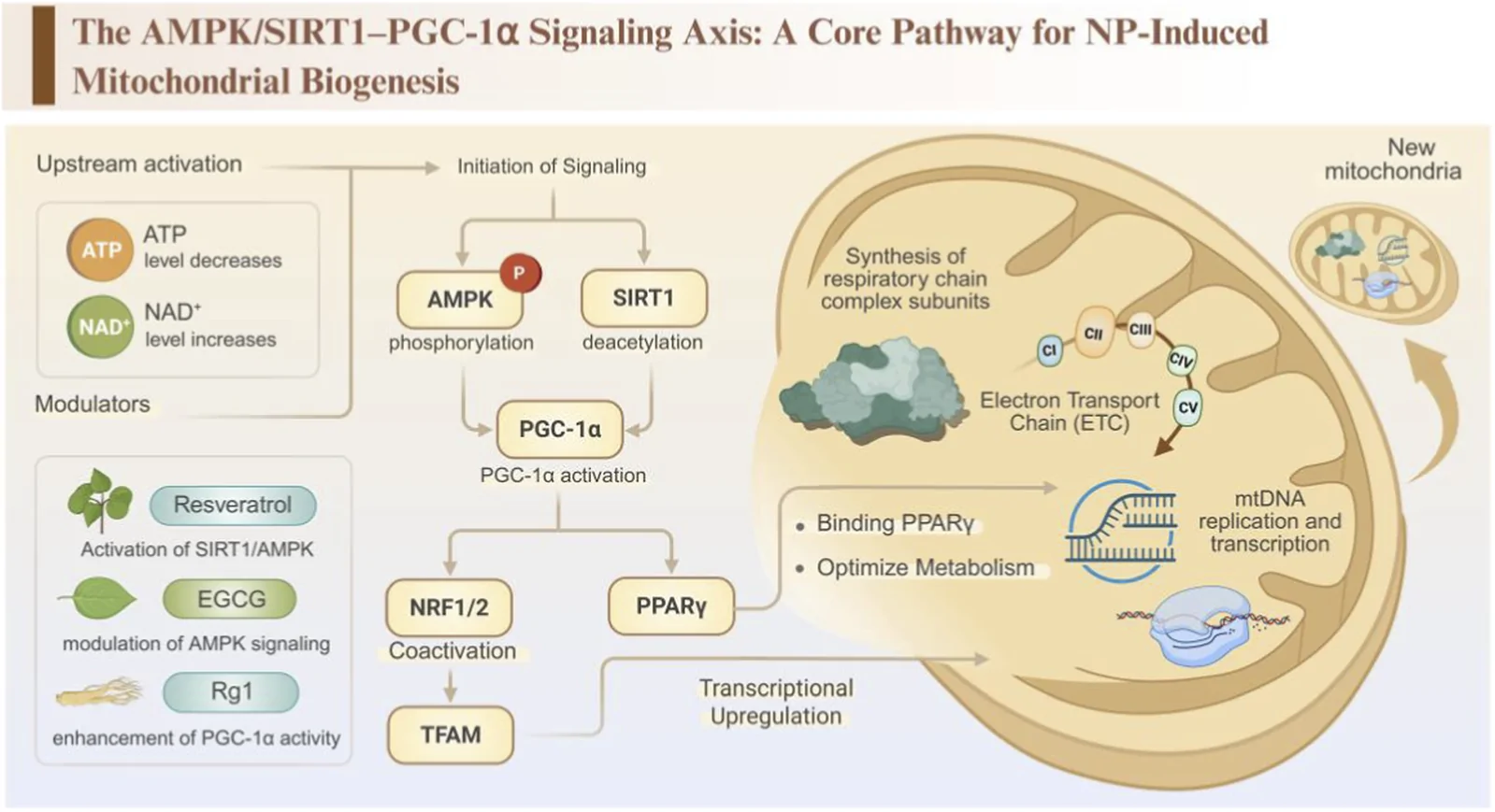
Building new powerhouses: The AMPK/SIRT1/PGC-1α signaling axis as a master regulator of natural product-induced mitochondrial biogenesis. This schematic illustrates the AMPK/SIRT1/PGC-1α signaling axis regulating mitochondrial biogenesis. Upstream activation involves decreased ATP levels leading to AMPK phosphorylation and increased NAD^+^ levels activating SIRT1 deacetylase activity. Natural metabolite modulators include resveratrol for activation of SIRT1/AMPK, epigallocatechin gallate (EGCG) for modulation of AMPK signaling, and Rg1 for enhancement of PGC-1α activity. AMPK and SIRT1 cooperate to activate PGC-1α. The activated PGC-1α binds to PPARγ to optimize metabolism and coactivates transcription factors NRF1/2, resulting in TFAM upregulation. This promotes the synthesis of respiratory chain complex subunits (CI–CV) and facilitates mitochondrial DNA replication and transcription, thus driving the formation of new mitochondria.

### 4.2 Polyphenols activate mitochondrial biogenesis via the AMPK/SIRT1/PGC-1α axis

Among natural products, polyphenols are the most widely studied inducers of mitochondrial biogenesis and often act as dual activators of AMPK and SIRT1. EGCG is a catechin-type polyphenol and a major bioactive metabolite extracted from fresh young leaves of the green tea plant *Camellia sinensis* (L.) Kuntze. It has been reported to enhance the activity of AMPK and SIRT1 and promote mitochondrial biogenesis. For example, in cells derived from patients with Down syndrome, EGCG (5 μM) enhances mitochondrial biogenesis through SIRT1-dependent deacetylation of PGC-1α, which is consistent with activation of the PGC-1α/NRF1/NRF2/TFAM cascade, thereby enhancing OXPHOS efficiency (Valenti et al., 2013). However, as articulated in the Core Paradox (Introduction), this *in vitro* concentration exemplifies the fundamental translational challenge: the oral bioavailability of EGCG is negligible (<1%), and human pharmacokinetic analysis shows that even at an extremely high dose (8 g/day), the metabolite reaches only a low-nanomolar maximum plasma concentration (C_max_). By contrast, the >10 μM threshold required for measurable SIRT1 activation *in vitro* is more than an order of magnitude higher (Andreu Fernández et al., 2020). This significant difference in concentration strongly indicates that while the direct activation of SIRT1 is unlikely to be the main mechanism for the benefits of EGCG, other mechanisms may play a role at nanomolar concentrations, such as mitohormetic adaptation induced by mild oxidative stress or the action of microbiota-derived metabolites (see Sections 4.3, 4.5.2, and 5).

Resveratrol (a polyphenolic monomer isolated from *Polygonum cuspidatum* Sieb. and Zucc.) stimulates the AMPK/SIRT1/PGC-1α axis through related mechanisms, partly because it promotes electron transport chain (ETC) flux, thus increasing the NAD^+^/NADH ratio, which in turn triggers mitochondrial biogenesis (Kapetanovic et al., 2010; Xie et al., 2023), and restores oxidative phosphorylation efficiency in the Down syndrome model. Resveratrol works in the concentration range of 10–50 μM. In this concentration range, it can upregulate MFN2, promote fusion, and enhance mitochondrial function in podocyte and neuronal models (Martínez-Abundis et al., 2016). Consistent with the Core Paradox (Introduction), these concentrations are difficult to achieve systemically: rapid metabolism limits oral bioavailability to approximately 6%, and although resveratrol penetrates the blood–brain barrier (BBB) better than EGCG, the free aglycone concentrations remain far below the *in vitro* effective range (see Section 5.1.1). However, the direct molecular target of resveratrol is still controversial: some studies attribute its AMPK activation to phosphodiesterase inhibition (an indirect cAMP/PKA-mediated mechanism), while other studies suggest that it directly binds to AMPK subunits (Cerqueira et al., 2025). In addition, the effect of resveratrol on SIRT1 is highly context-dependent: in cells with high NAD^+^/NADH ratios (for example, under nutrient-rich conditions), it can effectively deacetylate PGC-1α. However, in energy-depleted cells (low NAD^+^ levels), its efficacy is significantly reduced (Meza-Torres et al., 2020). In addition to genetic disease models, resveratrol can also enhance biogenesis and improve mitochondrial function in experimental models of Parkinson disease and AD; its multifunctional properties include antioxidant, anti-inflammatory, and anticancer activities. In diabetic nephropathy, resveratrol inhibits apoptosis of podocytes through the SIRT1/PGC-1α pathway, which shows cross-disease applicability (Zhang et al., 2019). An unresolved key problem is how to prioritize mitochondrial biogenesis over other SIRT1/AMPK-mediated processes (such as fatty acid oxidation). In podocytes, it drives mitochondrial biogenesis, while in hepatocytes, it enhances lipid metabolism, which suggests a tissue-specific pathway preference, but its mechanism remains unclear.

### 4.3 Glycosides and alkaloids regulate mitochondrial biogenesis and coordinate quality control via the AMPK/SIRT1/PGC-1α axis

In addition to polyphenolic metabolites, glycosides and alkaloids also participate in this core biological pathway. Ginsenoside Rg1 enhances the expression of cytochrome c oxidase, increases mitochondrial membrane potential, and enhances ATP production. At the same time, by upregulating PGC-1α, NRF1, and TFAM, it transcriptionally reprograms mitochondrial bioenergetics (Kwan et al., 2021), thus conferring neuroprotection. In neuroprotection models, ginsenoside Rg1 acts in the 10–20 μM range, increasing mitochondrial membrane potential and promoting ATP synthesis (Gao et al., 2017). However, it is not clear how ginsenoside Rg1 avoids excessive biogenesis (which may increase ROS production). It simultaneously upregulates antioxidant enzymes (such as SOD2), but the coordination between biogenesis and oxidative defense has not been characterized.

Anthraquinones such as rhein and protoberberine alkaloids, which are widely derived from traditional Chinese medicine, promote mitochondrial biogenesis by activating SIRT1/PGC-1α and relieve dysfunction caused by hyperglycemia, lipid toxicity, or oxidative stress. Berberine (an isoquinoline alkaloid monomer purified from *Coptis chinensis* Franch) acts at a concentration of 1 μM in the hippocampal neuron model to resist Aβ-induced mitochondrial damage, thus increasing the AMP/ATP ratio to promote mitochondrial biogenesis (Zhao et al., 2019). While this concentration appears modest compared to other compounds, the Core Paradox (Introduction) remains salient: berberine’s oral bioavailability is extremely low (0.37–1%), and under high-dose systemic exposure, a risk of cardiotoxicity can be produced by blocking the hERG channel (Section 5.2.7). There are contradictory data on the mechanism of AMPK activation: some studies have shown that it inhibits complex I to increase the AMP/ATP ratio (thereby directly activating AMPK through energy stress), while others report that it plays an indirect role through LKB1 phosphorylation (upstream kinase activation) (Feng et al., 2021), which complicates interpretation of its therapeutic effects in different cell types and energetic states. Similarly, at a concentration of 10–20 μM, rhein, an anthraquinone metabolite extracted from the dried rhizome of *Rheum officinale* Baill. (family Polygonaceae), can promote mitochondrial biogenesis and maintain mitochondrial mass in hyperglycemia/lipid toxic models, forming a dynamic mass-control system (Yin et al., 2022). By simultaneously inducing biogenesis (through SIRT1/PGC-1α) and regulating autophagy-related proteins, rhein plays a wider regulatory role, thus strengthening the concept of mitochondrial mass monitoring as a dynamic coupling system. This reflects a key principle: the crosstalk between mitochondrial biogenesis and mitochondrial quality control constructs a closed-loop regulatory system that balances energy production and damaged organelle clearance. However, the mechanism of coordination of SIRT1/PGC-1α (biogenesis) and Beclin1/LC3 (autophagy) is still unclear. At present, no study has yet identified the upstream signal hub connecting these two processes, which constitutes an important knowledge gap.

This highlights the interaction between biogenesis and quality control in the mitochondrial life cycle. These natural products with different structures have built a dual support network of biogenesis and quality control through multi-target and multi-level coordination. In line with this paradigm, echinacoside, a phenylethanoid glycoside from *Echinacea purpurea* (Asteraceae), improves the quality and function of mitochondria in the aging model by activating the AMPK signaling pathway. In the aging model, echinacoside can significantly increase the number of mitochondria and improve mitochondrial function at a concentration of 50–100 μM (Zhou et al., 2020). However, the oral bioavailability of echinacoside is extremely low (∼0.83%), and its ability to induce biogenesis *in vivo* (compared with *in vitro*) has not been confirmed, which limits its translational relevance (Liu et al., 2018). These examples emphasize the extensive convergence of a variety of natural products in the AMPK/SIRT1/PGC-1α axis and highlight their significant heterogeneity in terms of mechanism, efficacy, and pharmacokinetics, which affect clinical applications and formulation strategies.

### 4.4 Efficacy and comparative mechanisms of key biogenesis activators

The AMPK/SIRT1/PGC-1α axis has been established as the core signaling pathway of mitochondrial biogenesis. Although a variety of natural products, including polyphenols (such as EGCG and resveratrol), glycosides (such as ginsenoside Rg1), and alkaloids (such as berberine), converge on this axis, they differ substantially in mechanisms of action, efficacy profiles, and therapeutic relevance. Importantly, these differences are further aggravated by significant PK limitations and mechanistic controversy, thus challenging their translational potential.

Polyphenols usually work quickly by regulating upstream co-factors. For example, EGCG promotes SIRT1-dependent PGC-1α deacetylation, which may help address genetic vulnerabilities such as Down syndrome. However, its extremely low oral bioavailability (<1%) and maximum plasma concentrations in the low-nanomolar range, far below the >10 μM required to activate SIRT1 *in vitro*, strongly indicate that its *in vivo* benefits are unlikely to be directly mediated by SIRT1. Instead, other mechanisms, such as mitohormetic adaptation or the action of microbial metabolites, may be more important (Sections 4.6.4 and 4.5.2). In addition, EGCG has the dual effects of antioxidant and pro-oxidant activity; at high doses (>50 μM), it can disrupt the ETC and induce the production of ROS, making dose selection more complicated (Hazimeh et al., 2023). Resveratrol strongly triggers biogenesis by promoting electron transport chain flux and increasing the NAD^+^/NADH ratio, which is especially important in energy-deficient neurons. However, its mechanism remains controversial: some studies believe that AMPK activation is caused by phosphodiesterase inhibition and subsequent cAMP/PKA signaling, while other studies suggest that resveratrol directly binds to AMPK subunits (Cerqueira et al., 2025).

In addition, resveratrol’s impact on SIRT1 largely depends on the specific context: under nutrient-replete conditions, its effect is significant, whereas in cells with insufficient energy, its effect is weaker. There are also conflicting research results regarding its tissue permeability: some studies show that it can pass through the BBB, while others report that it cannot be detected in brain tissue, which greatly limits its application in the treatment of neurodegenerative diseases (Sergides et al., 2016). In general, the mechanisms of action of polyphenolic metabolites are relatively clear and act quickly, but their pharmacokinetic properties are poor, including low bioavailability, rapid metabolism, and a narrow therapeutic window, which limits their clinical application.

Unlike the rapid onset of polyphenols, glycosidic metabolites such as ginsenoside Rg1 usually exert milder and more sustained effects. Ginsenoside Rg1 may cooperate with TFAM to upregulate respiratory chain metabolites, such as cytochrome c oxidase, thereby improving biogenesis efficiency. Its active metabolite, protopanaxatriol (PPT), can prolong pharmacological activity, with a half-life of approximately 24 h. However, due to P-glycoprotein (P-gp) efflux and first-pass metabolism, the oral bioavailability of parent metabolites is low (1.9–20%) (Inam et al., 2017). A key knowledge gap is whether PPT retains the biological activity of Rg1. Most studies focus on parent metabolites, and the contribution of metabolites to the therapeutic effect *in vivo* is still unclear. In terms of neuroprotection, this mild and sustained activation may be conducive to the maintenance of mitochondria. However, the mitochondrial specificity of ginsenoside Rg1 is not yet clear; it also regulates other pathways (such as the PI3K/AKT pathway), which may complicate interpretation of its mitochondrial biogenesis. Alkaloids such as berberine adopt more active strategies, mainly by inhibiting complex I to increase the AMP/ATP ratio and strongly activate AMPK. This mechanism is effective in severe energy imbalances (such as insulin resistance), but its impact on OXPHOS remains contradictory. Some studies show that biogenesis can improve the function of complex I, while other studies report that at therapeutic doses, Complex I is still inhibited, which raises concerns about impaired energy metabolism (Feng et al., 2021). Its therapeutic window is relatively narrow; higher doses may lead to gastrointestinal intolerance and increase the risk of hepatotoxicity and hERG channel blockade-associated cardiotoxicity (see Section 5.2.7). This cell-type specificity is particularly important: although berberine is beneficial to insulin-resistant hepatocytes, it may aggravate mitochondrial complex I-dependent cardiomyocyte dysfunction, so its cell-type-specific effects must be evaluated.

There may be synergy between different types of natural products. The combination of EGCG and Rg1 may have a complementary effect: EGCG can quickly activate relevant pathways, while Rg1 provides sustained support. However, there is a potential risk of interaction with high doses of EGCG, so such combinations should be used with caution. At present, such combinations have not been systematically evaluated. Existing research is mainly focused on single metabolites, so the opportunity to use complementary mechanisms is missed.

Similarly, given the strong impact of berberine on cellular energy metabolism, strict safety assessment is also required when it is combined with other biogenesis activators. In practice, polyphenolic metabolites are most suitable for rapid intervention and local delivery or formulation-assisted absorption; glycosides are more suitable for low-intensity supportive treatment in neurodegenerative diseases; and alkaloid metabolites can be used to treat acute energy crises in metabolic diseases under strict monitoring. However, the lack of direct comparison research across disease models is still a major knowledge gap. For example, whether resveratrol or ginsenoside Rg1 is more effective in AD, and why, remains unresolved.

The activation of mitochondrial biogenesis lays the foundation for the construction of a robust organelle network, but cell homeostasis also depends on the stability and dynamic balance of mitochondrial morphology. As structural reshaping and functional regulation promote each other, we must now turn to the regulation of mitochondrial morphology, especially the balance required to maintain network integrity while controlling fission, and clearly consider the regulatory mechanisms and context dependence of this process.

### 4.5 Morphological regulation of rebalancing the fission–fusion equilibrium

Based on the basic role of biogenesis, morphological regulation directly determines the functional capacity of mitochondrial networks. Dysregulation at this level is a root cause of many diseases and has therefore become an important target of natural product intervention. Mitochondrial form and function are maintained by a continuous fission–fusion balance: fusion promotes content mixing, complementation, and repair, whereas fission separates damaged regions and controls organelle distribution. Fission is regulated by DRP1, while fusion is mediated by MFN1/MFN2 on the outer mitochondrial membrane and OPA1 on the inner membrane. Natural products can rebalance the DRP1/OPA1/MFN1/2 axis by coordinating post-translational modifications and gene-expression programs, thereby correcting dysregulated mitochondrial dynamics. Notably, this rebalancing often occurs at the physical interface of MERCs, which serve as both the initiation sites of mitochondrial fission and enrichment areas for fusion proteins such as MFN2. Therefore, natural product intervention at MERCs can profoundly affect mitochondrial morphology and inter-organelle signaling.

#### 4.5.1 DRP1/OPA1 dual pathway regulating fission–fusion balance

This subsection covers the Morphological Regulation dimension of our four-dimensional framework; balanced mitochondrial fission–fusion controlled by DRP1 and OPA1 is tightly coupled to subsequent mitophagy-based quality control (Section 4.6). Strict control of fusion and fission is central to mitochondrial network homeostasis. Natural products restore this balance by targeting nodal regulators such as DRP1 and OPA1. Polyphenolic metabolites show significant benefits in hepatic and metabolic contexts, particularly through multi-target mechanisms. Curcumin, a major active metabolite of turmeric rhizomes, directly acts on core metabolites of the fission–fusion machinery and exerts fine control over DRP1 activity in cell and animal models (Hasan et al., 2019). Evidence shows that curcumin, a curcuminoid polyphenol metabolite extracted from the dried rhizome of *Curcuma longa* L. (family Zingiberaceae), directly relieves mitochondrial fragmentation and stabilizes cristae at 5–50 μM in alcoholic liver disease (ALD)/NAFLD models (Wang B. et al., 2023; Khatoon and Kalam, 2025; Liu et al., 2022). These *in vitro* effective concentrations vastly exceed achievable systemic Cmax (∼1.7 µM even with an 8 g oral dose), which is a stark exemplification of the Core Paradox (Introduction). Without an advanced delivery system, these mechanisms may not be clinically significant, as detailed in Section 5.1.1. However, contradictory reports about the main mechanism of action of curcumin in different tissues exist: in cardiomyocytes, curcumin reduces the phosphorylation level of Ser616 by inhibiting CDK5, whereas in hepatocytes, activation of AMPK enhances Ser637 phosphorylation (Xu et al., 2013), suggesting unresolved context-dependent differences in post-translational modification preferences. At the same time, curcumin can activate the mitochondrial deacetylase SIRT3, thereby deacetylating OPA1, enhancing mitochondrial fusion, stabilizing the cristae structure, and promoting the assembly of respiratory chain supercomplexes (Ryu et al., 2024). This dual regulatory effect of inhibiting fission and enhancing fusion is consistent with the therapeutic effects of curcumin in ALD and NAFLD (Xu et al., 2013). However, the ability of curcumin to restore cristae structure differs between ALD and NAFLD. In ALD, curcumin can reverse OPA1 cleavage, while in NAFLD, curcumin promotes the assembly of respiratory chain supercomplexes. This highlights as-yet-unexplained disease-specific mechanisms.

Resveratrol is a non-flavonoid polyphenol, abundant in *Polygonum cuspidatum*, and it complements the mechanism of action of curcumin. At 10–50 μM, it increases MFN2 expression to drive mitochondrial elongation and stabilize MERCs in podocyte models (see Section 4.1) (Ren et al., 2017; Meza-Torres et al., 2020; Wang B. et al., 2023; Martínez-Abundis et al., 2016). Whether this MFN2 upregulation relies on PGC-1α remains controversial, as recent work identifies PGC-1α-independent regulatory pathways for resveratrol (Cerqueira et al., 2025). Although the AMPK/SIRT1/PGC-1α axis plays an important role in a variety of contexts, from podocyte protection in diabetic nephropathy to lipid oxidation in obesity-related metabolic syndrome, the specific transmission mechanism of this signaling axis to particular morphological effectors remains unclear. Punicalagin (a hydrolyzable ellagitannin isolated from the dried pericarp of *Punica granatum* L.), the major polyphenol in pomegranate peel, can enhance mitochondrial supercomplex function and correct energy imbalance through synergy with resveratrol via the STAT3/PTP1B axis. Its therapeutic effect has been confirmed in inflammatory bowel disease and hypoxic-ischemic brain injury. In cardiomyocyte models, punicalagin can play a role at a concentration of 10 μM; in the model of diabetic rats, the dose of 90 mg/kg/day can improve the energy metabolism imbalance (Wang Y. H. et al., 2023; Fu et al., 2021). Note that the bioavailability of punicalagin itself is extremely low, and its *in vivo* effect is mainly mediated by the metabolite of gut microbiota, urolithin A (Section 4.6.4). A key knowledge gap is whether this synergy extends to fission–fusion regulation: although punicalagin can improve the supercomplex function, its impact on DRP1/OPA1 has not been reported, which limits our understanding of its complete morphological effects. Compared with curcumin, which mainly acts on protein-level modification, the effect of resveratrol acts further upstream within signaling cascades and finally achieves transcriptional regulation, but the relative importance of these two modes (post-translational regulation and transcriptional regulation) in recovery kinetics is still unclear.

In the cardiovascular model, rosmarinic acid (a phenolic acid metabolite derived from *Rosmarinus officinalis* L. and *Perilla frutescens*) showed dose-dependent effects in the concentration range of 10–100 μM; while in the diabetic rat model, chicoric acid (a phenolic acid metabolite derived from *Cichorium intybus* L. and *Echinacea* spp.) can further balance mitochondrial fission–fusion at a dose of 50 mg/kg and protect cardiomyocytes (Ding et al., 2022; Gupta et al., 2022). However, there is no direct comparison of their efficacy in different cardiovascular disease models: rosmarinic acid is effective for ischemia/reperfusion injury, and chicoric acid is effective for diabetic cardiomyopathy, but it is not clear whether this reflects the disease-specific mechanism or the off-target effect. Paeonol is a phenolic metabolite derived from the cortex of *Paeonia suffruticosa*. By activating the PKCε/STAT3 pathway, it significantly improves the transcription and protein levels of MFN2 and enhances mitochondrial fusion, thus playing a significant protective role in the cardiotoxicity and myocardial injury models induced by doxorubicin. In the myocardial cell model, paeonol promotes mitochondrial fusion in the concentration range of 10–50 μM (Chen et al., 2018; Hu et al., 2020; Ding et al., 2023). Regarding the effect of paeonol on DRP1, the existing data are contradictory: some studies show that it can reduce the expression of DRP1, while others report no effect, suggesting that MFN2 upregulation may be its main mechanism, but this has not yet been formally verified by DRP1 overexpression experiments. From direct protein-level modulation (rosmarinic acid/chicoric acid) to transcription factor-driven gene activation (paeonol), this process constitutes a complementary layer of the heart protection mechanism.

In neurodegenerative diseases, isochlorogenic acid A (a chlorogenic acid-type phenolic acid metabolite derived from *Coffea arabica* L., Asteraceae plants, and *Lonicera* species) can reduce the expression of DRP1 and its outer mitochondrial membrane receptor FIS1, and at the same time upregulate the expression of OPA1, MFN1, and MFN2, thus inhibiting mitochondrial fission, promoting mitochondrial fusion, and quickly restoring the integrity of the mitochondrial network. However, there is a significant difference in its efficacy in AD and Parkinson disease models: in the AD model, it can reduce the activation of Aβ-induced DRP1, while in the Parkinson disease model, it has no effect on the fission mediated by α-synuclein. This highlights the unresolved disease-specific vulnerability. Gastrodin, derived from the tuber of *Gastrodia elata,* enhances mitochondrial fusion by increasing the nuclear translocation of NRF2, which drives the expression of fusion mediators (MFN1/2 and OPA1). The transcription program provides a continuous neuroprotective effect and reduces vascular dementia and neuronal apoptosis in the ischemic stroke model. In the stroke model, gastrodin played a role in the dose range of 100–200 mg/kg, promoting mitochondrial fusion and providing long-term protection at the transcriptional level (Cheng et al., 2020; Chen Y. X. et al., 2024). An unresolved key problem is how NRF2, which is traditionally considered an antioxidant transcription factor, regulates fusion genes: direct NRF2 binding sites have not been found in the promoters of MFN1/2 or OPA1, which suggests that NRF2 may act indirectly through as-yet undefined intermediate factors. Therefore, protein-level intervention and transcriptional reprogramming work together to protect mitochondrial structure and maintain its function in various diseases.

#### 4.5.2 Synergistic role of gene regulation and ion homeostasis

Alkaloid metabolites recalibrate mitochondrial dynamics by regulating gene-expression programs and ion homeostasis, thus showing therapeutic potential in different disease contexts. Matrine is a quinolizidine alkaloid extracted from the root of *Sophora flavescens* (Fabaceae). Its mechanism of action is different from that of the polyphenolic metabolite curcumin. Under stress conditions based on Ca^2+^-overload (usually caused by excessive MERCs signaling), matrine inhibits the excessive activation of the inner-membrane metalloproteinase OMA1, thus preventing the collapse of mitochondrial membrane potential (ΔΨm) and limiting cleavage of the long, fusion-competent OPA1 isoform. By protecting L-OPA1, it maintains mitochondrial fusion ability, reduces mitochondrial fragmentation (You et al., 2020), and enhances the protective effect of organelles in models such as renal ischemia–reperfusion injury. In the kidney injury model, intravenous injection of matrine (6 mg/kg) can maintain the fusion state by inhibiting the Ca^2+^/OMA1 axis and reducing L-OPA1 cleavage (Feng et al., 2024). However, the therapeutic effect of matrine in cells with abnormal Ca^2+^ homeostasis is weakened. In the chronic kidney disease (CKD) model, the increase in the level of basic Ca^2+^ will damage its ability to maintain L-OPA1, indicating that its activity window is narrow and affected by ion dependence. This factor has not been included in the drug administration strategy. Complementing this ion-centered mechanism, the indole alkaloid flazin counteracts L-buthionine-(S,R)-sulfoximine (BSO)-induced mitochondrial fragmentation by upregulating the fusion mediators MFN1/2 and inhibiting the fission effector DRP1. This transcriptional remodeling can restore mitochondrial branches, increase the network size, enhance fusion, improve bioenergetic function, and ultimately increase ATP generation while reducing BSO-induced cell death. In the oxidative stress model, flazin showed a concentration-dependent effect in the concentration range of 10–50 μM, inhibiting mitochondrial fragmentation and restoring network structure (Wu et al., 2023). A key knowledge gap is whether the transcriptional effect of flazin depends on Ca^2+^: unlike matrine, flazin does not target OMA1, and its therapeutic effect in the Ca^2+^-overload model has not been reported, which makes its effectiveness in ion-driven diseases (such as ischemia–reperfusion) unclear. In short, matrine gives priority to maintaining the integrity of the Ca^2+^ steady state and OPA1, while flazin enhances the fusion state through transcription and reprogramming. However, there is no research on whether these two mechanisms can be combined to solve the problem of mixed ion/transcription defects.

Cocaine is an alkaloid extracted from coca leaves. It extends the discussion to the field of neuropsychiatric diseases by activating a unique transcription pathway. It enhances the binding of early growth response 3 (EGR3) to the nuclear gene that controls mitochondrial function and dynamics, thus promoting the upregulation of transcription coactivator PGC-1α and fission protein DRP1. In animal models, cocaine can be administered at a dose of 20 mg/kg/day, inducing neuroadaptation and behavioral changes by upregulating PGC-1α and DRP1 and thereby changing the balance between mitochondrial fission and fusion (Cole et al., 2021). This EGR3-centered signaling cascade provides a mechanistic basis for cocaine-induced neural adaptation and related behavioral phenotypes. However, the ability of any natural-product-based intervention to inhibit EGR3-mediated DRP1 upregulation has not been evaluated; this represents a target that has not been fully explored in the treatment of substance use disorders.

The regulation of mitochondrial dynamics is not limited to classical fission–fusion proteins but also involves calcium signaling and specific transcriptional networks. Natural products that regulate these pathways may provide customized interventions for specific pathological conditions. They also highlight the need for a deeper understanding of cellular signaling.

#### 4.5.3 Targeted intervention at key fission–fusion nodes

Flavonoids protect mitochondrial function by interfering with the key regulatory points of the mitochondrial fission–fusion loop. Vitexin comes from *Vitex negundo* L. (Lamiaceae). It alleviates mitochondrial dysfunction by enhancing the expression of MFN2 and inhibiting DRP1 at the same time. Its regulatory effect is similar to that of metabolites such as paeonol and isochlorogenic acid A. In myocardial ischemia–reperfusion and hypoxia–reoxygenation models, vitexin acts at 10 μM to reduce ROS generation at the source and maintain oxidative phosphorylation, significantly improving ATP synthesis (Duan et al., 2024). The Core Paradox (Introduction) applies here as well: while 10 µM is achievable in localized cardiac tissue under certain delivery scenarios, systemic oral administration is unlikely to reach this threshold, underscoring the need for targeted formulation strategies (Section 5.1). However, the glycosylated state of vitexin affects its activity: deglycosylated vitexin loses the ability to upregulate MFN2, indicating that the glycosyl moiety is crucial for target binding, although no study has yet described the interaction between glycosylated vitexin and its presumed targets (such as transcription factors or kinases). In inflammatory models, quercetin (a flavonol metabolite widely distributed in various plants, including *Sophora japonica* L. and *Vitis vinifera* L.) acts in the concentration range of 5–20 μM, inhibiting mitochondrial fission and promoting mitochondrial fusion (Vafadar et al., 2020; Li F. et al., 2021). However, invoking the Core Paradox (Introduction), the C_max_ achievable in human plasma is only ∼2.3 μg/mL (∼7.6 μM for quercetin aglycone), with limited brain penetration, raising questions about the physiological relevance of these *in vitro* concentrations for central nervous system applications. Currently, findings regarding the effect of quercetin on DRP1 phosphorylation are contradictory: some studies show that quercetin can reduce the phosphorylation at Ser616, while other studies have not observed this effect, suggesting that PVT1 may act through non-phosphorylation mechanisms, such as DRP1 ubiquitination.

Baicalein (a flavonoid metabolite extracted from the dried root of *Scutellaria baicalensis* Georgi) and sanggenon C (a flavonoid metabolite extracted from the dried root bark of *Morus alba* L.) play complementary regulatory roles, forming a regulatory loop between transcription and post-translational regulation. Baicalein is a polyhydroxyflavonoid that can regulate the KLF4/MARCH5 axis, thus affecting the ubiquitination of MFN proteins and DRP1-related factors, inhibiting mitochondrial fission and stabilizing membrane potential (Hu et al., 2022; de Oliveira et al., 2015). In the hepatocyte model, the effective concentration of baicalein is approximately 10 μM; at this concentration, it can reduce mitochondrial fission, maintain autophagic homeostasis, and inhibit apoptosis. MARCH5 is a key mitochondrial E3 ubiquitin ligase that can regulate the turnover of mitochondrial dynamics-related proteins.

However, the specificity of Baicalein for MARCH5 is still unclear, because it may also regulate other E3 ligases, such as Parkin, which raises concerns about its off-target effects on mitophagy. At the same time, baicalein optimizes autophagic flux, reducing p62/SQSTM1 while increasing Beclin-1 and LC3-II, and inhibits the apoptotic cascade of Bax/caspase-3. In the CCl4-induced liver injury model, a dose of 50 mg/kg reduces mitochondrial fission and ROS (increasing superoxide dismutase (SOD), catalase (CAT), and GSH-Px activity), improves liver function (reducing ALT, AST, DBIL, and TBIL), and reduces fibrosis (Yu et al., 2020). Its neuroprotective effects under oxidative stress and its potential utility as an oncological adjuvant further highlight its broad therapeutic potential (Hu et al., 2022; de Andrade Teles et al., 2018).

Sanggenon C is isolated from *Morus alba* root bark and inhibits excessive fission by preventing overactivation of the ERK1/2 arm of the MAPK pathway. Because ERK can directly phosphorylate DRP1 at Ser616 or indirectly alter DRP1 activity and localization through upstream effectors, inhibition of ERK signaling by sanggenon C is likely to suppress DRP1-mediated fission. In gastric cancer cell models of proliferation and mitochondrial fission, sanggenon C acts at a concentration of approximately 9 μM, significantly inhibiting tumor cell proliferation and colony formation by suppressing excessive fission, while also inducing G0/G1 phase arrest. However, MAPK signaling is highly context-dependent: in some cancer cells, ERK inhibition can reduce cell proliferation independently of DRP1, which complicates the explanation of the anticancer efficacy of sanggenon C (Chen et al., 2022). Therefore, the precise molecular relay by which natural products recalibrate mitochondrial dynamics through this pathway warrants further investigation. In addition to mitochondrial remodeling, sanggenon C also downregulates BCL-2, upregulates BAX, activates caspase-9 and caspase-3, and induces apoptosis in gastric cancer cells. *In vitro* (HGC-27 and AGS) and *in vivo* (nude-mouse xenograft) studies show that an IC_50_ of approximately 9 μM is associated with strong antitumor activity, inhibition of proliferation and colony formation, G0/G1 arrest, and reduced tumor volume, with no obvious organ toxicity (Chen et al., 2022). Together, these flavonoids illustrate node-targeting effects across transcription factors, E3 ligases, and kinase pathways to restore fission–fusion balance and maintain mitochondrial network integrity. However, the frequent disconnect between strong *in vitro* efficacy and low *in vivo* bioavailability remains a major obstacle to the therapeutic development of such natural products.

#### 4.5.4 Complementary optimization mechanisms for dynamic networks

Glycoside natural products refine mitochondrial dynamics through complementary and context-dependent mechanisms. Salidroside is a phenylethanoid glycoside abundant in the roots and rhizomes of *Rhodiola crenulata* (Crassulaceae). It reshapes mitochondrial morphology through the AMPK/DRP1/MFN1/2 pathway and alleviates mitochondrial damage at a 50 mg/kg dosage in cerebral ischemia rat models (Zhang et al., 2021; Chen et al., 2017; Wen et al., 2022). In diabetic vascular lesions, DRP1 mRNA and protein are reduced in a dose-dependent manner, MFN2 is upregulated, the proportion of filamentous mitochondria is increased, and high glucose-induced proliferation of vascular smooth muscle cells is inhibited; these effects are associated with reduced ROS and NADPH oxidase activity and are reversible by MFN2-siRNA (Zhuang et al., 2017).

Salidroside’s mechanism of action may vary depending on the pathological background: in cerebral ischemia, regulation of the AMPK axis plays a leading role, whereas in diabetic vascular lesions, MFN2–ROS coupling is crucial. *Rhodiola rosea* has good *in vitro* safety (with no toxicity detected at concentrations below 500 μM) and multi-target effects; it shows translational potential in ischemic diseases and diabetes-related vascular lesions. Its anti-mitochondrial fragmentation activity complements the effect of another phenylethanol glycoside metabolite, echinacoside, which has also been confirmed to reduce mitochondrial fragmentation (Zhou et al., 2020). Similarly, ginsenoside Rg1, extracted from the roots, stems, leaves, and flower buds of ginseng, can reduce mitochondrial fragmentation and promote energy metabolism by increasing mitochondrial length and upregulating MFN2 expression. In the myocardial cell model, ginsenoside Rg1 can exert its effect at a concentration of 12.5 μM, significantly reducing the proportion of mitochondrial fragmentation and improving energy metabolism (Dong et al., 2016). Compared with the corresponding aglycones, glycosides usually have better solubility and pharmacokinetic properties, but their effects on mitochondrial dynamics are usually indirect and mediated by upstream signaling pathways. This highlights the importance of considering parent metabolites and their metabolites simultaneously when evaluating therapeutic potential.

#### 4.5.5 Targeting profiles and comparative efficacy of morphodynamic regulators

Natural products restore the balance between DRP1-mediated mitochondrial fission and OPA1/MFN2-mediated mitochondrial fusion through direct protein binding and/or transcriptional reprogramming. As shown in Table 1, their therapeutic effect largely depends on their chemical structure and pharmacokinetic properties.

**TABLE 1:** Summary of regulatory mechanisms and characteristics of representative natural products regulating mitochondrial fission/fusion balance.

Category	Core advantage	Main limitation	Main applicable context	References
Polyphenols	Bidirectional regulation (e.g., curcumin inhibits DRP1 and promotes OPA1)	Poor pharmacokinetics (e.g., curcumin <1%, resveratrol ∼6%)	Neurodegenerative diseases, metabolic syndrome; they often require nanodelivery	Ryu et al. (2024), Jelezova et al. (2015), Wang B. et al. (2023), Martínez-Abundis et al. (2016), Sergides et al. (2016), Kapetanovic et al. (2010)
Alkaloids	Pathological microenvironment responsiveness (e.g., matrine targets the Ca^2+^/OMA1 axis)	Narrow therapeutic window (e.g., more than 10 g of crude drug may cause hepatotoxicity)	Ischemia–reperfusion injury	You et al. (2020)
Flavonoids	Membrane targeting (sanggenon C has a logP = 3.48)	Hemolysis risk at high concentration (e.g., luteolin >20 μM)	Cardiovascular antioxidant therapy	Langeder et al. (2023), Vongthip et al. (2024)
Glycosides	High safety (e.g., salidroside has a LD_50_ > 5 g/kg) and direct DRP1 repression	Poor blood–brain barrier penetration (e.g., echinacoside has a bioavailability of F ∼ 0.83%) impedes neuroprotective applications	Cardiovascular and metabolic diseases	Chen et al. (2017), Zhang et al. (2021), Liu et al. (2018)

This table compares molecular strategies and limitations of natural products in regulating mitochondrial fission/fusion equilibrium. Aligned with the morphodynamics dimension of the four-dimensional regulatory framework, polyphenols (e.g., curcumin) suppress pathological fission through bidirectional modulation of DRP1 activity and SIRT3/OPA1 pathways, yet face bioavailability constraints (≤1%). Alkaloids (e.g., matrine) inhibit OMA1-mediated L-OPA1 cleavage in a Ca^2+^ homeostasis-dependent manner, restricting efficacy to specific pathological microenvironments. Flavonoids (e.g., vitexin) attenuate ROS by suppressing NOX4 via the Epac1/Rap1 axis, where glycosylation enhances solubility but limits CNS penetration. Glycosides (e.g., salidroside) demonstrate high safety (LD_50_ > 5 g/kg) and direct DRP1 repression, yet poor blood–brain barrier permeation (F ∼ 0.83%) impedes neuroprotective applications. Synergistic approaches are detailed in Section 5.1.2 (gut microbiota-mediated metabolic conversion). (Data originate from Section 4.5.5 and original table content).

Among different types of natural products, polyphenolic metabolites, such as curcumin, resveratrol, and paeonol, have different mechanisms of action, but they are all limited by their poor pharmacokinetic properties. Curcumin has a dual mechanism: it can not only directly inhibit DRP1 GTPase activity but also promote mitochondrial fusion through SIRT3-dependent OPA1 deacetylation, thereby achieving effective morphological remodeling in ALD and NAFLD models. However, its oral bioavailability is extremely low (approximately 1%), and its metabolism is rapid, which limits its application. In contrast, the mechanism of action of resveratrol is more indirect. It upregulates the expression of MFN2 through the AMPK/SIRT1/PGC-1α pathway. Its effect is slower, but the resulting network remodeling may be more lasting. Paeonol and its related metabolites act through transcription factor-driven programs, such as PKCε/STAT3-mediated MFN2 induction, trading a slower onset for more sustained effects. Alkaloids, such as matrine and astragaloside, adopt different strategies: matrine maintains fusion under Ca^2+^ stress by inhibiting the inner membrane protease OMA1, thus preventing the cleavage of fusion-competent L-OPA1, which is effective for Ca^2+^ imbalance-related injuries, such as renal ischemia–reperfusion injury. However, its therapeutic window is relatively narrow; for example, a crude dose of >10 g may lead to hepatotoxicity. Baicalein reverses oxidative stress-induced fragmentation by upregulating MFN1/2 and inhibiting DRP1 to reshape the transcriptome, but the existing evidence is still limited. These alkaloids may have certain chemical advantages, but they are also limited by toxicity risks and insufficient mechanistic evidence.

Flavonoid metabolites, such as morin, quercetin, baicalein, and sanggenon C, usually act on key signaling pathways and benefit from the regulation of the C_6_–C_3_–C_6_ skeleton. Morin inhibits NADPH oxidase 4 (NOX4) and upregulates MFN2 through the Epac1/Rap1 axis; quercetin indirectly inhibits mitochondrial fission by inducing lncRNA PVT1; baicalein destabilizes DRP1 and promotes mitochondrial fusion through the KLF4/MARCH5/DRP1 axis; sanggenon C, by blocking the ERK signaling pathway and thereby reducing the phosphorylation of DRP1 at Ser616, limits excessive fission and may exhibit cell-type-specific effects. Glycosylation, such as that in quercetin glycosides, can improve solubility and tissue distribution, but flavonoid metabolites usually have poor water solubility, and high concentrations may increase the risk of hemolysis, as shown by morin. Glycosides, such as salidroside, echinacoside, and ginsenoside Rg1, optimize mitochondrial dynamics through convergent but complementary mechanisms: salidroside directly inhibits DRP1 and enhances MFN2 expression, with good safety (LD50 > 5 g/kg); echinacoside can reduce mitochondrial fission, but its oral bioavailability is extremely low (F = 0.83%); ginsenoside Rg1 reduces mitochondrial fragmentation by promoting mitochondrial elongation and enhancing MFN2 expression. However, research on the mechanisms of glycosides remains relatively limited, and pharmacokinetic constraints are common.

Accordingly, combination strategies may be reasonable. For example, combining curcumin with alkaloids may enhance anti-fragmentation effects by inhibiting DRP1 activity and strengthening control over DRP1 turnover. Matrine may also be combined with Ca^2+^ homeostasis regulators, which may enhance therapeutic efficacy in specific contexts. In parallel, improving solubility and combining flavonoids with polyphenols or alkaloids may be a reasonable strategy to overcome pharmacokinetic bottlenecks while maintaining core therapeutic effects.

### 4.6 Quality control: induction of selective mitophagy

After outlining how natural products restore structural integrity through morphological regulation, this section turns to mitochondrial quality control, especially the selective removal of damaged mitochondria through mitophagy. This process is closely related to mitochondrial fission because mitochondrial fission is usually the initiating event for autophagic recognition and clearance. The therapeutic effects of natural product-mediated mitophagy regulation are deeply influenced by pharmacokinetic characteristics, especially the role of the gut microbiota in converting low-bioavailability natural product precursors into active metabolites, thereby forming a dynamic “gut microbiota–mitochondria axis,” which fundamentally shapes *in vivo* responses.

Mitophagy refers to the selective autophagic degradation of mitochondria and is a cornerstone of mitochondrial quality control. Its efficiency is closely related to the mitochondrial fission process described in Section 4.2 because fission can separate damaged mitochondrial regions for elimination. Natural products mainly enhance mitophagy through the PINK1/Parkin and AMPK/mTOR/ULK1 pathways and usually act at inter-organelle contact sites, such as MLCs (Wong et al., 2018). A key and often overlooked determinant of their *in vivo* efficacy is pharmacokinetics. Gut microbiota-mediated conversion of parent metabolites into active metabolites may help resolve the common bioavailability paradox.

#### 4.6.1 Precision intervention targeting core autophagy pathways

This subsection addresses the quality control dimension of our four-dimensional framework: selective mitophagy relies on the balanced mitochondrial morphology outlined in Section 4.5, and works synergistically with the biogenesis dimension to form a closed-loop mitochondrial renewal cycle. Mitochondrial quality control depends not only on structural remodeling but also on autophagy-mediated removal of damaged metabolites. Increasing evidence indicates that natural products enhance mitochondrial autophagy by targeting core pathways, especially the classical PINK1/Parkin-mediated mitochondrial autophagy pathway, one of the most widely studied mammalian mitophagy pathways. Dysfunction of this pathway is closely related to familial Parkinson disease, highlighting its core role in maintaining neuronal homeostasis. Mechanistically, natural products can act on multiple regulatory nodes, including stabilizing and activating PINK1 on the mitochondrial outer membrane, recruiting Parkin and activating its E3 ubiquitin ligase activity, promoting substrate ubiquitination, such as that of MFN1/2 and the voltage-dependent anion channel (VDAC), and promoting the association between mitochondrial autophagy receptors and autophagosomes, thus accelerating the removal of damaged mitochondria. For example, ginsenoside 20(S)-Rh2 can activate the PINK1/Parkin axis, promote Parkin recruitment to damaged mitochondria, and enhance the ubiquitination of mitochondrial outer membrane substrates, such as mitofusin 1/2 (MFN1/2) and VDAC, thereby marking these organelles for degradation. This clearance function complements the regenerative activity of its related metabolite ginsenoside Rg1, thus establishing a functional coupling between mitochondrial clearance and mitochondrial biogenesis.

Artemisinin is a sesquiterpene lactone with an internal endoperoxide bridge. It is derived from *Artemisia annua* L*.* and is famous for its antimalarial activity. It has also been studied as a potential anticancer drug, partly because it can induce mitochondrial depolarization, increase membrane rigidity, and increase intracellular ROS levels. This stress pattern is consistent with the activation of PINK1/Parkin-dependent mitophagy, which may occur by promoting PINK1 accumulation and downstream Parkin activation (Zhu et al., 2022), thus highlighting the therapeutically targetable stress–mitophagy axis in cancer treatment. In leukemia models, the IC_50_ range of artemisinin is 1–10 μM, and it can induce mitophagy in tumor cells. While this concentration range is tumor-microenvironment-specific and leverages iron-dependent activation, partially circumventing the Core Paradox (Introduction), systemic exposure in non-malignant tissues remains a concern. The effect of artemisinin is highly iron-dependent because its endoperoxide bridge requires Fe^2+^-mediated cleavage to generate ROS (Mancuso et al., 2020). In iron-deficient tissues, such as some neurodegenerative lesions, its ability to induce mitophagy may be significantly reduced. However, few studies have explored this limitation, leaving an important translational gap across different disease microenvironments. This highlights the crucial role of the disease microenvironment in determining the therapeutic effects of natural products.

#### 4.6.2 Bidirectional regulation strategy of autophagy pathways

Natural products can also provide context-sensitive bidirectional regulation, which usually reflects the principle of mitohormesis. Flavonoids have fine regulatory effects on BNIP3L/NIX-mediated mitophagy: in neuroinflammatory models, luteolin, a flavonoid metabolite derived from multiple sources, including *Lonicera japonica* Thunb. (family Caprifoliaceae) and *Perilla frutescens* (family Lamiaceae), inhibits excessive mitophagy by suppressing BNIP3L/NIX function (Ma et al., 2024), which is consistent with neuroprotection. In glutamate-induced neuroinflammatory models, luteolin acts in the concentration range of 10–20 μM, blocking excessive autophagy to protect neurons (Vongthip et al., 2024). This reflects context-dependent fine-tuning: when clearance is beneficial, autophagy is enhanced, whereas when overactivation is harmful, it is suppressed. Quercetin amplifies autophagic flux through the SIRT1/PINK1/Parkin cascade; in neuroprotection and anti-aging studies, it upregulates the expression and/or activity of PINK1 and Parkin through SIRT1-dependent signaling. In neuroprotective models, quercetin acts in the concentration range of 5–20 μM, activating the SIRT1-dependent signaling pathway and upregulating PINK1 and Parkin expression and/or activity (Vafadar et al., 2020). However, the direct activation of SIRT1 by quercetin, similar to that of resveratrol, may be subject to methodological controversy related to fluorescent substrate assays. Its effect may be more indirect, perhaps through upstream kinases or regulation of NAD^+^ levels.

These effects may complement those of berberine, an isoquinoline alkaloid derived from *Berberis* species and other medicinal plants, which strongly affects the AMPK pathway and thereby promotes mitophagy. As a cellular energy sensor, AMPK is activated under ATP depletion or energy stress. It promotes autophagy by directly phosphorylating and activating ULK1 while inhibiting its upstream inhibitory regulator mTORC1. Berberine triggers mitophagy at 25–35 μM to eliminate damaged mitochondria within the quality control regulatory axis. Its AMPK-activating mechanism varies across cell types: complex I inhibition dominates hepatocytes, while LKB1-mediated upstream phosphorylation prevails in cardiomyocytes, with no unified validated pathway to date (Feng et al., 2021). In addition, high concentrations of berberine may induce mitochondrial oxidative stress (Rad et al., 2017), thereby offsetting the benefits of mitophagy by damaging healthy mitochondria. This highlights that there may be toxicity thresholds beyond the optimal range of mitochondrial hormetic effects. At present, the concentration range that can balance beneficial AMPK activation and harmful ROS induction has not been determined, which brings uncertainty to dose optimization. This mechanism supports berberine’s potential [LTR30.1] in the treatment of insulin resistance, type 2 diabetes (Xie et al., 2023), non-alcoholic fatty liver disease, and certain cancers (Zhao et al., 2019).

Dihydrochalcone (DDC) is a dihydrochalcone metabolite isolated from a specific plant source, which further supports the synergistic relationship between mitochondrial quality control and the fate of breast cancer cells. DDC can significantly increase the LC3-II level in MCF-7 and MDA-MB-231 cells, for example, by about 7.7 times in MCF-7 cells, inhibit the mTOR signaling pathway by reducing Ser2448 phosphorylation, and upregulate Beclin-1 to promote autophagosome formation and organelle removal (Liu et al., 2023). In breast cancer cell models involving mitochondrial autophagy and intrinsic apoptosis, DDC acts in the concentration range of 52.5–66.4 μM: on the one hand, it activates autophagy to remove damaged organelles; on the other hand, it disrupts the mitochondrial membrane potential (ΔΨm) to trigger the intrinsic apoptosis pathway. For example, ΔΨm is reduced by approximately 70% in MDA-MB-231 cells, with increased levels of apoptotic proteins such as BAX and BIM and reduced levels of anti-apoptotic factors such as BCL2 and MCL1 (Xiao et al., 2017). Importantly, DDC can also regulate excessive mitochondrial fission by reducing DRP1 phosphorylation and upregulating fusion proteins, including MFN1, MFN2, and OPA1, thereby maintaining mitochondrial dynamic equilibrium (Mendez-Callejas et al., 2024). This makes DDC a dual-pathway regulator that can induce autophagy and apoptosis while rebalancing mitochondrial morphology. This multidimensional regulatory strategy may be particularly relevant for anticancer effects.

#### 4.6.3 Dynamic balance regulation of autophagy-biogenesis

The interaction between mitochondrial biogenesis and clearance is crucial for maintaining cellular homeostasis. A variety of natural products maintain this balance by coordinating autophagy and biogenesis. Thiamine is widely found in the human diet. It has been reported to regulate mitophagy through the PINK1/Parkin axis and the hypoxia-responsive HIF-1α/BNIP3 pathway. Because HIF-1α coordinates cellular adaptation to hypoxia and transcriptionally upregulates BNIP3, thereby initiating BNIP3-mediated mitophagy, maintaining appropriate HIF-1α/BNIP3 signaling is crucial for indirect regulation of mitochondrial clearance. In a dietary supplementation study in grass carp under hypoxic conditions, thiamine was administered at 0.81–1.32 mg/kg. Within this dose range, thiamine maintained normal HIF-1α/BNIP3 axis function, regulated BNIP3 expression, and activated BNIP3-mediated mitophagy under hypoxic conditions, thereby reducing mitochondrial damage (Huang et al., 2024). However, the current evidence mainly comes from thiamine-deficiency models and indirect inference. The direct molecular targets of thiamine and its pathway interactions remain to be verified, highlighting a major gap in the evidence chain.

Rhein activates mitochondrial biogenesis through the SIRT1/PGC-1α pathway and dynamically regulates autophagy-related proteins, thus forming a closed-loop mechanism of “biogenesis–repair–clearance” (Yin et al., 2022). Curcumin and berberine may have similar system-level regulatory characteristics. The mechanism of action of curcumin deserves special attention: it has been reported that curcumin can activate transcription factor EB (TFEB), a key regulator of lysosomal biogenesis, and enhance the degradation ability of lysosomes by upregulating lysosomal proteins and autophagy-related genes (Ruan et al., 2022). This enhanced lysosomal function is crucial for efficient mitochondrial autophagy and may promote mitochondrial clearance through MLCs. However, curcumin-mediated TFEB activation appears to be tissue-specific: in hepatocytes, it involves the inhibition of mTORC1, whereas in neurons, it may involve direct phosphorylation-dependent regulation (Ruan et al., 2022). The potential mechanism of this tissue specificity remains unclear, which makes it more difficult to predict its efficacy in different diseases and represents a key direction for future research. Resveratrol and isochlorogenic acid A (ICGA) can also enhance PINK1/Parkin-dependent mitophagy, although their mechanisms of action are different: resveratrol promotes the removal of Aβ-damaged mitochondria in Alzheimer disease (Meza-Torres et al., 2020; Ren et al., 2017), and ICGA reduces lipid-driven inflammation in non-alcoholic fatty liver disease through NRF2-mediated LC3-II upregulation, which reflects their association with different pathological pathways (Lai et al., 2024).

The activation of these natural product-induced quality control pathways highlights the core role of cellular energy status in mitochondrial monitoring. However, the interaction between SIRT1 and AMPK in natural product-mediated mitophagy has not been fully clarified. In some cases, they work together; for example, resveratrol activates both pathways to enhance autophagy. In other cases, they may be antagonistic; for example, high-dose berberine activates AMPK but may inhibit SIRT1 by consuming NAD^+^. It remains unclear how natural products adjust this balance to promote beneficial mitophagy, and this uncertainty is a major obstacle to optimizing their multi-target therapeutic potential.

#### 4.6.4 Gut microbiota–mitochondria axis

The regulation of mitochondrial autophagy is not limited to intracellular mechanisms but also involves host–microbiota interactions (Figure 6). As an important biotransformation hub, the gut microbiota helps to explain the bioavailability paradox of natural products. Microbial communities can convert parent metabolites into active metabolites with higher bioavailability. After these metabolites are absorbed, they can regulate mitochondrial autophagy and broader aspects of mitochondrial physiology, thus forming a gut microbiota–mitochondria axis (Park et al., 2021; Xing et al., 2024; Wang et al., 2025; Kern et al., 2025).

**FIGURE 6:**
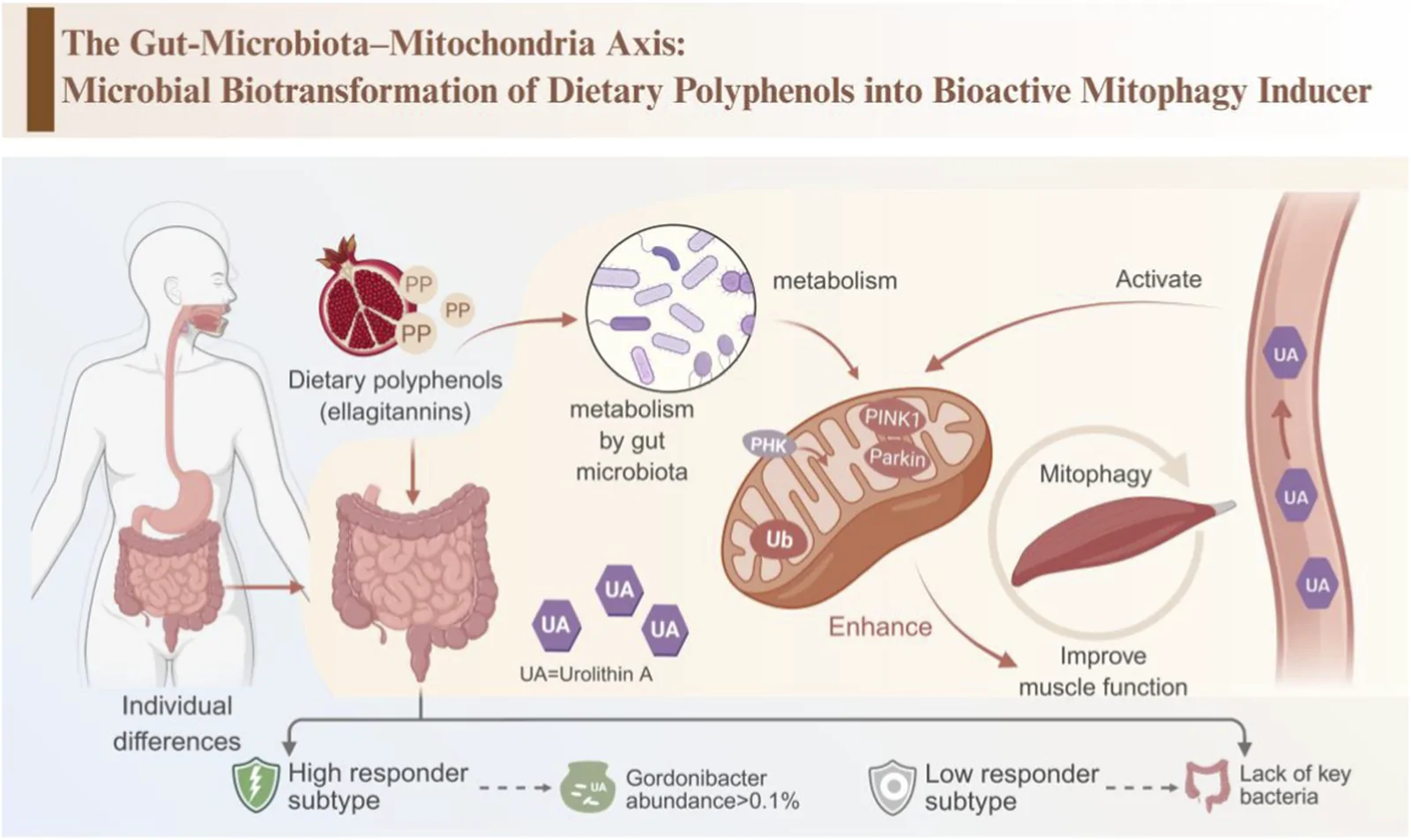
The gut-mitochondria connection: how microbial biotransformation of dietary polyphenols creates bioactive mitophagy inducers. This schematic illustrates the gut microbiota–mitochondria axis using urolithin A (UA) as an example. Dietary polyphenols (ellagitannins) from sources such as pomegranate (PP) are metabolized by gut microbiota into UA. Production efficiency varies between individuals: the high-responder subtype is characterized by *Gordonibacter* abundance >0.1%, while the low-responder subtype lacks key bacteria. UA activates the PINK1/Parkin pathway, leading to ubiquitination (Ub) and enhanced mitophagy, which promotes muscle function. The diagram depicts the metabolic conversion in the gut, the systemic circulation of UA, and its action on mitochondrial quality control.

Mechanistically, pomegranate and berry ellagitannins are metabolized by colonic *Gordonibacter* to UA, a lipophilic, stable metabolite that crosses the intestinal barrier and targets cardiac, skeletal muscle, and neuronal mitochondria (Li R. et al., 2023). UA improves overall mitochondrial function, and clinical trials show that 500–1,000 mg UA increases mitophagy activity and restores muscle mitochondrial performance (Ryu et al., 2016; Ju et al., 2025). Importantly, UA production is not uniform but shows significant interindividual differences, which are determined by the composition of each person’s gut microbiota, that is, the “metabotype.” This means that in clinical trials, subjects enriched in specific metabotypes (for example, metabotype A) and *Gordonibacter* may produce and absorb large amounts of UA, whereas subjects with different microbial spectra (such as metabotypes B or C) may produce very little or no UA. This poses a major challenge to clinical translation, requiring personalized strategies, such as metabolic screening or direct UA supplementation.

Clinical data further support this view: UA (Mitopure®) has been shown to be safe in humans, enhance respiratory chain function, and improve muscle strength and endurance in older adults (Ryu et al., 2016; Singh et al., 2022; Wang Y. H. et al., 2023). The research paradigm of UA strongly indicates that the gut microbiota not only helps solve the pharmacokinetic problem of low bioavailability of parent metabolites but is also a major source of individual differences and may become a key confounding factor affecting pharmacological outcomes. Therefore, for natural products that are susceptible to microbial metabolism, such as ellagitannins and some flavonoids, not considering metabotype stratification in clinical trials is effectively equivalent to testing different interventions in different subgroups. In practice, subjects may be exposed to different active metabolites under completely different exposure conditions. This heterogeneity will dilute the apparent therapeutic effect and may lead to incorrect conclusions about the therapeutic effect of natural products, including false-negative results. The gut microbiota–mitochondria axis helps to solve the contradictions in the bioavailability of natural products and shifts the focus of translational research from parent metabolites to microbial-derived metabolites, thereby enabling more accurate interventions, such as personalized dietary supplementation and probiotic-based combined treatment strategies (Singh et al., 2022). Therefore, in order to obtain meaningful and interpretable clinical data, intestinal microbiome analysis and subsequent metabotype stratification should be included in the research design. Alternatively, directly supplementing identified active microbial metabolites, such as Mitopure®, can bypass this variability and represent a more standardized treatment strategy. The UA research paradigm complements earlier mechanisms, such as Parkin recruitment driven by ginseng saponin 20(S)-Rh2 and PINK1 accumulation triggered by artemisinin. At the same time, it emphasizes that microbiome-dependent biotransformation is not only related to mitophagy but also to mitochondrial biogenesis (Section 4.1) and mitochondrial dynamics (Section 4.5), which helps to maintain mitochondrial homeostasis. In terms of clinical translation, the success of Mitopure® highlights the feasibility of “natural product–microbiome axis” therapy; however, individual differences in metabolic type and exposure are still the core challenges of pharmacokinetics (Section 5.1.2).

#### 4.6.5 Pathway preference and translation bottleneck comparison of mitophagy inducers

Natural products can induce mitophagy through several pathways, including the canonical PINK1/Parkin cascade, the AMPK/mTORC1/ULK1 axis, and the BNIP3/BNIP3L (NIX) pathway. However, there are significant differences in the selectivity and translational feasibility of these pathways. Among PINK1/Parkin activators, ginsenoside 20(S)-Rh2 can promote Parkin recruitment and substrate ubiquitination, thus removing mitochondria containing toxic α-synuclein in Parkinson disease models; however, as a saponin, its absorption and overall pharmacokinetic characteristics still need to be carefully evaluated. Artemisinin itself has limited capacity to trigger mitochondrial-specific clearance. Its derivative artesunate induces PINK1 accumulation through iron-dependent ROS generation, which provides a theoretical basis for targeting the iron-rich tumor microenvironment, but it may pose a risk of excessive stress in normal tissues when used at high doses.

In contrast, urolithin A is a gut microbiota-derived metabolite. It is a potent PINK1/Parkin activator that can overcome the bottleneck of low oral bioavailability associated with many phenolic metabolites. Its main limitation lies in the differences in metabolic phenotypes between individuals, which lead to heterogeneous therapeutic responses and require individualized treatment strategies, thus becoming a major obstacle to its broader clinical application.

Regarding the AMPK/mTORC1/ULK1 axis, cypressine is a potent AMPK activator, which can relieve the inhibition of ULK1 and initiate autophagy by inhibiting mTORC1, thus exerting its advantage in mitochondrial clearance in metabolic diseases and cancer. Its advantage is the efficient activation of AMPK; its risks include dose-dependent toxicity, including potential cardiotoxicity, and inhibition of mitochondrial complex I, which may aggravate energy metabolic dysfunction under susceptible conditions. Quercetin enhances autophagy through the SIRT1/PINK1 pathway and acts on more upstream links, usually producing a milder and more sustained effect. However, its extremely low oral bioavailability, approximately 1–5%, and limited BBB penetration limit its application in the central nervous system. For the BNIP3/BNIP3L (NIX) pathway, luteolin can inhibit excessive BNIP3L/NIX-mediated mitophagy in the neuroinflammatory environment, thus preventing excessive removal of neurons. Despite the broad prospects of receptor-level specificity, evidence remains insufficient regarding its effective removal of damaged mitochondria in nervous tissue, and bioavailability challenges also persist.

A variety of natural products coordinate mitophagy through complementary regulatory mechanisms. Rhein combines SIRT1/PGC-1α-driven biogenesis with dynamic regulation of autophagy proteins, thereby constructing a closed-loop model of biogenesis–repair–clearance. Curcumin can activate TFEB, a key regulator of lysosomal biogenesis and coordinated expression of lysosomal and autophagy-related genes, thereby enhancing lysosomal function and overall autophagic flux. In terms of pathway preference, the PINK1/Parkin axis remains the main pathway targeted by natural products to mediate mitophagy, whereas AMPK activation, for example by berberine or more indirectly by resveratrol, initiates autophagy under energy stress (Wang B. et al., 2023). There are relatively few examples of direct regulation of the BNIP3/NIX pathway, and luteolin is an example of inhibitory regulation. In terms of efficacy and specificity, artemisinin, with iron-dependent activity, and luteolin, with receptor-level regulation, show relatively high specificity; the therapeutic effect of berberine is substantial but accompanied by an obvious risk of off-target toxicity; urolithin A has significant *in vivo* therapeutic effects, but responses vary markedly among individuals.

Translational applications face many bottlenecks. Many parent metabolites, such as quercetin and saponins, have low oral bioavailability. Other metabolites, such as berberine and high-dose artemisinin, have a narrow therapeutic window, while UA is limited by metabolic heterogeneity. Reasonable combination therapy may help alleviate these limitations: if the dose of berberine can be strictly controlled and combined with PINK1/Parkin activators, such as 20(S)-Rh2, or low doses of artemisinin, it may improve autophagic flux by activating upstream autophagy and enhancing downstream selective mitochondrial clearance. Similarly, combining UA with probiotics or prebiotics may optimize microbial biotransformation and partially alleviate the restrictions associated with metabotypes. Overall, natural products can effectively remove dysfunctional mitochondria, but their removal efficiency is significantly affected by their chemical skeleton and pharmacokinetic characteristics. Microbiome-derived metabolites, such as urolithin A, illustrate how pharmacokinetic adaptability can overcome inherent limitations (see Section 5). However, effective mitochondrial quality control also depends on the core antioxidant defense mechanism, especially the regulation of organelle-derived ROS homeostasis.

### 4.7 Defense mechanisms: fortifying antioxidant systems and regulating apoptosis

This subsection illustrates the final oxidative defense dimension of our four-dimensional framework, which works in tandem with biogenesis, morphological balance, and mitophagy to counteract oxidative damage and lock in stable mitochondrial function. Oxidative defense can protect the existing mitochondrial network from damage and regulate the cell death threshold (Figure 7). Oxidative stress is a common cause of mitochondrial dynamics imbalance. However, the comprehensive “defense” strategy is not limited to removing ROS but also includes regulating mitochondrial apoptosis through MOMP mediated by the Bcl-2 family (Delbridge et al., 2017). The Bcl-2 protein family acts as a molecular rheostat that regulates MOMP, and the occurrence of MOMP marks an irreversible step toward cell death. The family contains three functional subgroups: (1) anti-apoptotic proteins, such as Bcl-2, Bcl-xL, and Mcl-1, which maintain mitochondrial integrity by sequestering pro-apoptotic factors; (2) pro-apoptotic effector proteins, such as Bax and Bak, which undergo oligomerization after activation and form MOMP pores; (3) BH3-only proteins, such as Bim, Bid, and Puma, which translate cellular stress signals and trigger apoptosis by neutralizing anti-apoptotic members or directly activating Bax/Bak (Czabotar et al., 2013). The dynamic balance between these subfamilies determines cell fate by modulating the MOMP threshold, which represents the “point of no return” in apoptosis (Delbridge et al., 2017). Nanoparticles regulate this network in a bidirectional and environment-dependent manner: in neurodegenerative diseases, they can increase the MOMP threshold by enhancing Bcl-2/Bcl-xL expression or inhibiting Bax activation; in cancer, they can inhibit Mcl-1 or directly activate BH3-only proteins, thereby lowering the threshold and linking antioxidant defense with precise apoptosis regulation (Perciavalle et al., 2012).

**FIGURE 7:**
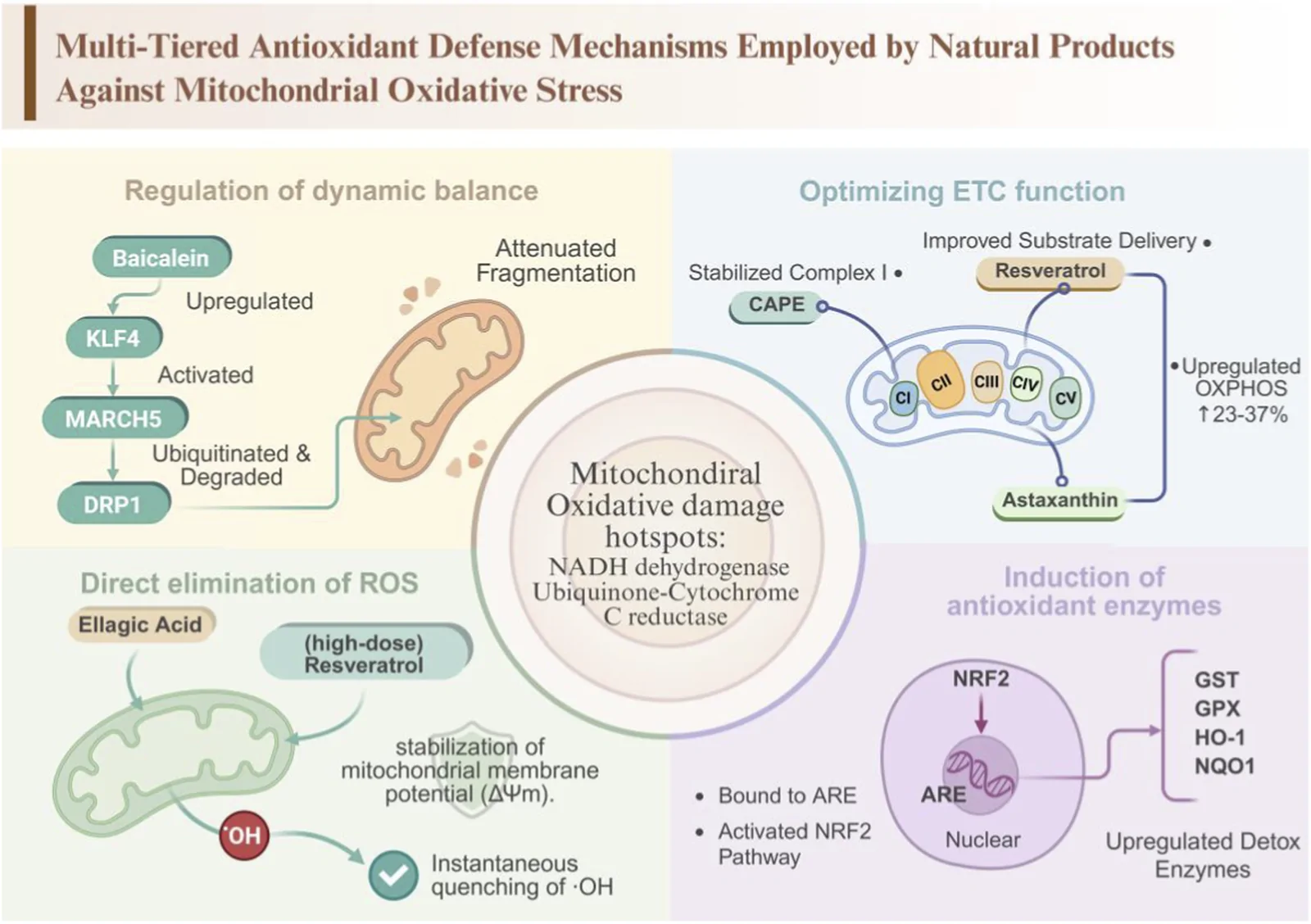
A layered shield: the multi-tiered antioxidant defense mechanisms of natural products against mitochondrial oxidative stress. The figure illustrates a layered antioxidant defense system against mitochondrial oxidative damage. The system contains four main strategies: (1) regulation of dynamic balance through the KLF4-MARCH5 axis (such as baicalein) to reduce mitochondrial fragmentation; (2) direct ROS scavenging by tannic acid and high-dose resveratrol to neutralize hydroxyl radicals and stabilize mitochondrial membrane potential; (3) optimization of electron transport chain function by stabilizing complex I (such as caffeic acid phenethyl ester (CAPE)) and improving substrate delivery (such as resveratrol and astaxanthin), thereby upregulating oxidative phosphorylation metabolites and improving oxidative phosphorylation efficiency (23–37%); and (4) induction of antioxidant enzymes through the Nrf2 pathway, thereby increasing the expression of detoxification enzymes (GST, GPX, HO-1, and NQO1). Arrows indicate the interactions among these coordinated defense mechanisms.

The core of mitochondrial oxidative stress lies in the imbalance between excessive production of ROS and insufficient endogenous antioxidant capacity. The ETC is the main source of ROS: complex I (NADH: ubiquinone oxidoreductase) and complex III (ubiquinol–cytochrome *c* oxidoreductase) produce superoxide anions (O_2_^−^) through electron leakage during electron transfer; reverse electron transport (RET), especially when the mitochondrial membrane potential (ΔΨm) increases, can further enhance the production of ROS (Ryu et al., 2024; Chan, 2012). In addition, ROS can damage enzyme activity, disrupt calcium homeostasis, and aggravate cellular damage (Marx et al., 2024; Tapias, 2019). The MERC microdomain precisely regulates bidirectional calcium signaling, thereby affecting both ROS production and susceptibility to apoptosis. Calcium released from the endoplasmic reticulum through inositol 1,4,5-triphosphate receptors (IP3Rs) enters the mitochondrial matrix through voltage-dependent anion channel 1 (VDAC1). This process is usually facilitated by tethering proteins at the MERC interface. Ca^2+^ activates the pyruvate dehydrogenase complex, thereby supporting ATP production. However, this process is subject to strict spatial restrictions: artificial tethering experiments show that when the membrane spacing is less than 10 nm, overcrowding will hinder the opening of IP3R channels and reduce the efficiency of calcium ion transport (Prudent and McBride, 2017; Rowland and Voeltz, 2012). Excessive or continuous Ca^2+^ transfer at MERCs may lead to mitochondrial Ca^2+^ overload, stimulate ROS generation, and increase susceptibility to apoptosis. A variety of natural products fight such damage through multiple mechanisms to maintain the structure and function of mitochondria, including the regulation of Bcl-2 family proteins and the regulation of calcium ion handling at MERCs.

#### 4.7.1 Bidirectional regulation of core antioxidant enzymes and energy metabolism

Polyphenolic metabolites, such as caffeic acid phenethyl ester (CAPE), tannic acid, and ferulic acid, play a role through two core mechanisms: antioxidant enzyme networks and mitochondrial energy metabolism, thus providing systemic protection, although their respective mechanisms of action are different. CAPE is found in propolis, wine, and some plant sources. It improves the efficiency of OXPHOS through complex I-related mechanisms, thereby increasing ATP yield in target tissues and inhibiting ROS generation at major sites of oxidative production. In cardiotoxicity models, CAPE acts in the concentration range of 10–30 μmol/kg, enhancing complex I function and increasing SOD/CAT activity to reduce ROS leakage (Ali et al., 2020; Akyol et al., 2014).

The exact structural basis of the interaction between CAPE and complex I has not been determined, and targeted biochemical and structural studies are still needed. At the same time, CAPE can enhance the activity of key antioxidant enzymes, including SOD and CAT, making it a reasonable intervention strategy for ischemia–reperfusion injury and neurodegenerative diseases (Zhao et al., 2025; Zhang et al., 2014). Tannic acid supplements this mechanism by stabilizing mitochondrial membrane potential (ΔΨm) and ATP levels and reversing mitochondrial swelling in metabolic syndrome-related kidney injury models. In cell models, tannic acid is effective in the concentration range of 1.5–3.0 μg/mL. It can remove hydroxyl radicals (·OH), thus stabilizing membrane potential and reversing mitochondrial swelling. Its effect is consistent with the Nrf2/NF-κB regulatory activity of tannic acid, which rebalances ROS homeostasis by regulating inflammation, thus expanding the multi-target application of polyphenolic metabolites in chronic inflammatory diseases. In the macrophage model, ferulic acid (a phenolic acid metabolite derived from *Oryza sativa* L. and *Angelica sinensis* Oliv. Diels.) showed concentration-dependent anti-inflammatory effects in the concentration range of 10–100 μM, inhibiting the production of nitric oxide induced by LPS at therapeutically relevant concentrations, and did not show cytotoxicity (Gong et al., 2022; Liu et al., 2021).

In cancer and neuroprotection models, EGCG can effectively induce phase II detoxification enzymes in the concentration range of 5–50 μM, thus establishing a rapid antioxidant defense system. In contrast, resveratrol focuses on the root cause of bioenergetic dysfunction: it can effectively improve the delivery of reducing equivalents to the ETC (Meza Torres et al., 2020) and activate SIRT1-dependent deacetylation (Xing et al., 2024). This dual effect reshapes the NAD^+^ metabolic network and enhances resistance to oxidative stress, partly because it improves the assembly quality of respiratory chain supercomplexes (SCs) (Xing et al., 2024). Other emerging polyphenolic metabolites have further expanded this treatment route. For example, *Gastrodia elata* can relieve metabolic-associated fatty liver disease through the lipid peroxidation–mitophagy axis (Chen Y. X. et al., 2024), while isochlorogenic acid A can penetrate the BBB and exert synaptoprotective effects in neurodegenerative diseases (Lai et al., 2024). The progression from basic antioxidant mechanisms to tissue-specific network regulation highlights the multidimensional value of polyphenols in bioenergetic reprogramming and maintaining redox homeostasis.

#### 4.7.2 Membrane system repair and dynamic balance regulation

Flavonoids reduce mitochondrial oxidative stress through two major mechanisms. First, they inhibit NOX4 through the Epac1/Rap1 signaling axis, thereby reducing ROS production at the source. In myocardial ischemia–reperfusion and hypoxia–reoxygenation models, vitexin inhibits NOX4 expression through the Epac1/Rap1 signaling pathway at a concentration of 10 μM and stabilizes the electron transfer efficiency of complex III, thereby reducing ROS production at the source and maintaining oxidative phosphorylation function (Duan et al., 2024). Second, by supporting the electron transfer efficiency of complex III, flavonoids maintain oxidative phosphorylation, improve ATP synthesis, and alleviate the energy crisis in ischemia–reperfusion models. Apigenin, which shares the C_6_–C_3_–C_6_ flavonoid scaffold, further extends this function: in addition to directly scavenging ROS, it also inhibits mitochondrial apoptotic signaling through the BAX/BCL-2 axis (Chen Y. et al., 2024), thereby regulating the MOMP threshold and producing synergistic antioxidant and anti-apoptotic protection. In neuroprotection models, apigenin is administered intraperitoneally at a dose of 50 mg/kg, where it scavenges free radicals, increases the Bcl-2/Bax ratio, and inhibits mitochondria-dependent apoptosis (Yang et al., 2021). This is consistent with the role of the Bcl-2 family as a central regulator of MOMP: Bcl-2, an anti-apoptotic protein, sequesters pro-apoptotic BAX, thereby preventing pore formation in the outer mitochondrial membrane.

Baicalein and baicalin together form a complementary regulatory system. Baicalein regulates mitochondrial fission–fusion balance through the KLF4/MARCH5/DRP1 axis, improving membrane potential recovery efficiency by about 1.8-fold. In liver injury models, administration of 50 mg/kg baicalein reduces mitochondrial fragmentation and improves membrane potential recovery (de Oliveira et al., 2015; Wu et al., 2021; Li R. et al., 2023). Baicalin, in turn, links the mitochondrial ROS (mtROS) threshold with ATP abundance, establishing a feedback mechanism that regulates organellar responses to oxidative load (Hu et al., 2022; Zhao et al., 2022). The connection between membrane potential recovery and energy sensing provides a real-time control paradigm for mitochondrial quality assurance, although its translational potential remains limited because its mechanism has not yet been fully characterized. From the molecular level to the ultrastructural level, flavonoids reduce superoxide levels by activating SOD2 and enhance OPA1-mediated cristae remodeling, thereby improving cristae density. In cell models, luteolin activates SOD2 and enhances OPA1-mediated cristae remodeling in the concentration range of 10–20 μM, thereby reducing peroxide levels and increasing cristae density (Ma et al., 2024). Combined with the dynamic regulation of ΔΨm by baicalin, these hierarchical effects form a dual safeguard for mitochondrial structural and functional integrity.

#### 4.7.3 Others: oxidative phosphorylation restoration and ROS dynamic balance mechanisms

In addition to flavonoids, other natural products also protect mitochondria through unique mechanisms, such as transcriptional reprogramming and mitohormetic regulation. Astaxanthin (a carotenoid metabolite derived from the microalga *Haematococcus pluvialis* Flotow, as well as from aquatic organisms such as shrimp and crabs) is a fat-soluble carotenoid that enhances OXPHOS by upregulating OXPHOS-related proteins, including complex IV subunits COX4I1/COX4I2, the complex III core metabolite UQCRC2, the complex I subunit NDUFA9, and TFAM, thus improving the efficiency of cellular oxidative phosphorylation. In neuronal cell injury models, a concentration of 80 μg/L increases ATP synthesis by 23–37% (Yan et al., 2024). By regulating key factors such as SOD1, SOD2, PARKIN, PINK1, and MFN1, astaxanthin can reduce oxidative stress and mitochondrial dynamics disorders in equine metabolic syndrome (EMS) models, thereby improving systemic metabolism (Mularczyk et al., 2022). It remains unclear how this broad transcriptional process is coordinated; upstream master transcription factors such as PGC-1α and NRF2 may be involved.

In contrast, the water-soluble isothiocyanate sulforaphane (SFN), abundant in cruciferous vegetables (the sprouts or flower heads of *Brassica oleracea* L. var. italica), reprograms mitochondrial function at the transcriptional level through the Nrf2/ARE pathway. SFN induces phase II enzymes (HO-1 and NQO1) and, through a PGC-1α-dependent mechanism, improves maximal respiration, spare respiratory capacity, basal respiration, and ATP production, while reducing proton leak. In the placental model, SFN plays a role in the concentration range of 5–50 μM, activates the NRF2/ARE pathway to induce HO-1/NQO1 expression, and optimizes the respiratory chain in a PGC-1α-dependent manner, thus improving the maximum respiratory rate and spare respiratory capacity, basal respiration, and ATP generation, while reducing proton leakage. It can also improve the mRNA levels of genes encoding mitochondrial respiratory-chain complexes and correct the increased expression of fission genes induced by hypoxia and oxidative stress. Functionally, SFN can prevent hypoxia-driven mitochondrial membrane damage, offset hypoxia/superoxide-induced dysfunction in syncytiotrophoblast cells *in vitro*, and improve mitochondrial respiration in placental trophoblasts from preeclamptic pregnancies (Langston-Cox et al., 2020).

Some natural products indirectly enhance mitochondrial fitness and improve redox stability by regulating ROS. Arecoline (a pyrrolidine alkaloid metabolite extracted from the seeds of *Areca catechu* L.) embodies this dose-dependent logic: at low ROS levels, it promotes autophagic flux to eliminate damaged mitochondria; under excessive oxidative stress, it helps stabilize intracellular redox balance and limit oxidative injury through a mechanism involving SIRT3. SIRT3, a mitochondrial NAD^+^-dependent deacetylase, is a key enzyme regulating ROS metabolism (such as via SOD2 and IDH2), fatty acid and amino acid catabolism, and the respiratory chain (Wang S. et al., 2023). How arecoline precisely acts on or regulates SIRT3 to achieve “ROS homeostatic regulation” warrants further investigation. Conceptually, natural product-mediated regulation of mitochondrial redox is not merely a matter of scavenging or inhibition but rather the dynamic maintenance of adaptive, state-dependent redox balance.

#### 4.7.4 Hierarchy and efficiency comparison of antioxidant defense strategies

The antioxidant defense network orchestrated by natural products operates at multiple levels: it directly scavenges free radicals, induces enzymes, repairs membranes, and regulates mitochondrial dynamics. It shows distinct emphases across different chemical classes. In the field of direct ROS neutralization, polyphenols dominate: tannic acid rapidly quenches the hydroxyl radical (·OH) through its polyphenolic hydroxyl groups, while also helping to stabilize ΔΨm (Gong et al., 2022; Liu et al., 2021). However, at higher concentrations, it may increase membrane permeability and thereby narrow the therapeutic window. Resveratrol can also scavenge free radicals, although its mechanism of action is less direct than that of tannic acid (Martínez-Abundis et al., 2016). Therefore, direct scavenging is rapid but relatively short-lived, and supra-physiological doses may cause membrane-related adverse effects.

In contrast, metabolites that induce antioxidant enzymes can establish more durable defenses. EGCG strongly induces phase II enzymes (GST, GPX, and HO-1), which is consistent with its roles in cancer prevention and neuroprotection, although higher doses are often required because of limited oral bioavailability (Andreu Fernández et al., 2020). SFN is a canonical Nrf2 activator that increases HO-1 and NQO1 with reliable efficacy and good aqueous solubility (Langston-Cox et al., 2020). Astaxanthin not only upregulates SOD1/2 but also maintains respiratory chain integrity, thereby increasing OXPHOS efficiency by 23–37%; its lipophilicity enables enrichment in cellular membranes, thereby playing a dual role in enzyme induction and structural repair (Yan et al., 2024).

From a more fundamental level, improving the performance of the ETC can reduce the production of ROS at the source: CAPE can enhance the function of complex I, inhibit electron leakage, and improve SOD/CAT activity; these characteristics are particularly important in ischemia–reperfusion injury. Resveratrol can promote the transfer of reducing equivalents such as NADH to the ETC, thus improving bioenergetic efficiency and reducing ROS production (Ali et al., 2020; Meza-Torres et al., 2020). These strategies can usually provide more lasting protection.

Regulating mitochondrial dynamics can also indirectly enhance antioxidant capacity. Baicalein inhibits mitochondrial fission through the KLF4/MARCH5/DRP1 axis, restoring the mitochondrial membrane potential by about 1.8-fold (de Oliveira et al., 2015). Baicalin and luteolin further stabilize the system by establishing mtROS-ATP feedback control and enhancing OPA1-mediated cristae remodeling, respectively. These interventions improve the resilience of redox homeostasis by enhancing structural stability (Hu et al., 2022; Ma et al., 2024). Although these effects are indirect, they may be persistent and can complement direct ROS scavenging and enzyme induction. Arecoline is a typical example of mitohormetic regulation: low levels of ROS signaling can trigger protective autophagy, and under high oxidative load, the SIRT3-dependent mechanism can limit ROS levels to the physiological range, reflecting the integration of energy sensing and redox regulation (Wang S. et al., 2023). Importantly, natural products can also regulate the Bcl-2 family network in both directions, thus fine-tuning the sensitivity of mitochondrial apoptosis. In neurodegenerative diseases, natural products may increase the MOMP threshold by enhancing Bcl-2/Bcl-xL expression or inhibiting Bax activation; in cancer, natural products may reduce this threshold by inhibiting Mcl-1 or activating BH3-only proteins, thus linking antioxidant defense with apoptotic regulation (Perciavalle et al., 2012; Delbridge et al., 2017; Czabotar et al., 2013). Mitochondrial dynamics, energy sensing, and Bcl-2 family regulation produce stronger systemic effects by combining organelle integrity with cell fate determination. Among source-control strategies, CAPE and astaxanthin are particularly effective because they can reduce ROS leakage and improve bioenergetic efficiency (Ali et al., 2020; Yan et al., 2024). Potential indication-oriented combination regimens include: acute oxidative stress, such as ischemia–reperfusion injury, may benefit from rapid clearance of ROS and enzyme induction, for example, CAPE combined with EGCG/SFN, whereas chronic neurodegenerative diseases may be more suitable for combined ETC support, mitochondrial dynamics stabilization, and Bcl-2 upregulation (Andreu Fernández et al., 2020; Langston-Cox et al., 2020; de Oliveira et al., 2015). Reasonable synergistic strategies may also include the combined use of ETC optimizers, such as CAPE, with mitochondrial stabilizers, such as baicalin, to reduce ROS generation and the risk of structural vulnerability, thereby stabilizing the defense network (Ali et al., 2020; de Oliveira et al., 2015). Similarly, combining an Nrf2 activator, such as SFN, with mitophagy inducers, such as low-dose quercetin, may enhance antioxidant capacity while removing damaged mitochondria, thereby constructing a closed-loop defense mechanism. In addition, the lipophilicity of astaxanthin enables it to form spatial complementarity with water-soluble antioxidants such as SFN (Yan et al., 2024; Langston-Cox et al., 2020).

In summary, natural products enhance antioxidant enzyme systems (SOD, CAT, HO-1, and NQO1), improve electron transport chain efficiency to reduce ROS leakage, maintain mitochondrial membrane potential (ΔΨm) and membrane integrity, rebalance fission–fusion dynamics, regulate MOMP through the Bcl-2 family, and reshape redox signaling networks (such as the Nrf2 pathway), thereby building a powerful mitochondrial defense system. These multidimensional and multi-target mechanisms jointly maintain mitochondrial homeostasis. However, their efficacy depends on the dosage and the route of administration. Tannic acid can scavenge ROS at low micromolar concentrations but may increase membrane permeability at higher concentrations. The plasma peak concentration (Cmax) of intravenous quercetin is approximately 2.3 μg/mL, which may have neuroprotective effects, but its oral bioavailability is low, approximately 1–5%, which limits its therapeutic concentration in the brain (Vafadar et al., 2020; Rayamajhi et al., 2013). The lipophilicity of astaxanthin facilitates its distribution in the mitochondrial membrane and may support the function of complex I, but it usually requires an oral dose above approximately 20 mg/kg to achieve a detectable tissue concentration, which remains a challenge in translational applications (Mularczyk et al., 2022) (Supplementary Table 1).

### 4.8 Synergy and integration: structure–activity relationships and mechanism crosstalk

The regulation of mitochondrial dynamics by natural products mainly depends on the product’s chemical structure and involves a variety of mechanisms. Core chemical characteristics, such as the number and location of phenolic hydroxyl groups in polyphenolic metabolites, the glycosylation sites of flavonoids, and the charge density of alkaloids, jointly determine their affinity for targets, subcellular localization, and residence behavior at the mitochondrial membrane. An in-depth understanding of these structure–activity relationships (SARs) is the basis for rational optimization. Key signaling pathways, including AMPK/SIRT1-PGC-1α, integrate mitochondrial biogenesis, dynamics, and autophagy, thus achieving multidimensional synergistic regulation. However, dose-dependent biphasic effects (i.e., hormetic effects or mitohormesis), signal reprogramming in the pathological microenvironment, and structural changes caused by primary metabolism are still the main obstacles to clinical translation. Future research should be committed to achieving a predictable and controllable balance between multi-target efficacy and off-target toxicity through pharmacological and medicinal-chemistry strategies, such as rational glycosylation control, charge shielding, and mitochondrial targeting delivery.

#### 4.8.1 Structure–activity relationships and target selectivity

The regulation of mitochondrial dynamics by natural products shows a close structure–function relationship, and their structural determinants regulate mitochondrial localization, target selectivity, pharmacokinetics, and dose-dependent effects in a hierarchical manner (Figure 8).

**FIGURE 8:**
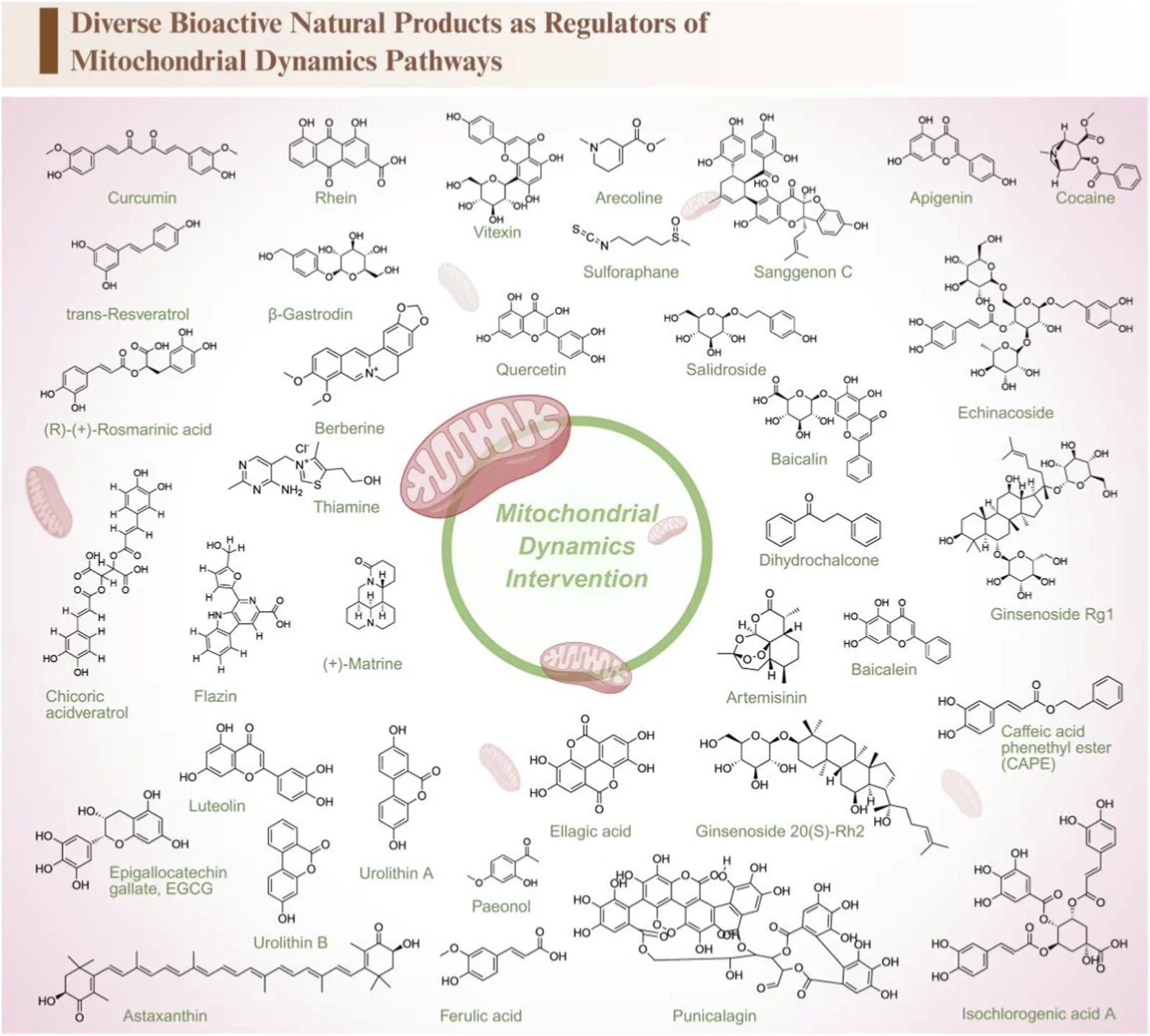
A spectrum of molecular architectures: the diverse structures of natural product regulators targeting pathways of mitochondrial dynamics. The metabolites include trans-resveratrol, curcumin, berberine, epigallocatechin gallate (EGCG), ginsenoside Rg1, rosmarinic acid, chicoric acid, isochlorogenic acid A, β-gastrodin, flazin, cocaine, vitexin, apigenin, quercetin, baicalein, sanggenon C, baicalin, salidroside, echinacoside, ginsenoside 20(S)-Rh2, artemisinin, luteolin, dihydrochalcone, thiamine, caffeic acid phenethyl ester (CAPE), ellagic acid, ferulic acid, sulforaphane, arecoline, punicalagin, urolithin A, and urolithin (B). These natural products are divided into polyphenols, alkaloids, flavonoids, glycosides, and other major categories according to their chemical scaffolds and biological activity related to the regulation of mitochondrial dynamics. The structure of these metabolites is shown in the article to facilitate identifying and comparing the key functional groups involved in mitochondrial targeting intuitively.

Polyphenolic metabolites exhibit a unique structure–effect relationship, which is mainly driven by phenolic hydroxyl groups and conjugated systems. This shows how chemical structure determines pharmacological effects and pharmacokinetic characteristics. For example, the conjugated system of curcumin and its β-diketone skeleton may affect electron transfer and the generation of ROS under certain conditions, thus affecting mitochondrial redox balance (Zhang et al., 2019). Tannic acid contains multiple phenolic hydroxyl groups, which can quickly neutralize hydroxyl radicals (·OH) and stabilize mitochondrial membrane potential (ΔΨm), but excessive concentration may increase membrane permeability. The interaction between the number of hydroxyl groups and hydrophobicity determines its safe concentration range (Teel et al., 1986).

The core C_6_–C_3_–C_6_ flavonoid skeleton further reflects fine-tuned regulation through hydroxylation and glycosylation. The water solubility of vitexin is improved by the presence of a C-8 glucosyl group, and NOX4 can be inhibited through the Epac1/Rap1 pathway, thereby reducing the production of O_2_^−^ and upregulating MFN2 (Chen W. et al., 2023; Yang et al., 2021). In contrast, deglycosylated derivatives may lose mitochondrial protective activity, suggesting that glycosylation can influence solubility, distribution, and target engagement. The 5,7-dihydroxyl structure of luteolin may allow it to interact with antioxidant or mitochondrial regulatory proteins; however, its overall lipophilicity makes it easier to insert into cholesterol-rich erythrocyte membrane domains, and concentrations exceeding 20 μM may cause dose-dependent hemolytic effects. The characteristics of baicalein highlight the potential role of a single hydroxyl group: its 7-position hydroxyl group (7-OH) may form hydrogen bonds involved in interaction with regulatory proteins such as KLF4, thereby influencing the KLF4-MARCH5-DRP1 axis (Zacharioudakis et al., 2022). If this site undergoes methylation or glycosylation, the above activity may be lost (Chen W. et al., 2023).

Alkaloids use the charge state and stereochemical properties of nitrogen-containing heterocycles to achieve selective mitochondrial anchoring. Berberine, a cationic isoquinoline alkaloid, can accumulate in mitochondria driven by the negative mitochondrial membrane potential (ΔΨm = −180 mV), inhibit complex I, increase the AMP/ATP ratio, and activate the AMPK-mTOR-ULK1 autophagy axis. However, this cationic feature also promotes interaction with negatively charged residues (T623/S624) near the outer vestibule of the hERG channel, thereby increasing the risk of IKr blockade and QT prolongation at high doses (Zhao et al., 2019). The rigid quinolizidine ring of matrine is considered to interact with the Ca^2+^–OMA1 interface, thus preventing OMA1-dependent cleavage of L-OPA1 and maintaining mitochondrial fusion. When the biological drug intake exceeds 10 g, CYP3A4 can oxidize it into lipophilic N-oxides that accumulate in hepatocyte mitochondria, resulting in sharp ALT/AST increases and indicating that nitrogen oxidation state and steric effects are shared determinants of the toxicity threshold (Yapa et al., 2021).

In a word, structural determinants hierarchically regulate the targeting, selectivity, pharmacokinetics, and dose-dependent effects of mitochondria, thus providing a quantifiable basis for rational optimization.

#### 4.8.2 Mechanism crosstalk: multidimensional synergy and bidirectional regulation of hub pathways

The regulation of natural products centers on three interrelated pathways: the AMPK/SIRT1-PGC-1α signaling pathway, the mitochondrial fission–fusion–mitophagy triad, and the antioxidant-dynamics feedback loop. These three pathways are highly sensitive to dose and microenvironment, which leads to bidirectional regulation (mitohormesis) (Yang et al., 2024). This theory proposes that many substances show beneficial or stimulatory effects at low doses but inhibitory or toxic effects at high doses. Specifically, mitohormesis describes a process in which mild mitochondrial stress (for example, a slight increase in ROS or a brief decrease in ATP) triggers adaptive cellular stress responses (for example, activation of NRF2, AMPK, or SIRT1), ultimately enhancing mitochondrial function, biogenesis, and antioxidant capacity, thereby conferring long-term resilience (Yang et al., 2024).

This theory provides a key framework for understanding the dose-dependent biphasic effects of many natural products, especially by helping to explain the difference between the strong *in vitro* effects observed at high micromolar concentrations and the beneficial effects observed at much lower systemic exposure levels. This concept is crucial to the mechanism of action of many natural products because they can slightly perturb mitochondrial processes at physiologically achievable low concentrations. For example, resveratrol may affect mitochondrial complexes I and III, while high-dose EGCG induces electron transport chain dysfunction. These effects are more likely to produce mild adaptive stress signals than to lead to catastrophic system failure.

EGCG is a typical example of this principle. At low concentrations (such as 5 μM), EGCG protects cells by activating protective pathways such as NRF2, thus enhancing the antioxidant defense capacity of cells (Valenti et al., 2013). However, at high concentrations (for example, 50 µM or higher), EGCG may be transformed into a pro-oxidant and lead to hepatotoxicity (Andreu Fernández et al., 2020; Hazimeh et al., 2023; Capasso et al., 2025). This transformation from protection to damage reflects the characteristic curve of mitohormesis. Similarly, betel nut triggers protective autophagy through the ROS signaling pathway at low doses, but high doses lead to oxidative damage and cell death (Wang S. et al., 2023). The principle of mitohormesis is of great significance: for many natural products with low bioavailability, high doses are not necessarily better. This provides a theoretical basis for the dose-dependent effects observed for many natural products and emphasizes the importance of detailed dose exploration in preclinical and clinical studies. The research focus should shift from simply pursuing the maximum tolerated dose (MTD) to identifying and verifying the hormetic dose window.

In the ternary balance of mitochondrial fission, fusion, and mitophagy, natural products act as network regulators, and their mechanisms of action usually depend on the specific environment. Artemisinin relies on the cleavage of its internal peroxide bridge triggered by Fe^2+^ to generate instantaneous ROS pulses, which are sufficient to activate the PINK1/Parkin pathway and remove damaged mitochondria; when the dose exceeds about twice the IC_50_, cytoplasmic ROS overload may induce apoptosis (Zhu et al., 2022).

Under neuroinflammatory conditions, luteolin inhibits BNIP3L/NIX-mediated mitophagy, thus preventing excessive neuronal loss (Vongthip et al., 2024). In contrast, quercetin enhances mitophagy through the SIRT1/PINK1/Parkin axis, showing tissue-specific bidirectional regulation (Li F. et al., 2021). Curcumin further emphasizes this context dependence: in hepatocellular carcinoma cells, it promotes apoptosis by inhibiting DRP1 oligomerization, whereas in normal hepatocytes, it enhances mitochondrial fusion through the AMPKα2/PGC-1α axis, thus maintaining liver function (Otuechere et al., 2014). Therefore, the pathophysiological state can alter the direction of the functional effects of the same molecule.

The third-layer mechanism links antioxidant defense with mitochondrial dynamics through a feedforward amplification loop. Caffeic acid phenethyl ester (CAPE) can enhance the electron throughput of complex I, thus reducing the basal ROS level and indirectly reducing the phosphorylation of DRP1 at Ser616 (Ali et al., 2020). Isochlorogenic acid A can simultaneously activate the Nrf2/LC3-II axis and upregulate OPA1/MFN2, thus integrating antioxidant signals with mitochondrial dynamics and producing a synergistic amplification effect (Lai et al., 2024). The mitohormetic effect of betel nut determines the dose threshold of this coupling: at 10 nM, ROS acts as a survival signal and triggers protective autophagy; when the concentration exceeds 1 μM, the level of ROS exceeds the buffering capacity, resulting in mitochondrial permeability transition pore (mPTP) opening and initiation of the cell death program (Wang S. et al., 2023; Nunnari and Suomalainen, 2012). Usually, natural products regulate mitochondrial homeostasis from multiple dimensions, such as structure, microenvironment, and dose dependence, by acting on the AMPK/SIRT1/PGC-1α axis, the fission–fusion–mitophagy triad, and the antioxidant-dynamics feedback loop. They usually follow the principle of mitohormesis, which provides a quantitative framework for target selection and therapeutic window optimization.

The regulatory effect of natural products is also limited by their inherent chemical properties: the number of phenolic hydroxyl groups, the glycosylation topology of flavonoids, and the charge density of alkaloids jointly determine their target affinity and subcellular distribution. Key nodes such as AMPK/SIRT1/PGC-1α integrate mitochondrial biogenesis, dynamics, and mitophagy into a coordinated multidimensional regulatory network. The dose-dependent biphasic effect, signaling reprogramming in the pathological microenvironment, and first-pass metabolic transformation are still the main obstacles to clinical translation. Potential solutions include glycosylation regulation, charge shielding, and mitochondrial-targeted delivery strategies to achieve a predictable balance between multi-target efficacy and off-target toxicity.

#### 4.8.3 Comparative perspective with representative clinical drugs

Natural products modulate mitochondrial homeostasis through concurrent engagement of morphological dynamics, antioxidant defense, energy metabolism, and autophagic quality control. However, this polypharmacological advantage remains constrained by unpredictable pharmacokinetics and limited exposure monitoring. Approved clinical agents demonstrate that translational success depends less on mechanistic breadth than on rigorous pharmacokinetic and pharmacodynamic characterization coupled with precise exposure control. Metformin targets only the AMPK/PGC-1α axis yet achieves reliable clinical outcomes through linear dose–exposure relationships and estimated glomerular filtration rate-guided dosing that minimizes lactic acidosis risk (Zhang et al., 2016). Edaravone serves narrowly as a radical scavenger but benefits from defined cerebrospinal fluid partitioning and short-term infusion protocols ensuring consistent central nervous system exposure (Soejima-Kusunoki et al., 2022). Idebenone offers high oral bioavailability and rapid blood–brain barrier penetration, pharmacokinetic attributes that most natural products, including resveratrol with approximately 6% bioavailability, fail to achieve (Javadov et al., 2018). Rapamycin drives autophagy via precise mechanistic target of rapamycin complex one inhibition, with its therapeutic window safeguarded by immunosuppressive monitoring protocols rather than mechanistic refinement (Wang P. et al., 2024). These examples illustrate that the translational gap for natural products stems not from inferior mechanistic potential. Baicalein simultaneously modulates the KLF4/MARCH5/DRP1 axis and antioxidant signaling (de Oliveira et al., 2015; Hu et al., 2022), resveratrol constructs sustained antioxidant networks through SIRT1-mediated deacetylation and Nrf2-driven phase II enzyme induction (Meza-Torres et al., 2020; Xing et al., 2024), astaxanthin improves oxidative phosphorylation efficiency by increasing core respiratory chain subunits (Yan et al., 2024), quercetin enhances protective autophagic flux through the SIRT1/PINK1/Parkin cascade (Vafadar et al., 2020), and urolithin A couples PINK1/Parkin-dependent mitophagy with peroxisome proliferator-activated receptor gamma coactivator 1-alpha-mediated biogenesis (Kern et al., 2025). Rather, the translational gap is a consequence of the absence of standardized pharmacokinetic and pharmacodynamic frameworks, biomarker-guided dosing strategies, and delivery systems capable of stabilizing exposure within hormetic therapeutic windows. Future natural product development should therefore prioritize adopting pharmacokinetic and pharmacodynamic modeling paradigms from approved drugs to define exposure–efficacy relationships, integrating nanodelivery or structural modification to improve bioavailability and tissue selectivity, and establishing mechanism-based safety monitoring rather than relying on empirical dose escalation. These translational adaptations, discussed further in Section 5, are essential to translate the multi-target potential of natural products into clinically viable mitochondrial therapies.

## 5 The pharmacokinetic-safety paradox: bridging the gap between *in vitro* efficacy and clinical translation

The preceding sections have systematically cataloged the multidimensional regulatory mechanisms through which natural products modulate mitochondrial dynamics, with each compound discussed explicitly anchored to the Core Paradox articulated in the Introduction: the persistent disconnect between *in vitro* effective concentrations (typically 5–50 µM) and achievable human systemic exposure (often an order of magnitude lower). This pervasive PK/PD mismatch is not merely a pharmacokinetic inconvenience but the central translational challenge that constrains the clinical viability of every mechanism described above. Low oral bioavailability, rapid biotransformation, and tissue-selective distribution determine whether effective concentrations can reach mitochondrial targets. For example, the *in vitro* DRP1 inhibition threshold usually exceeds 5 μM; without a specific strategy, such concentrations of natural products cannot be maintained *in vivo*. This section now turns from mechanistic description to critical analysis, examining how pharmacokinetic parameters determine whether, and under what conditions, these mechanisms can be translated into clinical reality.

### 5.1 Pharmacokinetic characteristics and bioavailability challenges

The effect of regulating mitochondrial dynamics is closely related to PK characteristics. Pharmacological outcomes, such as DRP1 inhibition and PGC-1α activation, largely depend on whether drug concentrations reach threshold levels at mitochondrial target sites (Figure 9). A core recurring problem is that therapeutic efficacy is often limited by a pronounced PK/PD disconnect, which is reflected in extremely low oral bioavailability, rapid clearance, and metabolic instability, making it difficult to maintain drug concentrations above effective levels at the target site (Bonelli et al., 2019). However, structure-based optimization and advanced delivery technologies can significantly improve clinical translation potential. Establishing a reliable translational PK/PD model is crucial to bridge the gap between preclinical observation and clinical efficacy. The following sections will discuss representative natural products and classify them according to their bioavailability and metabolic challenges.

**FIGURE 9:**
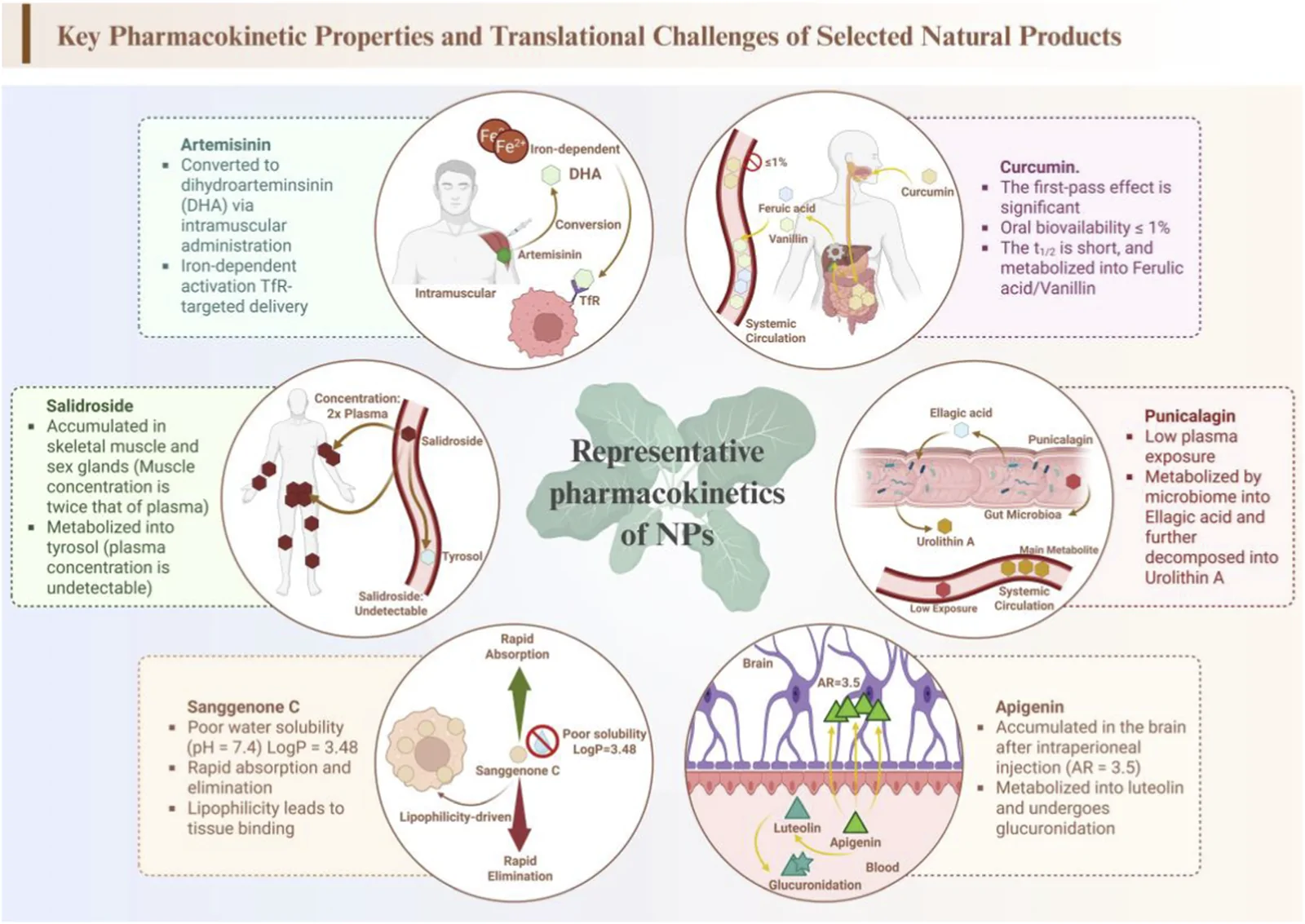
From laboratory to clinic: key pharmacokinetic characteristics and transformation disorders of natural products. This schematic summarizes key absorption, distribution, metabolism, and excretion (ADME) properties of selected natural products. Artemisinin is converted into dihydroartemisinin (DHA) after intramuscular administration and undergoes iron-dependent activation involving targeted delivery to the transferrin receptor (TfR). Curcumin shows a pronounced first-pass effect; oral bioavailability is only ∼1%, the half-life is short, and metabolites include ferulic acid and vanillin. Salidroside accumulates in skeletal muscle and gonadal tissue (muscle concentrations are approximately twice those in plasma) and is metabolized into tyrosol. Punicalagin shows low plasma exposure and is metabolized by the gut microbiota. Sanggenon C, with poor water solubility (logP = 3.48), is rapidly absorbed and eliminated. After intraperitoneal administration, apigenin shows a brain accumulation ratio (AR) of 3.5 and is metabolized into luteolin, followed by glucuronidation. These examples illustrate the diverse pharmacokinetic challenges faced in the development of natural products.

#### 5.1.1 First-pass effect dominant type

These metabolites undergo extensive intestinal and/or hepatic first-pass metabolism after oral administration, resulting in extremely low bioavailability of the parent metabolites, which is a major obstacle limiting their therapeutic effects. Curcumin is a typical representative of such metabolites: its water solubility is approximately 11 ng/mL, and first-pass metabolism is significant, resulting in oral bioavailability in rats of only approximately 1%, with a relatively short half-life (T_1/2_ = 11.96 ± 2.64 min). The β-diketone structure in the curcumin skeleton is prone to cleavage under near-neutral or alkaline conditions, producing vanillic acid and vanillin, thus further reducing its chemical stability (Jelezova et al., 2015). In addition, there are significant differences in bioavailability, tissue distribution, and related pharmacokinetic parameters among different administration routes. In humans, its oral bioavailability is even lower, approximately 0.001–0.04%. Even if the dose reaches 8 g, the plasma peak concentration (C_max_) is only 1.7 μM, which is far lower than the typical *in vitro* effective concentration of 5–50 μM (Wang B. et al., 2023).

This difference in bioavailability is also closely related to species-specific metabolic characteristics: in humans, 3′-O-glucuronidation is the main metabolic pathway, while in rats, 4′-O-glucuronidation dominates (Khatoon and Kalam, 2025; Liu et al., 2022). This significant difference indicates that extra care is needed when extrapolating preclinical findings to humans and also highlights the necessity of developing advanced drug-delivery systems.

The oral bioavailability of resveratrol is approximately 6%, limited by extensive glucuronidation and sulfation during first-pass metabolism. Human studies show that after a single oral administration of 500 mg, the absorption rate is relatively high, approximately 75%. However, due to rapid metabolism in the intestine and liver, the circulating concentration of the conjugated metabolites is much higher than that of the parent metabolite (i.e., free resveratrol) (Martínez-Abundis et al., 2016; Sergides et al., 2016; Kapetanovic et al., 2010). This means that the metabolites are more widely distributed in the body. Intravenous injection of 5 mg/kg can bypass first-pass metabolism, so as to achieve rapid systemic exposure and tissue distribution. Note that resveratrol can pass through the BBB and reach a measurable concentration in the brain, which supports the view that it may have a neuroprotective effect (Cerqueira et al., 2025). Species-specific differences are also evident: in rats, the apparent volume of distribution is substantial (V_d_: intravenous, 5.8 L/kg; oral, 23.3 L/kg), and the elimination half-life is prolonged (∼7–9 h), consistent with extensive tissue distribution and reduced clearance.

Quercetin is characterized by a low absorption rate, fast phase II metabolism, and significant intestinal and hepatic circulation. After healthy volunteers took 8 mg, 20 mg, or 500 mg of quercetin, the oral bioavailability was only 1–5%, C_max_ = 2.3 ± 1.5 μg/mL, and T_max_ = 0.7 ± 0.3 h. After absorption, quercetin is distributed in the liver, kidneys, and intestines, whereas BBB penetration is weak. Reported pharmacokinetic parameters include distribution half-life (T_1/2_, distribution = 0.7–7.8 min), elimination half-life (T_1/2_, elimination = 3.8–86 min), clearance (CL = 0.84 L/min/m^2^), and volume of distribution (V_d_ = 3.7 L/m^2^) (Vafadar et al., 2020; Rayamajhi et al., 2013).

ICGA shows moderate absolute bioavailability in rats (calculated: 30.71%; measured: 22.6%), but this is reduced by intestinal and hepatic first-pass effects (Behne et al., 2023). Intestinal absorption varies by segment, with the ileum showing the highest uptake. ICGA transport appears to occur mainly through passive diffusion within a specific small-intestinal absorption window and does not become saturated at high concentrations (Wang et al., 2020). However, the passive-diffusion mechanism of ICGA remains unclear. Its metabolic processes include hydrolysis, dehydroxylation, hydrogenation, and conjugation (Wang et al., 2021). The metabolite spectrum depends on the route of administration: after intravenous injection, cyclic metabolites N1, N2, N4, and N5 dominate during the first 30 min, with N1 predominating from 0.5 h to 4 h, followed by N8, while N6 is also detected; after oral administration, N6 and N8 become the major circulating species.

These differences reflect the rapid biotransformation of ICGA through plasma and liver enzymes in the systemic circulation. Its pharmacokinetic characteristics are almost linear, and metabolites such as N5 and N9 have been detected in plasma, urine, feces, and *in vitro* intestinal flora systems (Tian et al., 2017). At present, it is not clear whether the differences in metabolic profiles under different administration routes will lead to differences in PD effects, such as differences between N1 and N8 in mitochondrial fusion activity. This constitutes a key knowledge gap in understanding the therapeutic potential of ICGA under different routes of administration.

Matrine shows significant route dependence. Its oral bioavailability is limited by the first-pass effect (dogs: 19.4%; rats: 31.6–47%), while intramuscular injection shows high absolute bioavailability, approximately 104.43%. In humans receiving an intravenous dose of 6 mg/kg, its pharmacokinetic characteristics are consistent with a biphasic model. T_1/2_α is 19.21 min, and T_1/2_β is 184.22 min. Matrine is distributed in the liver, spleen, and kidney, and only a very small amount enters the brain and adipose tissue. approximately 52.75% of the intravenous dose is excreted through the kidneys, and the renal clearance rate is approximately 143.79 mL/min (You et al., 2020).

#### 5.1.2 Transporter- and microbial metabolism-dependent type

The absorption, metabolism, and pharmacological activity of these metabolites are highly dependent on intestinal transport proteins and intestinal microbiota. Because the systemic exposure of parent metabolites is usually low, the research focus tends to shift to biologically active metabolites. The oral bioavailability of EGCG is approximately 0.1–0.3%. Its pharmacokinetic characteristics include rapid absorption, extensive tissue distribution, rapid metabolism, and renal excretion. EGCG is mainly absorbed in the gastrointestinal tract and remains stable in the acidic environment (pH < 3) of the stomach, while degradation occurs in the alkaline environment of the small intestine. Its biotransformation encompasses microbially mediated conversion to bioactive phenyl-γ-valerolactone metabolites, as well as hepatic methylation, sulfation, and glucuronidation, followed by renal excretion (Pervin et al., 2019; Andreu Fernández et al., 2020; Capasso et al., 2025). Intake conditions have a significant impact on exposure: taking high-purity extracts on an empty stomach can improve bioavailability and accelerate absorption, but individual differences are still significant; in contrast, taking EGCG with food will significantly reduce exposure, which may be related to changes in gastric emptying and digestive juice secretion.

Some formulations, such as those containing vitamins or fatty acids, may reduce apparent bioavailability while improving chemical stability (Andreu Fernández et al., 2020). In general, the matrix, intake time, and dosage form jointly determine the pharmacokinetic behavior of EGCG.

For polyphenols, microbial metabolism is particularly important. The gut microbiota–mitochondria axis discussed in Section 4.6.4 is essentially mediated through these pharmacokinetic processes. UA is generated from ellagitannins through microbial metabolism, which largely determines the systemic effects of the parent precursors and reflects the pharmacokinetic role of the gut microbiota–mitochondria axis. Punicalagin is a typical example: after oral administration, because of low bioavailability and/or rapid systemic clearance, plasma exposure to the parent metabolite is extremely low and often difficult to detect. In a trial of 250 mg or 1,000 mg standardized pomegranate extract (Pomella®, containing ≥30% punicalagins) in healthy volunteers, 48-h UHPLC–MS/MS analysis showed that punicalagins were hydrolyzed into ellagic acid (EA). EA is then converted by the gut microbiota (such as *Gordonibacter* spp.) into urolithins, including UA and urolithin B; conjugated urolithins appear after approximately 8 h, peak at approximately 24 h, and dominate in circulation. Unconjugated UA is detectable only in a few individuals, whereas urolithin B is usually difficult to detect. This process shows marked interindividual variability. According to microbial metabolic capacity, individuals can be classified into different urolithin metabotypes (such as producers and non-producers). However, the relationship between microbial conversion efficiency and the PD effects of UA (for example, mitophagy induction) has not yet been quantified: it remains unclear whether a certain level of UA is required to activate PINK1/Parkin signaling, thereby limiting the development of metabolism-guided personalized dosing. This highlights the necessity of metabolism-oriented clinical strategies. Without prescreening or stratification, clinical trial cohorts will include both responders (for example, metabotype A) and non-responders (for example, metabotype 0), thereby diluting the overall therapeutic effect and potentially leading to false-negative conclusions. The area under the curve (AUC) of conjugated metabolites is fivefold to eightfold higher than that of unconjugated metabolites and shows delayed, metabolism-dependent cyclic characteristics, highlighting the microbiota dependence and rapid conjugation characteristics of this process (Wang Y. H. et al., 2023). In general, the pharmacokinetic profile of punicalagin is characterized by extremely low direct exposure, microbiome-dependent generation of indirect active metabolites, and substantial interindividual variability.

Interindividual differences in the ability to generate UA, namely the metabotype, highlight the central role of microbiome composition in determining therapeutic response. The abundance of *Gordonibacter* is related to circulating UA levels. This forms a closed loop with the mitophagy-related mechanisms described in Section 4.3: although microbial metabolites such as urolithin A have relatively high bioavailability, individualized administration strategies are still required because of interindividual differences, and probiotic intervention may help optimize the intestinal ecosystem. The translational significance is obvious: the metabolic type is not a secondary covariate, but a key determinant of the therapeutic response. Therefore, future clinical research must regard the composition of gut microbiota as a key variable. This can be achieved through pre-treatment metabolic stratification or interventions aimed at standardizing microbial metabolites, such as the combined use of specific probiotics. The same principle applies to other chemical skeletons, including microbiota-mediated glycoside hydrolysis.

*In vivo*, xanthine is hydrolyzed by intestinal β-glucosidase to produce anthrane, which has pharmacological activity and shows characteristic distribution and elimination kinetics, including a relatively long half-life, thus supporting continuous exposure. The route of administration has a significant impact on these parameters: oral administration is usually associated with slow absorption and low bioavailability, while intravenous administration can achieve rapid distribution and faster elimination kinetics (Hu et al., 2022).

#### 5.1.3 Blood–brain barrier penetrating type

Even with low systemic bioavailability, some natural products can pass through the BBB, which is crucial for CNS indications. Apigenin is a typical example: although its oral bioavailability is low and it undergoes extensive first-pass metabolism and enterohepatic circulation, it can still effectively enter the brain and remain in the brain tissue for a long time (Socała et al., 2025). After intraperitoneal injection of 50 mg/kg in mice, the serum and brain concentrations reached their peaks simultaneously (T_max_ = 0.25 h), indicating rapid penetration across the BBB. Elimination kinetic data further confirm drug accumulation in brain tissue: the brain half-life is 48.2 h, while the serum half-life is only 12.86 h. After repeated administration at 50 mg/kg for 14 consecutive days, the predicted trough concentration reached 89.92 ng/g, and its accumulation ratio (AR = 3.5) was about 2.5 times the serum accumulation ratio. Apigenin is metabolized by CYP1A1, CYP2B, and CYP2E1 to generate hydroxylation products, such as luteolin, and then undergoes glucuronidation. After intraperitoneal administration, its apparent clearance (CL/F = 19.33 L/h/kg) exceeds hepatic blood flow, approximately 5.4 L/h/kg, indicating substantial extrahepatic metabolism (Socała et al., 2025).

Echinacoside shows completely different characteristics: systemic exposure after oral administration is extremely low. Although there is evidence that it can penetrate the BBB, this permeability appears to be condition-dependent. In rats, its absolute oral bioavailability is only approximately 0.83%, and the parent metabolite is usually not detected in plasma. Human studies also confirmed this finding (Liu et al., 2018). Caco-2 cell experiments show that its apparent permeability coefficient is 10^−7^ cm/s, indicating poor epithelial permeability. After an oral dose of 100 mg/kg in rats, the peak time (T_max_) is 15 min, indicating that even when exposure is low, absorption may be very fast. Its biotransformation involves intestinal flora-mediated glycoside hydrolysis, and then further metabolism into related derivatives. In the human body, rapid phase II conjugation reactions, including sulfation and glucuronidation, limit the circulating concentration of the parent metabolite to the nanomolar range. Tissue distribution studies show that under cerebral ischemic conditions, echinacoside can pass through the blood–brain barrier, while under physiological conditions, its permeability is limited. Echinacoside is mainly excreted through the kidneys. In normal rats, its half-life (T_1/2_) was 74.4 min, while in Parkinson disease model rats, the half-life was extended to 121.6 min; at the same time, the drug clearance rate decreased, and the area under the AUC increased by approximately 35%, indicating that its pharmacokinetic characteristics were affected by the disease state (Liu et al., 2018). Generally speaking, echinacoside has the characteristics of low oral bioavailability and fast metabolism, and its distribution in the body is significantly affected by the pathological state.

#### 5.1.4 Protein-binding-dominant type

Such metabolites usually have a high plasma-protein binding rate, which limits the proportion of free drugs, regulates drug distribution, and affects their pharmacological activity. Sanggenon C is a typical example that shows how physicochemical properties form an absorption barrier: within the physiological pH range, its water solubility is low and shows significant pH dependence.

At pH 7.4, its solubility is only about one-tenth that of its stereoisomer sanggenon D, thus limiting oral absorption (Langeder et al., 2023). Its wide pKa range (2.64–12.05) indicates that it is partially ionized under physiological pH, and its logP value of 3.48 indicates moderate lipophilicity. In multi-metabolite extracts such as MA60, its solubility is further reduced, which is consistent with intermolecular interactions that hinder dissolution. In BALB/c mice orally administered MA60 extract (6.9% sanggenon C), systemic exposure was dose dependent yet low overall, with rapid clearance and no evidence of accumulation. At a dose of 100 mg/kg, the serum concentration ranged from 8.00 ng/mL to 124.22 ng/mL within 1 h after administration, but it dropped to 2.77 ng/mL or below the detection limit after 24 h. Tissue analysis shows that the concentration of the drug in the lungs is extremely low. No signals are detected in the spleen, and the recovery rate in the liver is only 26%, indicating that lipophilicity promotes rapid tissue binding and/or biotransformation of the drug. In the monolayer model of Calu-3 bronchial epithelium, when the sanggenon C concentration was 50 μg/mL, cellular uptake at 4 h and 24 h was 2.0 ± 0.1 pg/cell and 8.1 ± 0.3 pg/cell, respectively, consistent with passive diffusion. However, its apparent permeability is low, and bidirectional transport is negligible, indicating that its transepithelial transport ability is poor, suggesting that inhalation administration may be more suitable for prolonging the lung residence time (Schwarzinger et al., 2024).

Rosmarinic acid is transported through the paracellular pathway, and its extensive binding with plasma proteins, such as human serum albumin, may help form a circulating drug reservoir. Gastric acid can reduce the amount of recoverable metabolite by more than 50%, while its role in the small intestine is relatively limited. The intestinal flora can degrade rosmarinic acid into simpler phenolic acids, such as caffeic acid and valonic acid, thus promoting its absorption. Subsequently, rosmarinic acid is metabolized in the liver through sulfation, glucuronidation, and methylation, in which caffeic acid is further converted into protocatechuic acid, vanillic acid, and p-hydroxybenzoic acid.

After oral or intravenous administration, the parent metabolite rapidly appears in plasma and quickly reaches peak concentration (C_max_); food intake slightly reduces C_max_ and prolongs the time to peak concentration (T_max_). The drug is excreted through the kidneys. Most metabolites are cleared within 6 h, and trace metabolites can still be detected after 24–48 h (Nunes et al., 2015). In rats, reported pharmacokinetic parameters include T_1/2_ = 0.75 h, mean residence time = 0.32 h, clearance = 1.02 L/h/kg, and disproportionately high renal AUC relative to other tissues (Hitl et al., 2020).

The oral bioavailability of ginsenoside Rg1 is very low (1.9–20%), owing to intestinal first-pass metabolism, P-glycoprotein (P-gp)-mediated efflux (Shi et al., 2013), and limited aqueous solubility (Inam et al., 2017; Ni et al., 2015). It is absorbed rapidly and can be detected in plasma within 15 min after oral administration; in rats, T_max_ = 0.5 h, and at 300 mg/kg, the relative bioavailability is approximately 2.5%, based on standardized dose comparison (intravenous AUC = 99.76 μg h/mL; oral AUC = 322.70 μg h/mL) (Ma et al., 2011). It is widely distributed, with the highest concentrations in the liver, kidneys, and heart, and its overall pharmacokinetic profile is biphasic. Metabolism involves oxidation, reduction, and conjugation to generate active derivatives such as ginsenoside Rh1 (GRh1) and PPT. Both retain neuroprotective and anti-inflammatory activity, and PPT has a longer half-life (∼24 h), thereby prolonging pharmacological action (Inam et al., 2017). Only a small fraction of Rg1 is hydroxylated by CYP450, and oxidative metabolites account for less than 5% of the plasma parent-drug concentration. Mass balance studies show that after oral administration, approximately 72% of the total exposure is recovered in feces (parent metabolite: 40.11%), while bile and urine contribute 6.88% and 0.04%, respectively (Ni et al., 2015).

In preclinical animal models (primarily rats), the oral bioavailability of berberine is 0.37% ± 0.11%, which is limited by poor intestinal permeability caused by cationic ionization under physiological pH, P-gp-mediated efflux, intestinal microbial reduction to dihydroberberine, and extensive hepatic first-pass metabolism (Feng et al., 2021). Oral absorption is dose-dependent over the dose range of 48.2–240 mg/kg. The AUC of berberine and its metabolites (M1 and M6–M9) shows an approximately linear increase, but metabolic saturation may lead to nonlinear changes. Berberine is widely distributed in the body, including the liver, kidneys, muscles, lungs, brain, heart, pancreas, and adipose tissue, and tissue concentrations are usually higher than plasma concentrations.

In rats, its apparent volume of distribution (Vz/F) ranges from 12 L/kg to 38 L/kg. Phase I metabolism generates berberrubine (M1), demethyleneberberine (M2), and jatrorrhizine (M3) via CYP2D6, CYP3A4, CYP1A2, CYP2E1, and CYP2C19. In general, phase II glucuronidation (M4, M6, M7, and M9) and sulfation (M5 and M8) dominate, and exposure to phase II metabolites exceeds that of phase I metabolites. Enterohepatic circulation (for example, the biphasic absorption of M7) prolongs systemic residence time. Total excretion through urine, bile, and feces accounts for 41.2% of the recovered dose, of which fecal excretion is dominated by M1 (18.6%), urinary excretion is dominated by glucuronidated metabolites (such as M7), and biliary excretion is relatively low (M1: 2.62%; M7: 0.356%).

#### 5.1.5 Tissue accumulation type

Some natural products accumulate in specific tissues (such as liver, kidneys, or muscle), which in turn affects therapeutic effect and safety. Salidroside is well absorbed in rats and dogs and distributed rapidly. It is characterized by a short T_max_, high C_max_, and a short half-life. Tissue analysis shows that its concentration in muscle is higher than that in plasma, whereas in the brain, liver, and adipose tissue, it is lower (Zhang et al., 2021). Biotransformation occurs through enzymatic hydrolysis of the glycosidic bond to yield tyrosol, followed by further metabolism of the aglycone; circulating metabolites are usually difficult to detect in plasma (Chen et al., 2017). Consistent with this pathway, the amount of urine excretion exceeds the amount of feces and bile excretion, indicating that polar products can be effectively cleared by the kidneys. Ferulic acid has the characteristics of rapid absorption, wide tissue distribution, low oral bioavailability, fast metabolism, fast excretion, and so on (Gong et al., 2022). In rats, after an oral dose of 14.75 mg/kg, C_max_ is reached within 9 min and drops below the detection limit within 120 min; another study using 10 mg/kg also reported T_max_ = 9 min. Compared with oral administration, transdermal administration significantly increases exposure: rat plasma AUC_0−t_ increases to 953.90 ± 175.30 min mg/L versus 281.47 ± 46.76 min mg/L after oral administration; plasma relative bioavailability is 57.9%, and exposure in skin microdialysate reaches 463.4%, indicating prolonged local retention and the possibility of reducing dosing frequency (Yan et al., 2020).

Gastrodin is rapidly absorbed after oral administration (T_max_ = 1–4 h), distributed widely to the liver, kidneys, and heart, and converted to the active metabolite 3,4-dihydroxyphenylethanol, yet still exhibits a relatively short T_1/2_ on the order of hours (Gao H. et al., 2025). After intraperitoneal injection in rats, its dose-dependent pharmacokinetic characteristics can be well described by one-compartment or two-compartment models. After oral administration, gastrodin appears in plasma within 1 h, usually reaching the maximum plasma concentration (C_max_) within 2–4 h; this profile is consistent with its large apparent volume of distribution and extensive tissue penetration (Li R. et al., 2021). Although a lower plasma-protein binding rate may increase the concentration of free drug, it may also limit systemic exposure by increasing clearance. Liver metabolism produces several metabolites, including 4-hydroxybenzyl alcohol. Renal excretion is the main clearance route, while biliary excretion is relatively limited (Gao H. et al., 2025).

In rats, luteolin is usually absorbed quickly and eliminated slowly, and its pharmacokinetics are approximately linear. Its oral bioavailability is low, while intravenous administration can provide higher systemic exposure. The large apparent volume of distribution indicates wide tissue distribution, with accumulation in the liver, kidneys, and lungs. Metabolism produces more water-soluble conjugates through phase II pathways, including glucuronidation and sulfation, thus promoting biliary and urinary excretion and reducing the risk of tissue accumulation (Vongthip et al., 2024).

Neohesperidin dihydrochalcone (NHDC) is a typical dihydrochalcone metabolite. After oral administration of NHDC, it was rapidly absorbed in rats (time to maximum concentration (T_max_) = 0.167 h), but its bioavailability was only moderate (21.75%), which was consistent with the one-compartment model (Liu et al., 2023). After intravenous injection, NHDC is rapidly distributed to the heart, liver, and kidneys and reaches its peak at 0.083 h. Its elimination half-life (T_1/2_) varies depending on the administration route (oral T_1/2_ = 4.7 h; intravenous T_1/2_ = 0.69 h). Due to its low water solubility, absorption may be limited, so nanocarrier formulations may be needed to improve its bioavailability.

#### 5.1.6 Active-metabolite-generating type

In this class of metabolites, the parent metabolite itself has limited activity or poor drug-like properties but can be biotransformed into more active metabolites *in vivo*, thereby shifting therapeutic focus to the pharmacokinetic/pharmacodynamic properties of the metabolites. Artemisinin (ARS) is a sesquiterpene lactone derived from *Artemisia annua* L. Because of its poor aqueous solubility, it has been chemically modified to generate water-soluble derivatives, such as artesunate (ART) and dihydroartemisinin (DHA), the main active metabolite, as well as lipid-soluble analogs, such as artemether (ARM) and arteether. While retaining iron-dependent activation, these modifications improve bioavailability and enable multiple routes of administration. In tumor models, artemisinin derivatives can be targeted to cells overexpressing transferrin receptors (TfR) using holotransferrin or synthetic ligands, such as ART–TfR conjugates. Mechanistically, iron species such as Fe^2+^ can promote cleavage of the endoperoxide bridge and generate cytotoxic free radicals; therefore, pre-incubation with transferrin can enhance DHA cytotoxicity toward Molt-4 leukemia cells. In leukemia xenograft models, such as MOLM-14 and MV4-11, oral or intraperitoneal administration of 25–200 mg/kg artemisinin derivatives can inhibit tumor growth and prolong survival. Notably, artemisinin derivatives are not substrates of P-glycoprotein (P-gp) and can inhibit P-gp function, thereby reversing multidrug resistance; in drug-resistant CEM/ADR5000 cells, artemisinin derivatives enhance the therapeutic effect of doxorubicin by increasing intracellular drug accumulation (Mancuso et al., 2020).

After oral administration of paeonol in rats, paeonol was quickly absorbed and widely distributed. The drug concentration in the liver peaked at 10 min (3460.00 ± 1830.82 ng/g), and measurable levels could be detected in the heart, spleen, kidneys, lungs, and brain (Hu et al., 2020). The metabolite was cleared rapidly from the body (T_1/2_ = 0.68 h), and the tissue concentration decreased significantly within 3 h. Metabolism occurs through demethylation, glucuronidation, hydroxylation, and intestinal microbial hydrolysis (Chen et al., 2018). The metabolites produced include M1, M2, M3, and M4. Among them, the phase I product M2 achieves high plasma exposure at high doses, which may contribute to pharmacological activity. Within 24 h, the total recovery of paeonol and its metabolites in urine, bile, and feces is approximately 35.0%, with urine as the main excretion route; M2 dominates in bile and shows dose-dependent saturation.

In summary, effective mitochondrial intervention requires that natural products overcome persistent pharmacokinetic obstacles. For example, the significant accumulation of berberine in the liver may enhance AMPK activation, but cardiotoxicity may occur when C_max_ > 1 μM due to hERG inhibition; in contrast, the biological activity of resveratrol phase II conjugates is weakened. These examples emphasize the need to adopt pharmacokinetic adaptive strategies, such as microbiome-mediated activation (such as urolithin A) or targeted delivery systems, to balance mitochondrial efficacy and systemic safety. An in-depth understanding of these characteristics is essential, and the assessment of toxicity risks is equally important. These issues will be discussed in detail below.

### 5.2 Safety assessment and toxicity profiles

Safety is a prerequisite for the clinical translation of natural products (Supplementary Table 2). The toxicity spectrum of natural products varies greatly, ranging from good tolerance to specific organ-related risks, and is strictly limited by the pharmacokinetic characteristics described in Section 5.1. Although the multi-target characteristics of natural products may improve the therapeutic effect, they also increase the risk of off-target toxicity. In addition, their effects usually follow the mitohormetic pattern, that is, low doses are beneficial, and high doses are harmful. Low oral bioavailability usually requires higher doses, and extensive first-pass metabolism may produce active or toxic metabolites and increase local tissue exposure, which together change the therapeutic window. Therefore, the dose, administration route, and preparation should be reasonably selected according to the pharmacokinetic characteristics and mitohormetic effects of each natural product to balance efficacy and safety (Figure 10).

**FIGURE 10:**
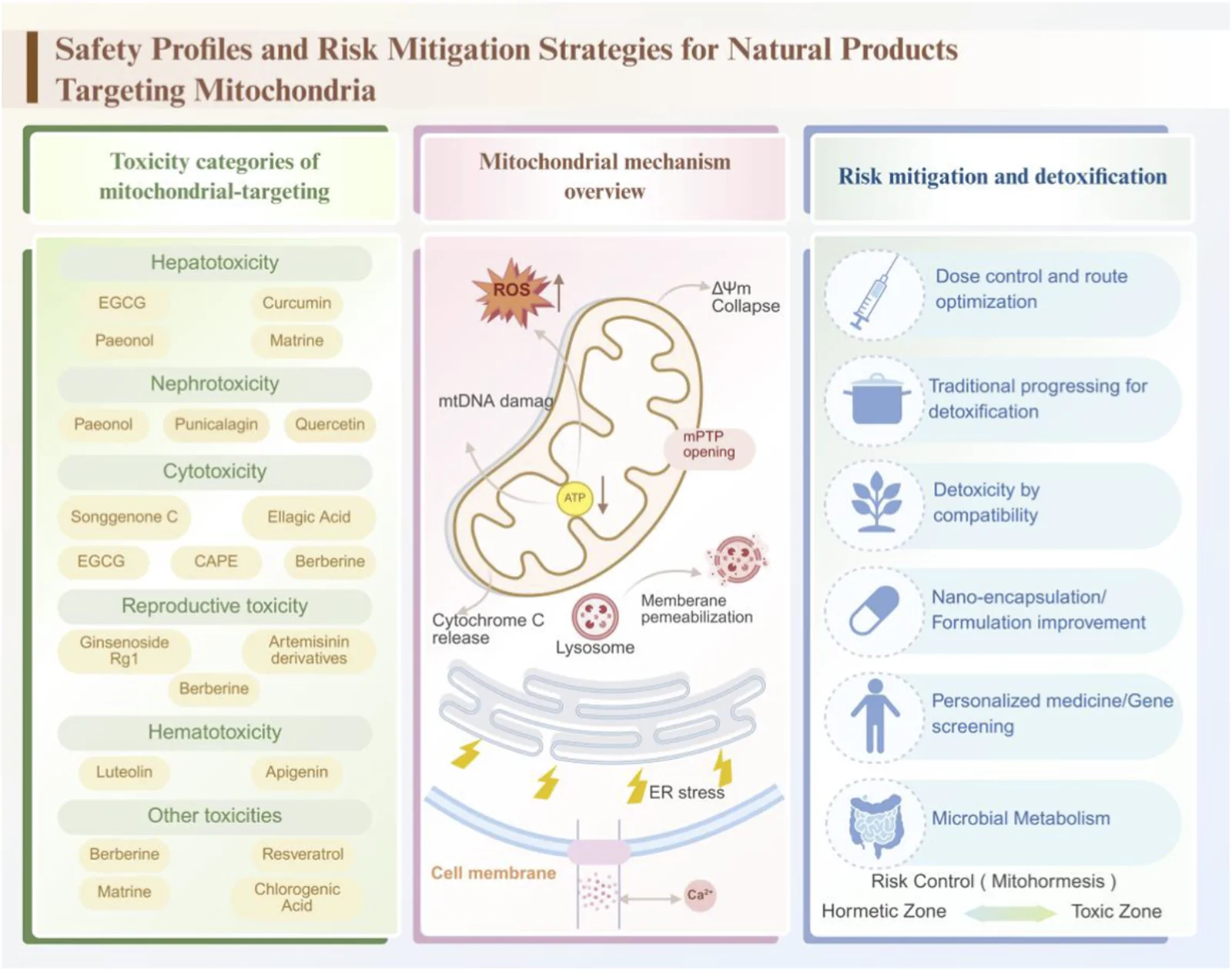
Balancing efficacy and safety: risk profiles and mitigation strategies for mitochondria-targeting natural products. The figure outlines the safety profile of mitochondrial-targeted natural products by integrating the toxicity categories (left) and the corresponding mitigation strategies (right). The left panel shows organ-specific and cellular toxicities: hepatotoxicity (e.g., EGCG, curcumin, paeonol, matrine), nephrotoxicity (e.g., paeonol, punicalagin, quercetin), cytotoxicity (e.g., sanggenon C, ellagic acid, EGCG, CAPE, berberine), reproductive toxicity (e.g., ginsenoside Rg1, artemisinin derivatives, berberine), hematotoxicity (e.g., luteolin, apigenin), and other toxicities (e.g., berberine, matrine, chlorogenic acid). The right panel shows risk management methods: dose control based on the mitohormetic window (mitohormesis), traditional detoxification methods (for example, compatibility-based detoxification), formulation improvement (e.g., nanoencapsulation), and strategies such as personalized medicine/genetic screening and microbial metabolism. The center map outlines the mechanisms of mitochondrial damage, including ROS, mitochondrial membrane potential (ΔΨm) collapse, mtDNA damage, and mPTP opening.

#### 5.2.1 Natural products with favorable safety profiles

A variety of natural products have shown favorable safety profiles. Once their pharmacokinetic limitations are resolved, they may become suitable candidates for further development. Many studies have shown that gastrodin has a favorable safety profile: acute toxicity studies show that there is no mortality or serious adverse reactions even at high doses; long-term administration does not affect survival, development, or major organ function; and its main metabolites do not show obvious toxicity, further supporting its good systemic tolerance (Li R. et al., 2021; Gao H. et al., 2025). Resveratrol has been widely studied for its antioxidant, anti-inflammatory, anti-aging, and cardioprotective properties and is usually well tolerated at moderate doses. A single-dose, open-label study of 500 mg resveratrol administered under fasting conditions in healthy adults confirmed its safety, while higher doses (2.5–5 g) occasionally cause mild to moderate gastrointestinal symptoms, which highlights the need for individualized monitoring during long-term or high-dose use (Ren et al., 2017; Sergides et al., 2016).

ICGA showed low acute toxicity in Wistar rats and existing animal models, while chlorogenic acid (CQA) showed good long-term tolerance in both animals and humans. Although CQA can induce DNA double-strand breaks *in vitro*, bacterial assays show no mutagenicity, and no obvious neurotoxicity of ICGA has been observed. However, the potential sensitization risk of CQA still warrants further assessment (Behne et al., 2023). Salidroside is well tolerated in animals, with a high median lethal dose (LD_50_ = 5.63 ± 0.33 g/kg) and no significant toxicities during prolonged administration; genotoxicity has not been observed in standard *in vitro* or *in vivo* assays (Zhang et al., 2021; Chen et al., 2017). Baicalein and baicalin show selective cytotoxicity toward cancer cells, low toxicity to normal cells, and good tolerability in animal studies. In some models, they even exert hepatoprotective effects. Although there are no major reports of long-term toxicity, prolonged administration should still be approached cautiously, with attention to potential adverse effects (Hu et al., 2022).

Although toxicological data on chicoric acid remain limited, *in vitro* and animal studies indicate that, at conventional doses, it shows good tolerability together with antioxidant, anti-inflammatory, anti-HIV, anti-obesity, and insulin-sensitizing activities (Wang et al., 2016). Echinacoside demonstrates high safety in acute and long-term studies, without evidence of mutagenicity, carcinogenicity, or reproductive toxicity (Liu et al., 2018). Ferulic acid shows concentration-dependent anti-inflammatory effects in macrophage models (RAW 264.7), inhibits LPS-induced nitric oxide production at therapeutically relevant concentrations, and shows no cytotoxicity in the range of 10–100 μM (Yan et al., 2020). Under reasonable exposure conditions, rosmarinic acid has likewise shown good tolerability in single-dose studies as well as in repeated clinical and animal studies (Yehya et al., 2019; Nunes et al., 2015).

Long-term evaluations of dihydrochalcones indicate that NHDC lacks carcinogenicity, teratogenicity, or genotoxicity (including in a 15-year USDA program), and the European Union has established an acceptable daily intake (ADI) of 5 mg/kg. *In vitro*, most dihydrochalcone derivatives show low cytotoxicity at concentrations ≤400 μM, although B-ring substituents modulate risk, with halogen or hydroxyl groups conferring higher safety than methyl/methoxy substituents, which may accumulate as metabolites (Pérez Gutiérrez et al., 2019; Liu et al., 2023; Xiao et al., 2017). Ginsenoside 20(S)-Rh2 also shows an excellent safety profile, with no evidence of genotoxicity and selective toxicity toward cancer cells. It has a wide therapeutic window and thus supports consideration for long-term anticancer treatment strategies (Shi et al., 2013).

#### 5.2.2 Hepatotoxicity

Because the liver is a major site of metabolism and oxidative biotransformation, it is a common target organ for natural product toxicity. EGCG shows dose-dependent risk: high-dose intake (≥800 mg/day) is associated with hepatotoxicity, reflected by increased transaminases, as well as dizziness and anemia. Although conventional doses (150–400 mg/day) rarely cause serious adverse reactions, the therapeutic window of epigallocatechin-3-gallate (EGCG) is relatively narrow, especially in fasting or susceptible populations. Note that the potential hepatotoxicity of EGCG has attracted regulatory attention. Some regulatory agencies for dietary supplements have issued warnings that high doses are not recommended (Andreu Fernández et al., 2020; Hazimeh et al., 2023). Curcumin may cause mild and dose-related liver function abnormalities. In rat experiments, a single dose of 50 mg/kg led to an increase in alkaline phosphatase (ALP) levels; in clinical applications, a daily dose of 0.9–3.6 g is associated with increased levels of nausea, diarrhea, ALP, and lactate dehydrogenase (LDH). In addition, simultaneous exposure to other metabolites may change the risk: compared with the control group, simultaneous exposure to pyridoxamine increases total cholesterol and white blood cell counts, indicating a potential synergistic oxidative and immunomodulatory effect (Liu et al., 2022; Otuechere et al., 2014).

Sophora flavescens-derived alkaloids have shown dose-dependent hepatotoxicity in a variety of models. In primary hepatocytes, the half-maximal inhibitory concentration (IC_50_) of matrine is 5.3 mmol/L, while the IC_50_ of oxymatrine is 19 mmol/L. In the experimental model, increased levels of alanine aminotransferase (ALT), ALP, aspartate aminotransferase (AST), and malondialdehyde (MDA) were observed, accompanied by glutathione (GSH) depletion and apoptotic changes. In the zebrafish model, the acute median hepatotoxic concentration (IC_50_) was 5.3 mmol/L, whereas the median lethal concentration (LC_50_) was 0.41 mmol/L, accompanied by hepatocyte vacuolization and apoptosis. During intravenous administration, rapid infusion may cause nausea, dizziness, and pain at the injection site, which can usually be relieved by reducing the dose or reducing the infusion rate. Compared with matrine, oxymatrine has similar oral bioavailability but stronger hepatotoxicity and aquatic biotoxicity (You et al., 2020). Paeonol and pomegranate-derived metabolites are rapidly metabolized and cleared in the body, but at high doses, they may produce metabolites that disrupt hepatic iron homeostasis and damage kidney function. However, in breast cancer models, both substances can restore tight-junction integrity and reduce circulating diamine oxidase (DAO), D-lactic acid, and endotoxin levels, thereby improving tumor-related intestinal barrier dysfunction (Hu et al., 2020; Wang Y. H. et al., 2023). Even natural products that usually have favorable hepatic safety profiles may show conditional hepatotoxicity: high doses or prolonged exposure to rosmarinic acid may impose metabolic stress on the livers of susceptible individuals; ellagic acid is cytotoxic to bronchial epithelial cells at concentrations >5 μg/mL and may have systemic effects; and artemisinin derivatives may induce hepatic oxidative stress at high exposure levels through iron-dependent ROS generation (Yehya et al., 2019; Mancuso et al., 2020).

#### 5.2.3 Nephrotoxicity

Nephrotoxicity is usually related to the route and dose of administration. Caffeic acid phenethyl ester (CAPE) shows low acute toxicity (oral LD_50_ in rats = 8–40 g/kg) and favorable organ tolerability. Subchronic intraperitoneal dosing (10–30 nmol/kg for 4 weeks) did not produce hepatic or renal injury or behavioral abnormalities, and conferred renoprotection in cyclosporine A-, cisplatin-, and gentamicin-induced nephrotoxicity models by reducing BUN, creatinine, and tubular necrosis (Akyol et al., 2014). CAPE also shows selective cytotoxicity toward cancer cells at high doses and does not damage normal fibroblasts or oral epithelial cells (Ali et al., 2020).

The renal risk of quercetin depends on both route and dose: oral doses of ≤1 g/person/day appear safe, whereas high-dose intravenous administration (>945 mg/m^2^) may cause vomiting, transient hypertension, nephrotoxicity, and hypokalemia. Animal studies indicate that even when toxicity is extremely low at 3,000 mg/kg, biological activity is still observed at much lower exposure levels (Vafadar et al., 2020; Rayamajhi et al., 2013). Formulation strategies may further reduce the risks associated with intravenous administration.

#### 5.2.4 Cytotoxicity

Although natural product-induced cell death may be beneficial in oncology, it also poses risks to normal cells at high concentrations or in susceptible tissues. The toxicological profile of sanggenon C includes dose-dependent oral toxicity and higher *in vitro* cytotoxicity than its stereoisomers. In BALB/c mice, oral administration of MA60 extract (30–100 mg/kg) containing sanggenon C does not affect body weight, clinical indicators, or organ weight; at 300 mg/kg, the maximum tolerated dose is defined by > 10% body-weight loss by day 4, although no histopathological tissue damage is observed. In Calu-3 cells, the IC_50_ of sanggenon C, 47.8 μg/mL, is lower than that of sanggenon D, 120.5 μg/mL, which may be attributed to its high lipophilicity (logP = 3.48) and stronger membrane distribution capacity. Although existing evidence indicates that sanggenon C is the main active metabolite, synergistic metabolites in the extract matrix may also further enhance cytotoxicity (Schwarzinger et al., 2024; Langeder et al., 2023).

Tannic acid shows relatively low toxicity toward human bronchial epithelial cells: at a concentration of 5 μg/mL, it can inhibit the formation of benzo[a]pyrene (B[a]P) metabolite-DNA adducts; in the concentration range of 1.5–3.0 μg/mL, it can inhibit the growth of multiple cell lines through free radical-mediated apoptotic mechanisms (Teel et al., 1986). This highlights its dose-dependent dual characteristics: it exerts antioxidant and DNA-protective effects at low doses, whereas it promotes apoptosis and cytotoxicity at higher exposure levels, a common feature of many polyphenolic metabolites. High-dose EGCG (≥800 mg/day) is cytotoxic to cancer cells but is less selective for normal tissues, whereas CAPE maintains preferential cytotoxicity toward malignant cells. Its IC_50_ range (0.15–10.7 μM) supports its further development in oncology (Hazimeh et al., 2023; Ali et al., 2020).

#### 5.2.5 Reproductive toxicity

The impact on reproduction is an important safety consideration, especially in the case of longer periods of use. The impact of a variety of natural products on reproductive parameters is dose dependent. Ginsenoside Rg1 (a tetracyclic triterpenoid saponin purified from dried root and rhizome of *Panax ginseng* C.A.Mey.) can improve the reproductive parameters of animals, including the number/quality of sperm and embryo survival, but high doses may damage ovarian function and embryonic development. Under routine exposure, adverse reactions are usually mild and reversible (such as nausea and diarrhea), while long-term or high-dose use may lead to high blood pressure or hypoglycemia (Gao et al., 2017). Artemisinin derivatives have a wide therapeutic window and low toxicity toward normal cells; nevertheless, embryotoxicity has been reported during organogenesis in pregnant mice and in pregnancy-related contexts. Their iron-dependent ROS production may be partially offset by induction of antioxidant enzymes; for example, high doses of DHA increase ROS in certain cell types, and this effect can be alleviated by free-radical scavengers or iron chelators (Mancuso et al., 2020).

#### 5.2.6 Hematotoxicity

Most natural products have a relatively limited effect on the blood and hematopoietic system at therapeutic doses, but vigilance is nevertheless required. Luteolin shows high acute safety at therapeutic concentrations, and the risk of hemolysis is negligible. Long-term exposure in mice had no significant effect on growth indices, organ indices, or histopathological characteristics (Vongthip et al., 2024). Matrine shows low acute toxicity after intraperitoneal injection at doses of 25–150 mg/kg, with no lethality or weight loss; even plant extracts at doses up to 2000 mg/kg did not cause death. Although transient platelet inhibition may occur under high acute doses, hematological indicators and liver and kidney function are usually normal. Intraperitoneal injection for 14 consecutive days did not cause motor dysfunction, suggesting that its neuroprotective effects may be achieved by maintaining calcium homeostasis and regulating the GABAergic system (Alelign et al., 2020).

#### 5.2.7 Other toxicities

In addition to organ-specific toxicities, natural products may show many other adverse effects, often resulting from interactions with specific off-target proteins or cellular processes. Berberine shows dose-dependent multisystem toxicity, among which cardiotoxicity is caused by blockade of the hERG potassium channel. Its acute lethality varies depending on species and route of administration: oral LD_50_ values in mice range from 329 mg/kg to 713.58 mg/kg, intraperitoneal LD_50_ values in mice range from 23 mg/kg to 57.61 mg/kg, and oral LD_50_ in rats is > 1,000 mg/kg. High-dose administration can induce gastrointestinal irritation, such as vomiting and diarrhea, neuromuscular symptoms, and gastric mucosal injury. Subacute administration (7–21 days) can reduce spleen weight and CD4^+^ and CD8^+^ T cell counts (intraperitoneal, 10 mg/kg) and increase ALT/AST levels (oral, 150 mg/kg). Long-term exposure (>90 days) may have UVA-related phototoxicity and atherosclerotic effects (for example, intraperitoneal injection of 5 mg/kg can accelerate atherosclerosis in apoE−/− mice). Special risks include reproductive toxicity during pregnancy (the lowest observed adverse effect level (LOAEL) in rats = 531 mg/kg, associated with fetal weight loss), neonatal hyperbilirubinemia caused by bilirubin-albumin displacement in patients with G6PD deficiency, and clinically relevant hERG K^+^ channel blockade (intravenous IC_50_ = 3.1–80 μM), which can prolong the QT interval and induce torsades de pointes. Because of its cationic charge, berberine tends to accumulate in cardiac mitochondria, thereby aggravating cardiac risk. At high concentrations (>100 μM), berberine can induce ROS accumulation and mitochondrial apoptosis by inhibiting adenine nucleotide translocase and activating caspase-3/8/9, thereby causing DNA damage in normal cells; meanwhile, the IC_50_ values in cancer cell lines such as HepG2 are markedly lower (25.2–34.5 μM) (Rad et al., 2017). In general, hERG-mediated cardiotoxicity remains the major warning signal. With increasing dose and exposure duration, the toxicity spectrum broadens from gastrointestinal discomfort to liver injury, immunosuppression, phototoxicity, and reproductive toxicity. Therefore, dose monitoring is crucial in clinical application. Pregnant women and patients with G6PD deficiency should be treated with caution: attention should be paid to interactions with CYP inhibitors, and the benefit–risk balance should be carefully evaluated.

Natural products and approved small-molecule drugs follow two roughly orthogonal pharmacological paradigms: the structure–metabolism–exposure trajectory of natural products, which reflects scaffold-driven multi-metabolite, multipathway, and multi-organ distribution; and the targeted dose-monitoring paradigm of clinical drugs, which is based on single-agent administration, relatively linear kinetics, and more controllable exposure. One of the clearest distinctions between the two lies in first-pass metabolism and dose proportionality. The oral bioavailability of polyphenols such as curcumin and resveratrol is usually <10%; extensive first-pass metabolism or conversion into smaller phenolic metabolites (such as ferulic acid) markedly reduces exposure to the active parent metabolites. At extremely high intake (8 g/day), CYP and UGT pathways may become saturated, resulting in a sharp increase in parent-metabolite concentrations together with increased ALT/AST levels. In contrast, the oral bioavailability of metformin is 50–60%; its first-pass metabolism is extremely low, and it is excreted through the kidneys in its parent form. Plasma concentration increases linearly with the dose. Adjusting the dose according to the eGFR can reduce the risk of lactic acidosis in patients with renal insufficiency (Zhang et al., 2016). Idebenone has high lipophilicity and oral bioavailability (>90%) and can quickly penetrate the BBB, so it has a delivery advantage over resveratrol. Resveratrol usually requires a higher dose to achieve the central nervous system effect (Javadov et al., 2018).

The distribution characteristics further distinguish natural products from conventional drugs. Salidroside shows preferential accumulation in skeletal muscle and gonadal tissue, and its muscle concentration is approximately twice the plasma concentration, suggesting possible endocrine-related distribution risks during long-term administration. The quaternary ammonium-like cationic structure of berberine makes it positively charged, enabling it to target the negative potential of the mitochondrial membrane and promoting enrichment in mitochondria, especially myocardial mitochondria. During intravenous administration, hERG channel inhibition, with reported IC_50_ values as low as 3.1 μM, may lead to QT interval prolongation. In contrast, the cerebrospinal fluid/plasma concentration ratio of edaravone is approximately 0.3, with a small apparent volume of distribution and low accumulation tendency; these characteristics are consistent with its clinical profile of limited cardiotoxicity at recommended doses and brain exposure achieved through short-term intravenous infusion. In contrast, natural flavonoids usually require high doses or carrier systems to overcome poor blood–brain barrier permeability (Soejima-Kusunoki et al., 2022).

Biotransformation usually determines the pharmacological properties of natural products. For example, EGCG is converted into valerolactone derivatives under the action of the gut microbiota, and pomegranate ellagitannins are converted into urolithin A. Individuals carrying the NQO1 C609T mutation may have reduced detoxification ability for EGCG-derived quinone metabolites. When the daily dose exceeds 800 mg/day, ROS-mediated hepatotoxicity may occur. Artemisinin derivatives must be converted into dihydroartemisinin through hepatic and systemic metabolism to exert maximum antitumor activity, but excessive administration may lead to reticulocytopenia (Mancuso et al., 2020). In contrast, the biotransformation effect of metformin is not obvious, as it is cleared by the kidneys; its half-life can be extended from 5 h to 6 h to more than 20 h in renal insufficiency. Idebenone is also not metabolized by CYP enzymes, thus reducing individual differences caused by CYP polymorphism (Zhang et al., 2016; Javadov et al., 2018).

These comparisons reveal a broader principle: the toxicity of natural products is usually related to dose and genotype. For example, EGCG-related hepatotoxicity is associated with NQO1 gene mutations and high exposure under fasting conditions; high-dose intravenous quercetin can induce renal tubular damage through metal chelation and ROS amplification (Vafadar et al., 2020; Rayamajhi et al., 2013). In contrast, the clinical application of approved drugs benefits from therapeutic drug monitoring and dose adjustment strategies based on renal function.

For example, when the eGFR is < 30 mL·min^−1^, metformin is contraindicated to reduce the risk of lactic acidosis; idebenone shows good renal safety because of its rapid clearance and limited accumulation (Zhang et al., 2016; Soejima-Kusunoki et al., 2022). Reasonable drug-combination strategies may help bridge the gap between the two models. For example, the combined treatment of metformin and berberine may activate AMPK together, thus reducing the dose, which can reduce metformin-related gastrointestinal intolerance and reduce the risk of QT interval prolongation associated with berberine (Leblanc et al., 2020).

Overall, natural products have expanded treatment options through multipathway regulation and tissue targeting, but they are still limited by first-pass metabolism, metabolic differences between individuals, and accumulation-related toxicity. In contrast, traditional drugs have advantages in pharmacokinetic predictability, exposure monitoring, and safety management. It is feasible to organically combine these two models: nanodelivery platforms, such as curcumin liposomes; structure-based derivative optimization, such as artemisinin analogs; and reasonable natural product-drug combinations, such as the antioxidant synergy between citicoline and edaravone, can enhance natural product-mediated therapeutic effects and reduce toxicity, while retaining the accuracy and safety of traditional drugs, thus broadening the treatment window.

### 5.3 Risk management and mitigation strategies

Although natural products have long been considered “green” and inherently safe substances, modern toxicological research shows that their risks depend on dose, metabolic pathways, host genetic background, and specific mechanisms that affect mitochondrial dynamics (see Section 4 and Figure 11). Traditional principles such as “the dose makes the poison” are not enough to address real-world safety issues. For example, even within the upper dose limit of 6 g stipulated in the Chinese Pharmacopoeia, *Sophora flavescens* (Kushen)-containing botanical drugs can still lead to increased liver enzymes; for individuals with NQO1 polymorphism, the intake of approximately 800 mg of EGCG derived from green tea may increase the risk of acute liver injury. Therefore, the research focus must shift from simple dose restrictions to mechanism-based detoxification strategies. The mitohormetic effects of many natural products further highlight the necessity of this transformation, which requires a deeper understanding of the interaction between natural products and mitochondria. To this end, we advocate a framework covering molecular, cellular, and organismal levels that combines known toxicity risks (such as bioactivation of EGCG-derived quinone intermediates and hepatotoxicity associated with Kushen) with layered interventions (including traditional processing/formula improvement, nanoencapsulation, and genotype-based administration strategies), so as to promote the application of natural products from simple “effective delivery” to “precise and safe use.”

**FIGURE 11:**
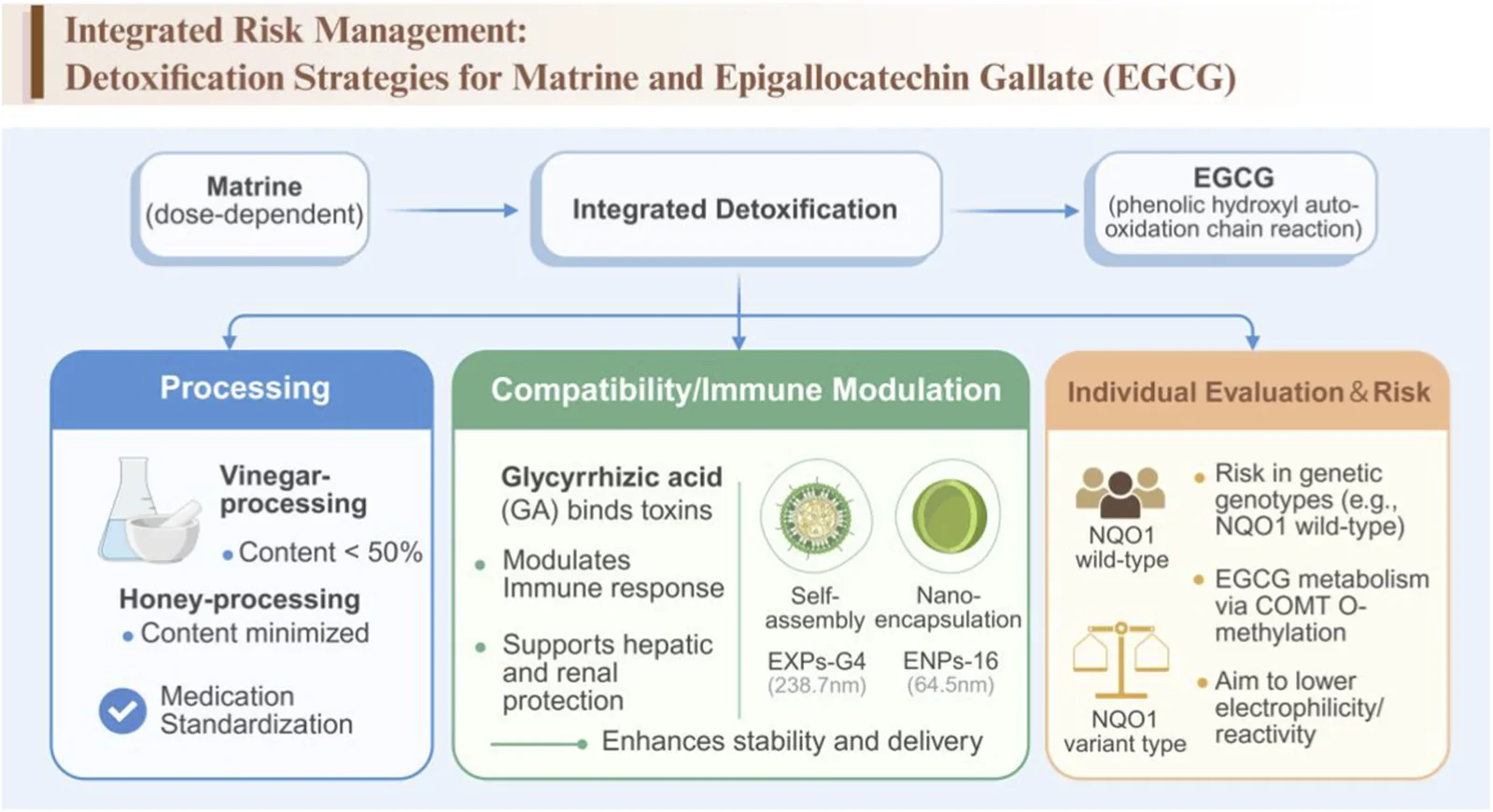
A proactive approach to safety: integrated risk management and detoxification strategies for matrine and EGCG. This figure illustrates integrated detoxification strategies for natural products, using matrine and EGCG as examples. For matrine (dose-dependent toxicity), risk management includes traditional processing methods (vinegar processing reduces content to <50%, and honey processing reduces it further), medication standardization/compatibility-based detoxification using glycyrrhizic acid (which binds toxins, modulates immune responses, and supports hepatic and renal protection), and strategies such as self-assembly encapsulation to enhance stability and delivery. For EGCG (phenolic hydroxyl auto-oxidation chain reactions), strategies focus on individual evaluation and risk assessment based on the recipient’s genetic genotype (e.g., NQO1 wild type vs. NQO1 variant carriers), with metabolism occurring via COMT-mediated O-methylation to reduce electrophilicity/reactivity. The diagram integrates processing, compatibility, and individualized evaluation to improve the safety profile of natural products.

Based on this framework, the detoxification of Kushen-containing botanical drugs depends on the dosage, processing method, and preparation design. A large retrospective cohort study (n = 382) showed that when the dose of the crude drug exceeded 10 g, the incidence of acute poisoning was as high as 46.3%, which emphasized the need to strictly follow the upper dose of 6 g stipulated in the Pharmacopoeia and adopt a detoxification strategy.

Traditional processing methods can significantly reduce the toxic load: vinegar processing can reduce toxicity by approximately 50%, while honey processing can further reduce toxicity. Compared with the stir-frying method, the soaking method retains a higher level of total alkaloids (1.07% vs. 0.41%), helping to maintain therapeutic activity while limiting peak exposure. Formulation design introduces additional regulatory mechanisms: glycyrrhizic acid can reduce liver damage by binding to toxic metabolites and regulating the immune response; bitter ginseng and licorice can be used in a ratio of 1:2 to effectively regulate oxidative stress indices in animal models (Zeng et al., 2025). Conversely, the combination with certain fermentation preparations may increase the solubility of alkaloids, causing dizziness, palpitations, and central nervous system toxicity. The actual drug administration specifications are also very important: avoiding co-administration with alcohol may prevent ethanol-induced CYP450 upregulation and metabolic bioactivation, and avoiding prolonged decoction can limit the oxidation of bitter ginseng to oxidized bitter ginseng (Zeng et al., 2025).

EGCG provides a complementary model, namely inhibiting oxidative self-toxicity through physicochemical restructuring and individualized metabolic management. Its phenolic hydroxyl groups can undergo auto-oxidation in weakly alkaline environments, triggering chain reactions that generate reactive quinone species and H_2_O_2_ (Pandey et al., 2020; Wang G. et al., 2023). In intestinal fluid, EGCG forms two types of nanoparticles, EGCG_16_ (64.5 nm) and EGCG_64_ (238.7 nm), through ionic and hydrophobic interactions. Nanoencapsulation reduces phenolic reactivity, thereby reducing H_2_O_2_ generation and quinone–protein adduct formation (Wang G. et al., 2023; Wu et al., 2024; Ushiki et al., 2024). Particle size is crucial: EGCG_64_ shows higher *in vivo* safety. At a dose of 80 mg/kg, it can activate the Nrf2 signaling pathway and induce expression of detoxification enzymes, including HO-1 and NQO1, and glutathione-synthesis enzymes, including GCLC and GSS. At low doses, it does not interfere with the basal antioxidant system, including TrxR/Grx, thereby avoiding rebound oxidative stress (Capasso et al., 2025; Ushiki et al., 2024; Wu et al., 2024). Individual differences are equally important: people with wild-type NQO1 can efficiently reduce EGCG-derived quinones to less reactive hydroquinone species, whereas individuals carrying the C609T variant may show impaired detoxification capacity and increased risk (Pandey et al., 2020; Hazimeh et al., 2023). Other detoxification pathways, including COMT-mediated O-methylation and glutathione conjugation, further support genotype-guided individualized dosing strategies.

Despite differences in strategy, the case studies of matrine-containing crude drugs and EGCG consistently clarify three principles for the safe use of natural products. First, it is necessary to clarify the molecular source of toxicity, such as whether it involves the oxidative metabolites of Kushen or the reactive intermediates of EGCG, in order to formulate targeted countermeasures. Second, traditional methods such as processing and classical formula compatibility should be combined with modern technologies such as nanoencapsulation and enzymatic pathway regulation to achieve multi-scale regulation of exposure levels and chemical reactivity. Third, precision intervention can be achieved through gene stratification and dynamic therapeutic windows based on dose-response models. Only by integrating these elements into development and clinical practice can efficacy and safety be harmonized.

Finally, pharmacokinetics and toxicology jointly define the dual threshold of clinical translation. Low bioavailability and metabolic instability not only weaken therapeutic efficacy (for example, human exposure to curcumin rarely reaches *in vitro* effective concentrations) but may also concentrate local toxicity (for example, EGCG-associated hepatotoxicity and paeonol metabolite-driven nephrotoxicity). In addition, species-specific PK differences (such as distinct curcumin metabolites in humans and rats) and disease-state dependence (for example, the prolonged half-life of echinacoside in Parkinson disease models) further complicate translational prediction. Overcoming these PK-toxicity bottlenecks requires both mechanism-level clarification and technical risk-mitigation strategies, which currently represent both the major challenge and the most promising direction for promoting the safe and effective clinical application of natural products.

## 6 Overcoming translational hurdles: future strategies for precision mitochondrial medicine

This review summarizes how natural products regulate mitochondrial dynamics, thereby providing mechanistic entry points for therapy. We integrate these functions into a four-dimensional regulatory framework (biogenesis, morphology, quality control, and defense). The main mechanisms of action include activation of the AMPK/SIRT1/PGC-1α axis (for example, EGCG, resveratrol, and ginsenoside Rg1) to drive mitochondrial biogenesis; recalibration of the fission–fusion balance (for example, curcumin attenuating DRP1 activity and matrine influencing OPA1 processing), which is usually mediated by MERCs; activation of mitophagy pathways (for example, 20(S)-Rh2 activating PINK1/Parkin and luteolin suppressing BNIP3L/NIX), which are facilitated by MLCs; and fortification of antioxidant defenses (for example, CAPE enhancing SOD/CAT activity and apigenin modulating Bcl-2/Bax signaling). These mechanisms work together to maintain mitochondrial network homeostasis and show potential application value in the treatment of neurodegenerative diseases, cardiovascular diseases, metabolic disorders, and cancer. However, problems such as mechanistic complexity, translational bottlenecks (especially PK/PD mismatch), and safety limitations urgently need to be systematically integrated and addressed.

### 6.1 Advances in natural product-mediated mitochondrial regulation: establishing a multidimensional framework

#### 6.1.1 Establishing a multidimensional synergistic framework

Extensive research has elucidated how natural products integrate mitochondrial biogenesis, morphology, quality control, and redox defense into a coordinated network. This review synthesizes these advances within a four-dimensional regulatory framework.

In terms of mitochondrial construction, namely biogenesis, activation of the AMPK/SIRT1/PGC-1α hub coordinates mitochondrial renewal. EGCG enhances PGC-1α activity through SIRT1-dependent deacetylation, thereby promoting mtDNA replication and respiratory chain assembly. Resveratrol can improve the NAD^+^/NADH ratio, upregulate MFN2, and improve energy efficiency. It has been reported to restore mitochondrial function in models of diabetic nephropathy and neurodegenerative disease. MFN2 not only regulates mitochondrial fusion but is also a key tethering protein for MERCs (Prudent and McBride, 2017). In terms of morphological regulation, particularly fission–fusion balance, the DRP1/OPA1/MFN1/2 axis maintains the integrity of the mitochondrial network (Lei et al., 2022). DRP1 SUMOylation can stabilize these contact sites and coordinate fission initiation and calcium-dependent cristae remodeling (Prudent and McBride, 2017). Curcumin inhibits the GTPase activity of DRP1 and activates the SIRT3/OPA1 fusion pathway, thus limiting mitochondrial fragmentation and stabilizing the cristae structure (Lei et al., 2022). Gastrodin upregulates MFN1/2 and OPA1 expression by promoting Nrf2 nuclear translocation, thereby restoring mitochondrial network continuity in ischemic stroke models.

In terms of quality control, namely mitophagy, mitochondrial dynamics are closely coupled with autophagy. Mitophagy enables selective removal through autophagosome nucleation in PI3P-enriched microdomains. Ginsenoside 20(S)-Rh2 enhances PINK1/Parkin-mediated ubiquitination and thereby removes damaged mitochondria. In addition, the proposed gut microbiota–mitochondria axis, for example, microbiota-derived urolithin A activating PINK1/Parkin-dependent mitophagy, helps overcome limitations imposed by first-pass metabolism, although this strategy must still be optimized according to individual metabotypes. The multiple effects of natural products give them a wide range of potential in the treatment of a variety of diseases. In neurodegenerative diseases, luteolin inhibits BNIP3L/NIX-driven excessive mitophagy and protects neurons from glutamate-induced excitotoxicity; quercetin enhances autophagy flux through SIRT1-PINK1 cascading, thus reducing Aβ-induced mitochondrial damage (Vongthip et al., 2024; Vafadar et al., 2020).

#### 6.1.2 Expansion of disease application scenarios

The multi-efficacy of natural products gives them therapeutic potential across multiple disease contexts. In neurodegenerative diseases, luteolin inhibits BNIP3L/NIX-driven excessive mitophagy and protects neurons from glutamate-induced excitotoxicity, whereas quercetin enhances autophagic flux through the SIRT1/PINK1 cascade, thereby attenuating Aβ-induced mitochondrial damage (Vongthip et al., 2024; Vafadar et al., 2020). In cardiovascular diseases, paeonol increases MFN2 expression through the PKCε/STAT3 axis, thereby ameliorating doxorubicin-induced cardiotoxicity; rosmarinic acid rebalances MFN1/2 and DRP1 expression, thereby reducing ischemia–reperfusion injury (Ding et al., 2023; Hu et al., 2020; Nunes et al., 2015). In metabolic disorders, berberine activates the AMPK/mTOR/ULK1 pathway to restore autophagy and reverse insulin resistance. Isochlorogenic acid A coordinates autophagy and fusion through the NRF2/LC3-II signaling pathway, thereby reducing lipotoxicity associated with non-alcoholic fatty liver disease (Feng et al., 2021; Lai et al., 2024). In cancer, artemisinin uses iron-dependent ROS to drive PINK1 accumulation and mitophagy in tumor cells; curcumin inhibits DRP1 in hepatocellular carcinoma, induces apoptosis in malignant cells, and promotes fusion in non-malignant hepatocytes, reflecting the bidirectional regulation imposed by dose and microenvironmental context (Lei et al., 2022; Mancuso et al., 2020; Zhu et al., 2022).

#### 6.1.3 Conceptual innovation and technological integration

We have outlined four conceptual advances to build a framework for future work. First, a four-dimensional regulatory framework integrating biogenesis, mitochondrial dynamics, quality control, and defense highlights natural product synergies; for example, rhein activates SIRT1/PGC-1α-driven biogenesis while regulating autophagy proteins to close a biogenesis–repair–clearance loop (Yin et al., 2022). Second, the gut microbiota–mitochondria axis explains the systemic role of microbiota-derived metabolites (such as urolithin A), provides a strategy to overcome first-pass limitations, and promotes personalized treatment based on metabotype stratification (Singh et al., 2022; Li R. et al., 2023; Kern et al., 2025).

Third, the hub-pathway integration model regards AMPK/SIRT1/PGC-1α as the core node connecting energy sensing, dynamic balance, and autophagy. For example, low-dose EGCG can simultaneously promote mitochondrial DNA replication and reduce the phosphorylation level of DRP1 at Ser616 (Valenti et al., 2013). Fourth, as the main principle underlying the actions of natural products, mitochondrial hormesis (mitohormesis) provides a key framework for understanding dose-dependent bidirectional effects and optimizing the therapeutic window.

Usually, the mitohormetic effect is triggered by the slight disturbance of mitochondrial electron transfer or redox balance caused by low-dose natural products, which constitutes a basic and unified mechanism of action. This mechanism explains why natural products with diverse structures and low systemic bioavailability can still induce beneficial adaptive reactions in the body, thus helping to bridge the gap between pharmacokinetics and pharmacodynamics. This represents an important conceptual shift: research should not only describe isolated mechanisms of action but should also focus on adaptive stress signals at low doses rather than direct target binding at high doses, which may be crucial for many natural products.

Despite the above progress, some urgent problems remain to be solved, such as unclear protein interaction networks, bidirectional effects depending on specific situations in the disease microenvironment, and limited bioavailability. Solving these problems requires high-content imaging, single-cell multi-omics, AI-guided network pharmacology, and formulation science. The next section will systematically discuss these challenges and propose technology-driven solutions.

### 6.2 Current challenges and mechanistic bottlenecks

#### 6.2.1 Complexity of mechanisms regulating mitochondrial dynamics

The study of how natural products regulate mitochondrial dynamics faces multiple intertwining challenges. Multi-level PTMs of core effector proteins make the balance between mitochondrial fission and fusion highly dynamic, and the quantitative threshold required for balance remodeling is still unclear. Key proteins such as DRP1, MFN1/2, and OPA1 are dynamically regulated by phosphorylation, SUMOylation, and ubiquitination (Gawlowski et al., 2012; Ma et al., 2020). For example, DRP1 integrates antagonistic phosphorylation events at the activation site Ser616 and the inhibitory site Ser637 (Chang et al., 2021). Kinase activity varies across different contexts, such as CDK1 during cell division and AMPK during energy stress, which leads to competitive PTM regulatory logic and functional plasticity of the system (Taguchi et al., 2007). In an Alzheimer disease model, EGCG reduces pathological mitochondrial fission by changing the phosphorylation balance between Ser616 and Ser637: the activation of AMPK not only inhibits CDK1-dependent Ser616 phosphorylation but also promotes Ser637 phosphorylation, thus inhibiting excessive mitochondrial fragmentation (Chu et al., 2024). OPA1 further increases this complexity; it is expressed in multiple splice variants and proteolytically processed forms, among which the stability or cleavage of the L-OPA1 is affected by changes in membrane potential and regulated by OMA1 and YME1L. The S-OPA1 mediates the remodeling of mitochondrial cristae and may partially compensate for impaired fission through a mechanism that has not been fully clarified (Javadov et al., 2018; Wang P. et al., 2024).

The spatial complexity of organelle communication is also a major challenge. Although MERC and MLC are considered important regulatory hubs, the specific mechanism by which natural products regulate the stability, composition, and signaling function of these contact sites is still unclear. At present, tools for visualizing and quantitatively analyzing these dynamic interactions after natural product treatment are still under development. The interaction between mitochondrial dynamics and mitophagy also involves a variety of protein and signaling pathway nodes. Some key issues have not been solved, limiting the development of targeted strategies and reasonable natural product design. In PINK1/Parkin-dependent mitophagy, the type and function of Parkin ubiquitin substrates (such as VDAC1 and MFN1/2) vary from tissue to tissue, and the role of specific ubiquitination-site topology has not been fully clarified (Pickles et al., 2018). Under hypoxia or metabolic stress, receptor-mediated parallel pathways (such as FUNDC1 and BNIP3L/NIX) will also be activated. A study on turmeric extract in Alzheimer disease shows that the activation of PINK1/Parkin can enhance the interaction between FUNDC1 and LC3 by promoting FUNDC1 phosphorylation; in contrast, impairment of FUNDC1-mediated mitophagy will aggravate Aβ deposition and neuronal apoptosis. These findings emphasize the cell-type specificity of signal integration (Qi et al., 2025). However, the mechanistic interface between receptor-mediated mitophagy and the PINK1/Parkin axis remains incompletely understood (Wang H. et al., 2023; Pickles et al., 2018).

#### 6.2.2 Bottlenecks in natural product clinical translation

As mentioned in Section 5, the therapeutic effects of natural products are often limited by pharmacokinetic barriers such as low oral bioavailability, rapid clearance, and metabolic instability. To overcome these liabilities, strategies such as nanodelivery systems (for example, liposomes and pH-responsive micelles) and structure-guided modifications have been adopted (Wang et al., 2018; Mani et al., 2021). In rabbits, liposomal curcumin follows a two-compartment model: plasma exposure increases by about 3–5-fold, and the half-life is extended to 8–12 h, characterized by rapid distribution and slow elimination. Even so, the antiproliferative activity of curcumin at 10–40 μM *in vitro* is difficult to reproduce *in vivo* without co-formulation or combination therapy. Therefore, it has been proposed that curcumin be combined with metabolites such as resveratrol or ferulic acid to increase exposure and augment efficacy. Without resolving this exposure gap, mechanistic findings remain largely of academic interest.

The polypharmacological nature underlying the broad therapeutic effects of natural products simultaneously complicates their clinical translation. Natural products usually regulate mitochondrial dynamics through multipathway synergy, which can blur primary and secondary targets and increase the difficulty of target deconvolution (Xie et al., 2020; Bonelli et al., 2019). In addition, the direction of the effect may depend on the tissue context or pathological microenvironment, as exemplified by the principle of mitohormesis. Another major confounding factor, especially for orally administered natural products, is the gut microbiota. Its role in biotransformation introduces substantial interindividual pharmacokinetic and pharmacodynamic variability. Clinical trials that do not account for participants’ metabotypes, for example, by stratifying urolithin producers and non-producers when testing pomegranate extracts, effectively pool data from subgroups receiving different active treatments and different effective doses, thereby greatly complicating interpretation of efficacy and safety outcomes. Single-cell transcriptomic studies also show that curcumin can bidirectionally regulate DRP1: in hepatocellular carcinoma, it inhibits DRP1 Ser616 phosphorylation, thereby suppressing mitochondrial fission and inducing apoptosis; in normal hepatocytes, curcumin activates AMPK and promotes fusion, thereby maintaining liver function.

Further barriers to development include the lack of high-throughput *in vivo* screening platforms, limited large-scale clinical datasets, constraints of species-specific models, and physiological compensation that may mask drug effects. To bridge the gap in these models, organoid systems have begun to link the results of preclinical research with translational applications: in human brain organoids that simulate the microenvironment of amyloid plaques, quercetin activates PINK1/Parkin-dependent mitophagy and significantly reduces neuronal mitochondrial fragmentation (Gorgey et al., 2023) In short, sustained progress requires integration of drug-delivery science aimed at enhancing exposure, mechanism-based target identification, and next-generation disease models, so as to accelerate translation of mitochondrial regulation into clinical benefit. Crucially, future clinical trial designs must also take into account microbiota-mediated metabolic heterogeneity, for example, through prescreening, stratification, or direct supplementation of active metabolites.

#### 6.2.3 Technological innovations driving future research

The combination of artificial intelligence (AI) and machine learning with single-cell dynamic imaging and genome-scale perturbation platforms is redefining the evaluation of natural products in mitochondrial biology. Tools such as mitochondrial GTP/GDP fluorescent sensors, for example, GIC-GTP/GDP or related genetically encoded sensors, can analyze natural product effects in spatiotemporal dimensions and specific cellular states in living systems. In parallel, CRISPR interference (CRISPRi) combined with multi-omics and spatial mass spectrometry imaging enables rigorous target validation and mechanistic analysis, for example, visualization of tanshinone IIA accumulation in cardiomyocyte mitochondria (Zou et al., 2025).

Crucially, the application of AI should not be limited to current roles in mechanistic analysis and target identification. A logical and necessary next step is to use it as a generative engine for the rational design of synergistic multicomponent therapies. Based on multi-omic data, including transcriptomics, proteomics, and metabolomics, and AI models trained on high-content phenotypic screening data from single metabolites and combinations, researchers can predict the optimal metabolite composition and ratio of purified natural products. The strategy aims to achieve multi-target effects on mitochondrial networks, such as simultaneously enhancing mitochondrial biogenesis, fusion, and mitophagy, while minimizing off-target toxicity by ensuring that the concentration of each metabolite remains below its respective toxicity threshold and by accounting for its unique pharmacokinetic characteristics. Together, these technologies have established a complete workflow from discovery to verification and are expected to accelerate the translational research of natural product-mediated mitochondrial regulation.

#### 6.2.4 High-throughput screening and precision mechanism elucidation technologies

High-throughput screening (HTS) can quickly analyze a large number of natural products and their derivatives, so as to identify regulators of mitochondrial dynamics. A typical example is the discovery of NAT-5r, which is an activator of nicotinamide phosphoribosyltransferase (NAMPT); this metabolite promotes NAD^+^ biosynthesis, corrects mitochondrial energy metabolism defects, and shows therapeutic effects in disease models of neurodegenerative diseases characterized by mitochondrial dysfunction (Wang et al., 2022). When combined with advanced cell systems and biosensors, HTS can provide higher-fidelity readouts: multiplexed detection technologies can monitor mitochondrial membrane potential, reactive oxygen species (ROS) levels, and fusion–fission events in real time, thus quantifying the impact of natural products on mitochondrial dynamic network behavior (Sáez-Calvo et al., 2017).

Genetically encoded biosensors further improve the resolution of mechanistic analysis. The mitochondrial GTP/GDP sensor GRISER-HR is a combination of a circularly permuted yellow fluorescent protein (cpYFP) and a guanine nucleotide binding module, which can track the mitochondrial metabolic status in real time through changes in the GTP/GDP ratio, thereby helping to evaluate the effects of natural products on mitochondrial energy homeostasis (Johnson et al., 2020; Zhang et al., 2022). By integrating such reporter constructs into the high-throughput screening (HTS) platform, the spatial and temporal effects of natural products on membrane potential, ROS flux, and fusion–fission balance can be accurately evaluated in parallel, thus improving screening efficiency and the accuracy of mechanistic analysis.

The genome editing technology based on CRISPR provides a strong complement to the above methods by establishing causality. By targeting or knocking out/knocking in mitochondrial dynamics-related genes, such as DRP1 and MFN1, researchers can deeply analyze the impact of natural products on the morphology and function of organelles (Duan et al., 2020). In addition, the combination of CRISPRi and single-cell transcriptomics helps to analyze the polypharmacology of natural products: by systematically silencing candidate genes, researchers can identify the key nodes that mediate the effect of natural products. In studies of ischemic stroke, ginsenoside Rg1 can simultaneously regulate OPA1, mitophagy receptor FUNDC1, and antioxidant enzyme SOD2, thus constructing a coordinated protection network. CRISPRi-mediated OPA1/FUNDC1 silencing experiments help to verify whether the efficacy of Rg1 depends on these targets (Gelléri et al., 2023). Therefore, disease-related genome editing and single-cell readout technologies provide a physiologically relevant platform for evaluating the therapeutic potential of natural products.

#### 6.2.5 Innovations in metabolism and delivery technologies

Mass spectrometry imaging (MSI) can intuitively visualize the distribution and metabolism of natural products in tissues and cells, thus clarifying their mitochondrial entry pathways, subcellular localization, and molecular interactions (Minelli et al., 2010). This technology is crucial to confirm whether natural products reach mitochondrial targets *in vivo* and helps to solve the key problem of pharmacokinetics/pharmacodynamics (PK/PD) mismatch. Li and colleagues used spatial mass spectrometry imaging to confirm that tanshinone IIA can be selectively enriched in mitochondria, and its abundance is positively correlated with the integrity of mitochondrial cristae. Complementary fluorescence-probe studies show that tanshinone IIA can improve mitochondrial membrane potential (ΔΨm) and inhibit the accumulation of ROS, thus alleviating mitochondrial dysfunction caused by ischemia–reperfusion (Zou et al., 2025). In addition to providing static snapshots, MSI can also track the generation and turnover of metabolites *in vivo*, providing key data for dose optimization, efficacy evaluation, and safety analysis.

The fluorescent probes targeting mitochondria can monitor the dynamic changes of membrane potential, ROS, and calcium ions (Ca^2+^) in real time, thus improving the sensitivity and specificity of analysis (Johnson et al., 2020). When combined with advanced microscopy technologies, including confocal and super-resolution imaging, these probes can achieve high-resolution imaging of mitochondrial network remodeling, cristae structure, and cytoskeletal interaction. For example, super-resolution studies show that quercetin can trigger the rapid reorganization of mitochondrial networks and regulate the cristae structure by inducing OPA1 processing (Gao et al., 2023). Similarly, using a probe to monitor the effect of ginsenoside Rg1 on membrane potential can provide actionable data on bioenergetic regulation.

Multi-omics analyses, including genomics (Zhong et al., 2021), transcriptomics, proteomics (Bantscheff et al., 2007; Savitski et al., 2014), and metabolomics, can reconstruct the mitochondrial dynamics network regulated by natural products at the system level. By integrating natural product-induced expression profiles, protein–protein interactions, post-translational modifications, and metabolite changes, these methods can construct a comprehensive mechanism model (Savitski et al., 2014). Taking berberine as an example, multi-omic analysis can reveal the synergistic regulation between mitochondrial metabolic enzymes and respiratory chain complex proteins. At the same time, acetylation and other modifications may affect its functional effect. Importantly, these datasets can also reveal biomarkers associated with mitochondrial dysfunction, thus supporting early diagnosis, efficacy monitoring, and pharmacokinetic evaluation. Specifically, metabolomic indicators help to quantify the intervention effect of natural products in different disease contexts.

### 6.3 Future directions: multi-target precision medicine and clinical translation

The emerging platforms discussed above constitute a comprehensive toolkit for overcoming current limitations and guiding future research directions. Looking to the future, research and development should focus on clinically oriented multi-target precision regulation and interdisciplinary technological innovation to closely integrate mechanism research with translational medicine.

#### 6.3.1 Research on multi-target precision regulation

Future investigations should transition from unidimensional interventions to precise multi-node network regulation of mitochondrial dynamics. This means that research should go beyond the identification of a single target and instead explore how natural products regulate the entire regulatory network, especially the regulatory effect of inter-organelle contact sites. Existing studies have confirmed this trend. For example, ginsenoside Rg1 can maintain mitochondrial morphology and bioenergetic metabolism and may improve dysfunction associated with AD through multiple mechanisms; this indicates that effective intervention strategies may coordinate mitochondrial biogenesis, dynamics, mitophagy, and redox defense. Quercetin protects H9C2 myocardial cells by upregulating the long non-coding RNA PVT1; therefore, it warrants systematic evaluation in different cell types and disease backgrounds to elucidate its multi-target mechanism. Similarly, the antioxidant and metabolic effects of resveratrol extend beyond neurodegenerative diseases to include metabolic syndromes and age-related disorders, whereas the neuroprotective effects of luteolin against glutamate-induced oxidative stress and autophagy-mediated neuronal death support its evaluation in additional neurodegenerative disease models.

Mitochondrial quality control will remain a central theme, encompassing biogenesis, fission–fusion dynamics, mitophagy, and oxidative stress buffering. For example, baicalein enhances Nrf2 nuclear translocation, thereby recalibrating mitochondrial dynamics and maintaining mitochondrial structure and function, thus protecting H9C2 cells from oxidative damage. A key goal is to clarify how natural products exploit integrated quality control programs, rather than isolated nodes, to prevent and treat disease. In addition, future research must prioritize identification of the optimal dose range of key natural products and move beyond existing study designs centered on the maximum tolerated dose (MTD). At the same time, formulation science should be emphasized as crucial for improving bioavailability and chemical stability, thereby enhancing therapeutic efficacy (Liu et al., 2025). Interdisciplinary strategies combining cell and molecular biology, bioinformatics, and clinical methods will accelerate mechanistic analysis and candidate-target prioritization; for example, network pharmacology can be used to explore how ginsenoside Rg1 regulates ferroptosis in Alzheimer disease and to identify experimentally testable targets. These methods will help transform multi-target effects into precise and flexible therapeutic strategies.

#### 6.3.2 Clinical translation and cross-disciplinary technological innovation

From mechanistic exploration to clinical benefit, the development of natural products requires systematic integration. In the discovery stage, rigorous basic research should be carried out, and technologies such as gene knockout/editing and proteomics should be used to analyze targets and signaling pathways to clarify the causal mechanisms and druggable nodes. Disease modeling must go beyond reductionist models and move toward scenarios that contain multiple factors and can better reflect the pathophysiological characteristics in order to more accurately predict therapeutic effects. The transition from preclinical to clinical research should be guided by biomarker design and PK/PD alignment, and PK/PD mismatches should be explicitly addressed through advanced delivery systems or through a focus on microbiota-derived metabolites. Subsequently, well-powered multicenter clinical trials should be carried out across indications such as neurodegenerative diseases, cardiovascular diseases, cancer, and metabolic disorders to verify broad applicability and safety. To this end, the wider use of single-cell sequencing, CRISPR–Cas9 gene editing, and AI-driven predictive modeling will improve resolution and throughput, thereby enabling more effective identification of lead natural products and their mechanisms of action (Hu et al., 2023). Finally, continuous investment in interdisciplinary cooperation will accelerate the translation of natural product-based mitochondrial interventions into evidence-based therapies.

## 7 Conclusion

Natural products are not single-target drugs but potential regulators of complex mitochondrial dynamic networks. This review constructs a four-dimensional regulation framework integrating biogenesis, mitochondrial dynamics, quality control, and oxidative defense, thus revealing the synergy and multi-target characteristics of natural products. Crucially, the efficacy of natural products shows context-dependent and dose-responsive bidirectional regulation, that is, mitohormesis. For example, the different effects of curcumin on malignant and non-malignant hepatocytes and the regulatory effect of resveratrol on the AMPK/SIRT1/PGC-1α pathway highlight the regulatory potential of this context dependence. This review further emphasizes the role of inter-organelle communication sites, especially MERCs and MLCs, which serve as central platforms at which natural products recalibrate signaling microdomains and influence fission, fusion, calcium signaling, and mitophagy, thereby integrating these processes into coordinated cellular responses. Despite this potential, complex mechanistic interactions and significant pharmacokinetic barriers, especially low bioavailability (for example, curcumin <1%), continue to limit clinical translation, leading to a pronounced PK/PD disconnect.

To overcome these bottlenecks, we propose a paradigm shift that emphasizes the gut microbiota–mitochondria axis (for example, the generation of urolithin A) together with an integrated hub-pathway model, thereby providing a new strategy for optimizing systemic efficacy and individualized treatment through metabotype-informed approaches. In addition, given that mitohormesis may represent a principal mechanism underlying the effects of low-bioavailability natural products, future research should focus on defining the optimal hormetic dose window, rather than merely pursuing maximal exposure. The future of mitochondria-targeted therapy will depend on technological integration. The combination of AI-driven high-throughput screening and mechanism deconvolution, CRISPR-based target validation, and advanced mitochondria-targeted nanodelivery systems will be crucial for overcoming pharmacokinetic limitations and improving target specificity. By integrating multidimensional mechanistic insight with precision medicine strategies, natural products are expected to evolve from promising theoretical entities into transformative personalized therapies for diseases associated with mitochondrial dysfunction.

## Glossary

AD: Alzheimer disease
AI: Artificial intelligence
AUC: Area under the curve
ALT: Alanine aminotransferase
ALD: Alcoholic liver disease
AMPK: AMP-activated protein kinase
ANT: Adenine nucleotide translocator
ARE: Antioxidant response element
AST: Aspartate aminotransferase
ATP: Adenosine triphosphate
Aβ: β-Amyloid
BNIP3: Bcl-2/adenovirus E1B 19 kDa-interacting protein 3
BUN: Blood urea nitrogen
Bax: Bcl-2-associated X protein
Bak: Bcl-2 homologous antagonist/killer
Bcl-2: B-cell lymphoma 2
Bcl-xL: B-cell lymphoma-extra large
BH3: Bcl-2 homology domain 3
Bim: Bcl-2 interacting mediator of cell death
CA: Chicoric acid
CAT: Catalase
CDK5: Cyclin-dependent kinase 5
CHF: Chronic heart failure
CL: Clearance
COX: Cytochrome c oxidase
CRISPR: Clustered regularly interspaced short palindromic repeats
CRISPRi: CRISPR interference
CVDs: Cardiovascular diseases
CYP: Cytochrome P450
Cmax: Maximum concentration
DDC: Dihydrochalcone
DBIL: Direct bilirubin
DRP1: Dynamin-related protein 1
DNA: Deoxyribonucleic acid
ECH: Echinacoside
EGCG: Epigallocatechin gallate
eGFR: Estimated glomerular filtration rate
Epac1: Exchange protein directly activated by cAMP 1
ERK: Extracellular signal-regulated kinase
ETC: Electron transport chain
E3: Ubiquitin-protein ligase E3
ER: Endoplasmic reticulum
INF2: Inverted formin 2
IP3Rs: Inositol 1,4,5-trisphosphate receptors
G6PD: Glucose-6-phosphate dehydrogenase
GAS: Gastrodin
GDP: Guanosine diphosphate
GLUT4: Glucose transporter type 4
GPX: Glutathione peroxidase
GST: Glutathione S-transferase
GTP: Guanosine triphosphate
GSH: Glutathione
GS: Ginsenoside
HD: Huntington disease
HF: Heart failure
HIF-1α: Hypoxia-inducible factor-1 alpha
HO-1: Heme Oxygenase-1
IC50: Half-maximal inhibitory concentration
ICGA: Isochlorogenic acid A
IDH2: Isocitrate dehydrogenase 2
I/R: Ischemia/reperfusion
IMM: Inner mitochondrial membrane
IP: Intraperitoneal
IV: Intravenous
KLF4: Krüppel-like factor 4
LC3: Microtubule-associated protein 1A/1B-light chain 3
LD50: Lethal dose 50%
LIR: LC3-interacting region
LOAEL: Lowest observed adverse effect level
L-OPA1: Long-form OPA1
MAFLD: Metabolic-associated fatty liver disease
MAPK: Mitogen-activated protein kinase
MAPL: Mitochondrial-anchored protein ligase
MARCH5: Mitochondrial E3 ubiquitin-protein ligase
Mcl-1: Myeloid cell leukemia sequence 1
MCF-7: Michigan Cancer Foundation-7
MDA-MB-231: M.D. Anderson-metastatic breast-231
MFN2: Mitofusin 2
MFN1: Mitofusin 1
MFF: Mitochondrial fission factor
MiD49/51: Mitochondrial dynamics protein 49/51
MERCs: Mitochondria–endoplasmic reticulum contact sites
mHTT: Mutant Huntingtin protein
MLCs: Mitochondria–lysosome contacts
mPTP: Mitochondrial permeability transition pore
mTOR: Mammalian target of rapamycin
MQC: Mitochondrial quality control
MS: Mass spectrometry
MS/MS: Tandem mass spectrometry
MRT: Mean residence time
MTD: Maximum tolerated dose
NAD+: Nicotinamide adenine dinucleotide
NADH: Reduced nicotinamide adenine dinucleotide
NADPH: Nicotinamide adenine dinucleotide phosphate
NAFLD: Non-alcoholic fatty liver disease
NDP52: Nuclear dot protein 52
NDs: Neurodegenerative diseases
NOX4: NADPH oxidase 4
NQO1: NAD(P)H quinone dehydrogenase 1
NRF2: Nuclear factor erythroid 2-related factor 2
O-GlcNAcylation: O-Linked N-acetylglucosamine modification
OMA1: OMA1 zinc metallopeptidase
OMM: Outer mitochondrial membrane
OPA1: Optic atrophy 1
OPTN: Optineurin
OXPHOS: Oxidative phosphorylation
PG: Punicalagin
PGC-1α: Peroxisome proliferator-activated receptor gamma coactivator 1-alpha
PINK1: PTEN-induced putative kinase 1
PK: Pharmacokinetic
PK/PD: Pharmacokinetic/pharmacodynamic
PD: Parkinson disease
Papp: Apparent permeability coefficient
PTMs: Post-translational modifications
PI3P: Phosphatidylinositol 3-phosphate
RA: Rosmarinic acid
Rap1: Ras-related protein 1
RET: Reverse electron transport
Rg1: Ginsenoside Rg1
Rh2: Ginsenoside 20(S)-Rh2
RNA: Ribonucleic acid
ROS: Reactive oxygen species
SCs: Respiratory supercomplexes
SIRT1: Sirtuin 1
SIRT3: Sirtuin 3
SOD: Superoxide dismutase
S-OPA1: Short-form OPA1
SUMOylation: Small ubiquitin-like modifier modification
S-nitrosylation: S-nitrosylation
T2D: Type 2 diabetes
TMAO: Trimethylamine N-oxide
Tmax: Time to reach maximum concentration
TBIL: Total bilirubin
TF: Transcription factor
TFAM: Mitochondrial transcription factor A
TFEB: Transcription factor EB
T1/2: Half-life
SERCA: Sarcoplasmic/endoplasmic reticulum calcium ATPase
UHPLC: Ultra-high-performance liquid chromatography
ULK1: Unc-51-like autophagy activating kinase 1
**V**_**d**_: Volume of distribution
VDAC1: Voltage-dependent anion channel 1
