# Supplementary material for: Natural products targeting mitochondrial homeostasis: a four-dimensional framework integrating dynamics, mitophagy, pharmacokinetics, and translational safety
Source: Front Pharmacol. 2026 Sep 18;17:1911495. doi: 10.3389/fphar.2026.1911495 (PMC13670514; doi:10.3389/fphar.2026.1911495)
Supplement: Supplementary file 1 [file Table1.pdf]

Supplementary Table 1

Mechanisms of Action of Natural Products in Intervening in Mitochondrial Function via the Four-Dimensional Regulatory Framework

Regulatory Dimension	Core Mechanism	Representative Metabolite	Key Target/ Pathway	Biological Effect	Interaction & Synergy	Concentration	Disease Application	References	Evidence Classification	Mitophagy Evidence Tier
Foundational Construction (Biogenesis)	Activation of mitochondrial biogenesis via the AMPK/SIRT1-PGC-1α axis	EGCG	SIRT1-PGC-1α axis	Increase in mtDNA replication and an improvement in OXPHOS efficiency	Synergy with Nrf2 for enhanced antioxidant enzyme expression; biogenesis supplies the "raw material" for morphological remodeling and quality control	5 μM (Down syndrome cell models)	Neurodegenerative diseases, metabolic syndrome	(Valenti et al. 2013)	Class II Extreme PAINS; plasma nanomolar levels far below in vitro effective dose.	Tier II: Context-dependent putative mitophagy regulator
		Resveratrol	AMPK/SIRT1-PGC-1α axis; upregulation of the	Upregulation of MFN2, promoting	Activation of upstream energy sensors (AMPK) and	10-50 μM (podocyte and neuronal models)	Diabetic nephropathy, obesity-related	(Kapetanovic et al. 2010; Xie et al.	Class II High PAINS; conjugated metabolites	Tier II: Context-dependent putative

Regulatory Dimension	Core Mechanism	Representative Metabolite	Key Target/ Pathway	Biological Effect	Interaction & Synergy	Concentration	Disease Application	References	Evidence Classification	Mitophagy Evidence Tier
			NAD^+/NADH ratio	fusion and elongation	redox integrators (SIRT1), converging on PGC-1α		metabolic syndrome	2023; Zhang et al. 2019)	dominate human blood, inconsistent clinical results.	mitophagy regulator
		Ginsenoside Rg1	Upregulation of COX, TFAM	Increase in mitochondrial membrane potential and promotion of ATP synthesis	Enhancement of mitochondrial bioenergetics alongside biogenesis; metabolites (e.g., PPT) may extend pharmacodynamic persistence	10-20 μM (neuroprotection models)	Neuroprotection (upregulates respiratory chain metabolites)	(Kwan et al. 2021，Gao et al., 2017)	Class III<br>Low PAINS; P-gp efflux limits oral absorption, no human RCTs.	Tier III: No direct mitophagy activity evidence
		Berberine	SIRT1/PGC-1α axis; Inhibition of Complex I;	Raised AMP/ATP ratio and promotion of mitochond	Potent AMPK activation with context-dependent mechanisms (Complex I	1 μM (hippocampal neuron models against Aβ	Insulin resistance, non-alcoholic fatty liver disease	(Zhou et al. 2020)	Class III<br>Low PAINS; hERG cardiotoxic risk, scarce	Tier I: Well-validated selective mitophagy modulator

Regulatory Dimension	Core Mechanism	Representative Metabolite	Key Target/ Pathway	Biological Effect	Interaction & Synergy	Concentration	Disease Application	References	Evidence Classification	Mitophagy Evidence Tier
			Activation of AMPK	rial neogenesis	inhibition vs. LKB1 activation); a narrow therapeutic window				neurological human trials.	
		Echinacoside	Activation of AMPK	Significant increase in mitochondrial number and functional improvement	Extremely low oral bioavailability (~ 0.83%) limits translational relevance despite *in vitro* efficacy	50-100 μM (aging models)	Delay aging	(Zhou et al. 2020；Liu et al., 2018)	Class II<br>Medium PAINS; barely crosses intact blood-brain barrier orally.	Tier II: Context-dependent putative mitophagy regulator
	Activation of mitochondrial biogenesis; Regulation of autophagy-	Rhein	SIRT1/PGC-1α axis; Autophagy-related proteins	Maintenance of mitochondrial quality, forming a dynamic quality	Establishment of a closed-loop "generation-repair-clearance" paradigm by coupling	10-20 μM (hyperglycemia /lipotoxicity models)	Mitochondrial dysfunction induced by high glucose, high fat, oxidative stress	(Yin et al. 2022)	Class II<br>Medium PAINS; organ accumulation risks, limited lipid-toxicity models.	Tier III: Biogenesis regulator (no direct mitophagy evidence)

Regulatory Dimension	Core Mechanism	Representative Metabolite	Key Target/ Pathway	Biological Effect	Interaction & Synergy	Concentration	Disease Application	References	Evidence Classification	Mitophagy Evidence Tier
	related proteins to maintain mitochondrial quality			control system	biogenesis with autophagy regulation					
Morphological Regulation (Fission/Fusion Balance)	Balancing of fission/fusion by targeting the DRP1-OPA1/MFN1/2 axis	Curcumin	Inhibition of DRP1 GTPase activity; Activation of SIRT3-OPA1	Reduction of fragmentation and stabilization of cristae structure	Remodeling reduces formation of damaged mitochondria, lowering quality control load; synergy with antioxidant defense stabilizes membrane potential; context-dependent effects (pro-	5-50 μM (ALD/NAFLD cell models)	Alcoholic/non-alcoholic fatty liver disease	(Hasan et al. 2019; Ryu et al. 2024; Xu et al. 2013; Khatoon and Kalam, 2025)	Class II<br>High PAINS; ultra-low oral bioavailability, no mitochondrial human trials.	Tier II: Context-dependent mitophagy-associated regulator

Regulatory Dimension	Core Mechanism	Representative Metabolite	Key Target/ Pathway	Biological Effect	Interaction & Synergy	Concentration	Disease Application	References	Evidence Classification	Mitophagy Evidence Tier
					apoptotic in cancer)					
		Paeonol	Balancing of fission/fusion via the PKCε-Stat3 pathway leading to upregulation of MFN2	Promotion of mitochondrial fusion	Action via transcription factor-driven programs for sustained effects	10-50 μM (cardiomyocyte models)	Doxorubicin-induced cardiotoxicity, cardiomyocyte protection	(Ding et al. 2023；Hu et al., 2020)	Class III<br>Moderate PAINS; Low oral bioavailability, only cardiac/renal animal data.	Tier III: No direct mitophagy activity evidence
		Rosmarinic Acid (RA)<br>Chicoric Acid (CA)	Balancing of the protein ratio of Mfn1/2 and DRP1	Further balancing of mitochondrial fission/fusion	Direct protein normalization; complementary action to transcriptional approaches	RA: 10-100 μM (cardiovascular models)<br>CA: 50 mg/kg (diabetic rat models)	Cardiomyocyte protection	(Ding et al. 2022; Gupta et al. 2022；Wang et al., 2016)	Class II<br>High PAINS; severe first-pass clearance, no human mitochondrial data.	Tier III: No direct mitophagy activity evidence
		Isochlorogenic Acid A (ICGA)	Downregulation of DRP1 expression	Improvement of mitochondrial	Coordination of antioxidant signaling (Nrf2) with	64 μg/mL (macrophage models against mycotoxins)	Non-alcoholic fatty liver disease, synaptic	(Gray et al. 2014; Lai et al. 2024;	Class III<br>Moderate PAINS; Poor oral	Tier III: Mitochondrial dynamic/red

Regulatory Dimension	Core Mechanism	Representative Metabolite	Key Target/ Pathway	Biological Effect	Interaction & Synergy	Concentration	Disease Application	References	Evidence Classification	Mitophagy Evidence Tier
			and upregulation of MFN2/OPA1	network continuity	fusion promotion (MFN2/OPA1) for synergistic amplification		protection in neurodegeneration	Duan et al. 2024)	permeability, liver/neuro animal models only.	ox modulator (no mitophagy evidence)
		Quercetin	Promotion of PVT1 expression	Inhibition of mitochondrial fission and promotion of mitochondrial fusion	Induction of lncRNA PVT1, potentially acting through non-phosphorylation mechanisms (e.g., ubiquitination)	5-20 μM (inflammatory models)	Mitochondrial inflammatory damage	(F Li et al. 2021; Vafadar et al. 2020)	Class II<br>Extreme PAINS; low oral uptake, weak blood-brain tissue penetration.	Tier I: Well-validated selective mitophagy modulator (neuron-specific)
		Salidroside	Inhibition of the AMPK signaling pathway; Inhibition of	Reduction of excessive mitochondrial fission	High safety profile (LD_{50} > 5 g/kg); direct repression of	50 mg/kg（ischemic rat models)	Diabetes-related vascular diseases,	(Chen et al. 2017; Zhang et al. 2021;	Class III<br>Low PAINS fast deglycosylation	Tier II: Context-dependent putative

**Regulatory Dimension**	**Core Mechanism**	**Representative Metabolite**	**Key Target/ Pathway**	**Biological Effect**	**Interaction & Synergy**	**Concentration**	**Disease Application**	**References**	**Evidence Classification**	**Mitophagy Evidence Tier**
			DRP1 and upregulation of MFN2	and promotion of mitochondrial fusion	DRP1 alongside elevation of MFN2		ischemic diseases	Zhuang et al. 2017)	, effective solely in ischemia rodents.	mitophagy regulator
	Gene regulation	Cocaine	mRNA expression of related nuclear genes, Egr3	Upregulation of PGC-1α and DRP1, balancing fission and fusion	Engagement of a distinct EGR3-centered transcriptional pathway; highlights a potential target for substance use disorder	20 mg/kg/d (rodent models)	Neuroadaptation and behavioral changes	(Cole et al. 2021)	Class I<br>Low PAINS; Toxic mitochondrial disruptor, no therapeutic human trials.	Tier III: No direct mitophagy activity evidence
		Flazin	Transcriptional remodeling to enforce a pro-fusion state; Upregulatio	Inhibition of mitochondrial fragmentation, restoration	Transcriptional remodeling to enforce a pro-fusion state; efficacy in Ca^{2+} overload	10-50 μM (oxidative stress models)	Oxidative stress and mitochondrial disorder-related kidney diseases	(Wu et al. 2023)	Class II<br>Low PAINS; merely renal cell and single mouse oxidative data.	Tier III: No direct mitophagy activity evidence

Regulatory Dimension	Core Mechanism	Representative Metabolite	Key Target/ Pathway	Biological Effect	Interaction & Synergy	Concentration	Disease Application	References	Evidence Classification	Mitophagy Evidence Tier
			n of MFN2 and inhibition of DRP1	of branched mitochondrial structure, and increases in mitochondrial length and network size	models remain unclear					
	Promote fusion	Resveratrol	AMPK/SIRT1/PGC-1α	Upregulation of PGC-1α and MFN2, promoting mitochondrial fusion and elongation	Distal transcriptional reprogramming for MFN2 upregulation	10-50 μM (podocyte models)	Diabetic nephropathy, obesity-related metabolic syndrome	(Martínez-Abundis et al. 2016; Ren et al. 2017; Meza-Torres et al. 2020)	Class II<br>High PAINS; conjugated metabolites dominate human blood, inconsistent clinical results.	Tier II: Context-dependent mitophagy-associated regulator

Regulatory Dimension	Core Mechanism	Representative Metabolite	Key Target/ Pathway	Biological Effect	Interaction & Synergy	Concentration	Disease Application	References	Evidence Classification	Mitophagy Evidence Tier
		Gastrodin (GAS)	Increase in nuclear translocation of Nrf2 and upregulation of Mfn1/2 and OPA1	Promotion of mitochondrial fusion, achieving long-range protection at the transcriptional level	Involvement of Nrf2, traditionally an antioxidant transcription factor, suggesting indirect regulation of fusion genes	100-200 mg/kg (stroke models)	Vascular dementia, ischemic stroke	(Cheng et al. 2020; YX Chen et al. 2024)	Class III<br>Low PAINS; short half-life, no human mitochondrial clinical trials.	Tier III: No direct mitophagy activity evidence
		Ginsenoside Rg1	Increase in mitochondrial length and MFN2 expression	Significant reduction in the proportion of fragmented mitochondria and improvement in energy	Gentler, more sustained stimulation; additional modulation of other pathways (e.g., PI3K/Akt)	12.5 μM (cardiomyocyte models)	Neuroprotection, improve energy metabolism	(Dong et al. 2016)	Class III<br>Low PAINS; P-gp efflux limits oral absorption, no human RCTs.	Tier III: No direct mitophagy activity evidence

Regulatory Dimension	Core Mechanism	Representative Metabolite	Key Target/ Pathway	Biological Effect	Interaction & Synergy	Concentration	Disease Application	References	Evidence Classification	Mitophagy Evidence Tier
				metabolism						
	Enhancement of mitochondrial supercomplex function	Punicalagin	Stat3-PTP1B signaling axis	Improvement of energy metabolism imbalance	Synergy with resveratrol; impact on fission-fusion machinery (DRP1/OPA1) is unreported	10 μM (cardiomyocyte models); 90 mg/kg/d (diabetic rat models)	Inflammatory bowel disease, hypoxic-ischemic brain injury	(Fu et al. 2021; Y-H Wang et al. 2023)	Class III<br>Medium PAINS; relies on gut-generated urolithin A, variable human response.	Tier III: No direct mitophagy activity evidence
	Ion homeostasis	Matrine	Inhibition of the Ca^{2+}-OMA1 axis to reduce L-OPA1 cleavage	Maintenance of the mitochondrial fusion state	Safeguarding of fusion under Ca^{2+} disequilibrium; narrow window of ion-dependent activity	6 mg/kg IV (renal injury models)	Renal ischemia-reperfusion injury	(You et al. 2020)	Class III<br>Low PAINS; oral first-pass loss, intravenous-only potent efficacy.	Tier III: No direct mitophagy activity evidence
	Targeted intervention	Baicalein	Activation of KLF4 to enhance MARCH5	Reduction of mitochondrial fission,	Tuning of the fission threshold; improvement	IC_{50} ~ 10 μM (hepatocyte models); 50	Improve liver function,	(de Oliveira et al. 2015; Hu et al.	Class III<br>Medium PAINS; liver-	Tier II: Context-dependent putative

Regulatory Dimension	Core Mechanism	Representative Metabolite	Key Target/ Pathway	Biological Effect	Interaction & Synergy	Concentration	Disease Application	References	Evidence Classification	Mitophagy Evidence Tier
	n at key nodes		expression, leading to ubiquitination and degradation of DRP1 receptors	maintenance of autophagy balance, and inhibition of apoptosis	of membrane-potential repair efficiency by ~ 1.8x; potential off-target effects on other E3 ligases	mg/kg (liver injury models)	inhibit liver fibrosis	2022; Yu et al. 2020)	selective distribution, absent clinical evidence.	mitophagy regulator
		Sanggenon C	Blockade of ERK1/2 phosphorylation to inhibit DRP1 Ser616 site activation	Inhibition of excessive mitochondrial fission and protection of cells from damage	Context-dependent efficacy (ERK inhibition may reduce proliferation independently of DRP1)	9 µM (gastric cancer cell proliferation and mitochondrial fission inhibition models)	Suppresses tumor cell proliferation	(Chen et al. 2022)	Class I<br>Extreme PAINS; only tumor cell/xenograft data, no normal tissue proof.	Tier III: No direct mitophagy activity evidence
Quality Control (Mitophagy)	Activation of the PINK1/Par	Ginsenoside 20(S)-Rh2	The PINK1/Parkin pathway	Promotion of Parkin translocation and	Efficient removal necessitates biogenesis	10-20 µM (Parkinson’s disease models)	Parkinson's disease treatment	(Gao et al. 2020)	Class III<br>Low PAINS; poor water	Tier II: Context-dependent putative

Regulatory Dimension	Core Mechanism	Representative Metabolite	Key Target/ Pathway	Biological Effect	Interaction & Synergy	Concentration	Disease Application	References	Evidence Classification	Mitophagy Evidence Tier
	kin pathway			enhancement of ubiquitination modification, facilitating clearance of α-synuclein-toxic mitochondria	replenishment; fission provides "cleavable" substrates for autophagy				solubility, only tumor animal models.	mitophagy regulator
		Artemisinin	Iron-dependent ROS generation leading to PINK1 accumulation	Triggering of mitophagy in tumor cells	High specificity for iron-rich microenvironments; high doses risk excessive stress	IC_{50} ~ 1-10 μM (leukemia models)	Leukemia, solid tumor treatment	(Zhu et al. 2022 ; Mancuso et al., 2020)	Class III<br>Low PAINS; activity iron-dependent, invalid for metabolic disorders.	Tier II: Context-dependent putative mitophagy regulator

Regulatory Dimension	Core Mechanism	Representative Metabolite	Key Target/ Pathway	Biological Effect	Interaction & Synergy	Concentration	Disease Application	References	Evidence Classification	Mitophagy Evidence Tier
	Activation of the SIRT1-PINK1-Parkin signaling cascade	Quercetin	The SIRT1-PINK1-Parkin signaling cascade	Amplification of autophagic flux	Action upstream of mTOR to avoid global immunosuppression; milder, more durable effects	5-20 μM (neuroprotection models)	Neuroprotection and anti-aging	(Vafadar et al. 2020)	Class II<br>Extreme PAINS; low oral uptake, weak blood-brain tissue penetration.	Tier I: Well-validated selective mitophagy modulator (neuron-specific)
	Regulation of the BNIP3/NIX pathway	Luteolin;	Inhibition of BNIP3L/NIX	Blockade of excessive autophagy and protection of neurons	Exemplification of state-dependent fine-tuning; receptor-level specificity	10-20 μM (glutamate-induced neuroinflammation models)	Glutamate-induced neuroinflammation model	(Ma et al. 2024; Vongthip et al. 2024)	Class II<br>High PAINS; rich cell data without human neurodegeneration trials.	Tier II: Putative mitophagy inhibitor (unvalidated selectivity)
	PINK1/Parkin-dependent pathway or HIF-1α/BNIP3 axis	Thiamine	Maintenance of normal HIF-1α/BNIP3 axis function	Upregulation of BNIP3 expression, initiating BNIP3-mediated	Evidence primarily from deficiency models; direct molecular	0.81-1.32 mg/kg (dietary supplementation for grass carp under hypoxia)	Alleviate mitochondrial damage under hypoxia	(Huang et al. 2024)	Class IV<br>Low PAINS; Multiple mitochondrial disorder human RCTs,	Tier III: Mitochondrial homeostasis regulator (no direct mitophagy

Regulatory Dimension	Core Mechanism	Representative Metabolite	Key Target/ Pathway	Biological Effect	Interaction & Synergy	Concentration	Disease Application	References	Evidence Classification	Mitophagy Evidence Tier
				mitophagy under hypoxic conditions	targets remain unverified				consistent PK/PD.	flux evidence)
	Activation of the AMPK-mTOR-ULK1 pathway	Berberine	AMPK-mTOR-ULK1 pathway	Inhibition of mTORC1 and activation of ULK1, leading to clearance of damaged mitochondria	Robust AMPK activation; dose-dependent toxicity (cardiotoxicity via hERG); potential exacerbation of energetic compromise in vulnerable tissues	25-35 μM (concentration-dependent mitophagy induction)	Type 2 diabetes, cancer	(Xie et al. 2023; Zhao et al. 2019; Feng et al., 2021)	Class III<br>Low PAINS; hERG cardiotoxic risk, scarce neurological human trials.	Tier I: Well-validated selective mitophagy modulator
	mTOR-Beclin-1 pathway	Dihydrochalcone (DDC)	Inhibition of mTOR-Ser2448 phosphorylation and	Activation of autophagy to clear damaged	Dual-pathway agent: induces autophagy and mitochondrial apoptosis	52.5-66.4 μM (breast cancer cell mitophagy and intrinsic	Potential anticancer agent	(Liu et al. 2023; Mendez-Callejas et al. 2024;	Class II<br>Medium PAINS; only breast tumor	Tier III: No validated mitophagic activity

Regulatory Dimension	Core Mechanism	Representative Metabolite	Key Target/ Pathway	Biological Effect	Interaction & Synergy	Concentration	Disease Application	References	Evidence Classification	Mitophagy Evidence Tier
			upregulation of Beclin-1	organelles; disruption of mitochondrial membrane potential to activate the mitochondrial apoptotic pathway	while rebalancing morphology	apoptosis models)		Xiao et al. 2017)	data, universal protection unproven.	
	Gut microbiota-mitochondria axis	Urolithin A (microbiota metabolite)	Parkin-PINK1 pathway	Activation of autophagy and enhancement of muscle mitochondrial function	Circumvention of oral bioavailability bottlenecks; efficacy depends on individual "metabotype" (e.g.,	500 and 1000 mg (clinical doses for muscle health)	Senile muscle decline (clinically verified)	(Singh et al. 2022; X Li et al. 2023; Kern et al. 2025; Ryu et al. 2016; Y-H Wang et al. 2023)	Class IV<br>Low PAINS; human muscle RCTs confirm stable mitochondrial regulation.	Tier I: Well-validated selective mitophagy inducer

Regulatory Dimension	Core Mechanism	Representative Metabolite	Key Target/ Pathway	Biological Effect	Interaction & Synergy	Concentration	Disease Application	References	Evidence Classification	Mitophagy Evidence Tier
					Gordonibacter abundance)					
Defense Mechanism (Oxidative Stress)	Direct scavenging of ROS	Ellagic Acid	·OH (Hydroxyl radical)	Stabilization of membrane potential and reversal of mitochondrial swelling	ROS reduction decreases mitochondrial damage, easing quality control load; lower ROS also lessens fission stress, favoring morphological stability	1.5-3.0 μg/mL (cell models)	Metabolic syndrome	(Gong et al. 2022; Liu et al. 2021；Teel et al., 1986b)	Class II<br>High PAINS; parent compound undetectable in human systemic circulation.	Tier III: No direct mitophagy activity evidence
	Enzyme induction	EGCG	Phase II detoxifying enzymes (e.g., GST, GPX, HO-1)	Establishment of a rapid antioxidant defense system	Rapid action; exhibits hormesis (pro-oxidant at high doses > 50 μM)	5-50 μM (cancer/neuroprotection models)	Cancer prevention, acute neural injury treatment	(Meza-Torres et al. 2020; Xing et al. 2024; Andreu Fernández et al., 2020)	Class II<br>Extreme PAINS; plasma nanomolar levels far below in vitro effective dose.	Tier II: Context-dependent putative mitophagy regulator

Regulatory Dimension	Core Mechanism	Representative Metabolite	Key Target/ Pathway	Biological Effect	Interaction & Synergy	Concentration	Disease Application	References	Evidence Classification	Mitophagy Evidence Tier
	Synergistic regulation of ROS balance	Apigenin	Bax/Bcl-2 pathway	Scavenging of free radicals; upregulation of the Bcl-2/Bax ratio; inhibition of mitochondrial pathway apoptosis	Efficient BBB penetration and prolonged brain retention despite poor oral bioavailability	50 mg/kg IP (neuroprotection models)	Synergistic antioxidant-antiapoptosis	(Y Chen et al. 2024; Yang et al. 2021 ; Socała et al., 2025)	Class III<br>High PAINS; good brain uptake only via non-oral delivery.	Tier III: No direct mitophagy activity evidence
		Ferulic Acid	Activation of the Nrf2-NF-κB signaling axis	Synergistic regulation of ROS balance by inhibiting inflammatory responses	Concentration-dependent anti-inflammatory effects without cytotoxicity at therapeutic levels	10-100 μM (macrophage models)	Chronic inflammatory diseases	(Gong et al. 2022; Liu et al. 2021)	Class III<br>Medium PAINS; rapid hepatic conjugation, lacking large human trials.	Tier III: No direct mitophagy activity evidence

**Regulatory Dimension**	**Core Mechanism**	**Representative Metabolite**	**Key Target/ Pathway**	**Biological Effect**	**Interaction & Synergy**	**Concentration**	**Disease Application**	**References**	**Evidence Classification**	**Mitophagy Evidence Tier**
	Improvement of mitochondrial OXPHOS function	Astaxanthin	Upregulation of SOD1/2 and repair of Complex I/III	A 23-37% increase in ATP synthesis	Lipophilicity enables membrane enrichment, conferring dual benefits in enzyme induction and structural repair	80 μg/L (neuronal cell injury model)	Alleviates energy metabolism crisis in ischemia-reperfusion models	(Mularczyk et al. 2022)	Class III<br>Low PAINS; lipid-dependent absorption, no human hard endpoints.	Tier III: No direct mitophagy activity evidence
		Vitexin	Inhibition of NADPH oxidase 4 (NOX4) expression via the Epac1-Rap1 signaling pathway; stabilization of Complex III electron	Reduction of ROS generation from the source; maintenance of oxidative phosphorylation function, leading to a	Glycosylation (C-8 glucose) enhances aqueous solubility and bioactivity; deglycosylated form loses efficacy	10 μM (myocardial ischemia/reperfusion and hypoxia/reoxygenation models)	Myocardial ischemia-reperfusion injury	(Duan et al. 2024)	Class III<br>Medium PAINS; glycoside vital for activity, low intestinal permeability.	Tier III: No direct mitophagy activity evidence

Regulatory Dimension	Core Mechanism	Representative Metabolite	Key Target/ Pathway	Biological Effect	Interaction & Synergy	Concentration	Disease Application	References	Evidence Classification	Mitophagy Evidence Tier
			transfer efficiency	significant increase in ATP synthesis rate						
	Enzyme induction and ETC optimization	Caffeic Acid Phenethyl Ester (CAPE)	Enhancement of Complex I function and increase of SOD/CAT activity	Reduction of ROS leakage	Enhancement of ETC efficiency to minimize ROS at the source; favorable organ tolerance	10-30 μmol/kg (cardiotoxicity models)	Placental protection in preeclampsia; mitochondrial function reconstruction in fatty liver	(Akyol et al. 2014; Ali et al. 2020; Zhang et al. 2014; Zhao et al. 2025)	Class III<br>Medium PAINS; poor oral absorption, most data from injection models.	Tier III: No direct mitophagy activity evidence
	Transcriptional reprogramming	Sulforaphane (SFN)	Activation of the Nrf2/ARE pathway, inducing HO-1/NQO1 expression; PGC-1α-	Enhancement of maximum respiration, spare respiratory capacity, basal respiration	Canonical NRF2 activator; reliable efficacy and favorable aqueous solubility	5-50 μM (placental models)	Maintain fission/fusion threshold	(Langston-Cox et al. 2020)	Class III<br>Low PAINS; unstable orally, lacking human mitochondrial RCTs.	Tier II: Context-dependent putative mitophagy regulator

**Regulatory Dimension**	**Core Mechanism**	**Representative Metabolite**	**Key Target/ Pathway**	**Biological Effect**	**Interaction & Synergy**	**Concentration**	**Disease Application**	**References**	**Evidence Classification**	**Mitophagy Evidence Tier**
			dependent respiratory chain optimization	, and ATP production ; decrease in proton leak; repair of hypoxic damage						
	Regulation of dynamics balance	Baicalein	KLF4-MARCH5-DRP1 axis	Reduction of fragmentation and a 1.8-fold increase in membrane potential repair efficiency	Mitigation of fragmentation indirectly fortifies antioxidant capacity; establishment of mtROS-ATP feedback with baicalin	50 mg/kg (liver injury models)	Neuroprotection	(de Oliveira et al. 2015; X Li et al. 2023; Wu et al. 2021 ; Yu et al., 2020)	Class III<br>Medium PAINS; liver-selective distribution, absent clinical evidence.	Tier III: No direct mitophagy activity evidence
		Luteolin	Activation of SOD2 and enhancement of OPA1-mediated	Reduction of superoxide levels and increase in	Stabilization of redox resilience by buttressing structural stability;	10-20 μM (cell models)	Precise regulation of redox homeostasis	(Ma et al. 2024)	Class II<br>High PAINS; rich cell data without human	Tier II: Context-dependent putative

Regulatory Dimension	Core Mechanism	Representative Metabolite	Key Target/ Pathway	Biological Effect	Interaction & Synergy	Concentration	Disease Application	References	Evidence Classification	Mitophagy Evidence Tier
			cristae remodeling	cristae density	hemolysis risk at concentrations > 20 μM		in metabolic diseases		neurodegeneration trials.	mitophagy regulator
	Hormetic tuning of ROS	Arecoline	SIRT3-SOD2/IDH2 axis	Control of ROS within a physiological fluctuation range to prevent excessive oxidative damage	Low-dose ROS triggers protective autophagy; high-dose ROS overwhelms buffering capacity; SIRT3-dependent	no specific concentration parameters	Improve oxidative stress in metabolic syndrome	(S Wang et al. 2023)	Class II<br>Low PAINS; Dose-dependent mitochondrial toxicity, lacking human safety data.	Tier III: No direct mitophagy activity evidence

This table integrates molecular mechanisms through which natural products cooperatively regulate mitochondrial function via the Four-Dimensional Framework (Biogenesis, Morphodynamics, Quality Control, Defense). In Biogenesis, AMPK/SIRT1-PGC-1α activation (e.g., EGCG-enhanced mtDNA replication) fuels mitochondrial biogenesis. Morphodynamics rebalances DRP1/OPA1-MFN1/2 (e.g., matrine-maintained fusion competence via OMA1 inhibition). Quality Control couples mitophagy with dynamics (e.g., berberine-mediated clearance of damaged mitochondria through AMPK-mTOR-ULK1). Defense mechanisms establish oxidative stress feedback (e.g., astaxanthin-induced 23-37% OXPHOS efficiency gain). Multi-target synergy is elaborated in Section 4.5 (AMPK/SIRT1 hub integrating energy sensing and autophagy initiation), while metabolic conversion bottlenecks are addressed in Section 5.1.2 (gut microbiota-dependent urolithin A generation).
