# Supplementary material for: Natural products targeting mitochondrial homeostasis: a four-dimensional framework integrating dynamics, mitophagy, pharmacokinetics, and translational safety
Source: Front Pharmacol. 2026 Sep 18;17:1911495. doi: 10.3389/fphar.2026.1911495 (PMC13670514; doi:10.3389/fphar.2026.1911495)
Supplement: Supplementary file 2 [file Table2.pdf]

Supplementary Table 2

Overview of Pharmacokinetic Characteristics and Safety Risks of Natural Products Regulating Mitochondrial Dynamics

Category	Representative Metabolite	Pharmacokinetic Characteristics	Pharmacokinetic parameters	Toxicity Manifestations	Toxicological parameters	References
First-pass effect dominant type	Curcumin	Oral bioavailability dominated by first-pass effect; short half-life; rapid metabolism via β-diketone motif cleavage; species-specific metabolism; low C_{max} relative to *in vitro* efficacious concentrations<br>Main metabolite: 3'-OH glucuronide (humans), 4'-OH glucuronide (rats)	Oral bioavailability: ≤ 1% (rats), 0.001-0.04% (humans)<br>$T_{1/2}$: 11.96 ± 2.64 min (rat)<br>C_{max}: 1.7 μM (humans, after 8 g oral dose)<br>*In vitro* efficacious concentrations: 5-50 μM	Dose-related hepatic perturbations; synergistic pro-oxidant and immune-modulating effects with pretilachlor co-exposure; gastrointestinal symptoms in clinical use	ALP elevation: Rats (50 mg/kg)<br>Clinical dose for symptoms: 0.9-3.6 g/day (nausea, diarrhea, ALP/LDH elevations)	(Jelezova et al. 2015; Wang et al. 2016; Liu et al. 2022; Otuechere et al. 2014)
	Resveratrol	Oral bioavailability affected by extensive first-pass conjugation; high absorption rate but rapid metabolism; circulating conjugate concentrations higher than aglycone; IV administration bypasses first-pass effect; blood-brain barrier penetration;	Oral bioavailability: ~ 6%<br>Absorption rate: ~ 75% (from 500 mg dose)<br>IV dose for systemic exposure: 5 mg/kg<br>$T_{1/2}$: ~ 7-9 h (rats, longer than humans)<br>Vd: Larger in rats than humans	Generally well tolerated at moderate doses; mild-moderate gastrointestinal symptoms at higher doses	Dose for gastrointestinal symptoms: 2.5-5 g	(Cerqueira et al. 2025; Kapetanovic et al. 2010; Martínez-Abundis et al. 2016; Sergides et al. 2016; Ren et al. 2017)

Category	Representative Metabolite	Pharmacokinetic Characteristics	Pharmacokinetic parameters	Toxicity Manifestations	Toxicological parameters	References
		species differences in volume of distribution (Vd) and half-life				
	Quercetin	Low oral bioavailability; short distribution and elimination half-lives; specific clearance (CL) and volume of distribution (Vd); high concentration in liver, kidney, intestine; weak brain penetration; no accumulation; metabolism to multiple derivatives; excretion via urine, bile, feces	Oral bioavailability: 1-5%<br>T_{max}: 0.7 ± 0.3 h<br>Distribution $T_{1/2}$: 0.7-7.8 min<br>Elimination $T_{1/2}$: 3.8-86 min<br>CL: 0.84 L/min/m²<br>Vd: 3.7 L/m²	Safety at low oral doses; toxicity (vomiting, hypertension, nephrotoxicity, hypokalemia) with high-dose IV administration	Safe oral dose: ≤ 1 g/day (humans)<br>IV dose for toxicity: > 945 mg/m²	(Rayamajhi et al. 2013; Vafadar et al. 2020)
	Isochlorogenic Acid A (ICGA)	Absolute bioavailability with calculated and observed values; highest absorption in ileum via passive diffusion (unique absorption window); metabolism to multiple products (N1-N9); excretion mainly via feces, followed by bile	Absolute bioavailability: Calculated 30.71%, observed 22.6% (rats)<br>Fecal excretion (prototype): 40.11%<br>Biliary excretion: 6.88%	Low acute toxicity in Wistar rats and birds; good long-term tolerability of chlorogenic acid (CQA); *in vitro* DNA double-strand breaks; non-mutagenicity in bacterial assays; potential allergenicity of CQA	No quantitative toxicity data specified	(Behne et al. 2023; Tian et al. 2017; Wang et al. 2021)

Category	Representative Metabolite	Pharmacokinetic Characteristics	Pharmacokinetic parameters	Toxicity Manifestations	Toxicological parameters	References
	Matrine	Oral and intramuscular bioavailability differences; biphasic pharmacokinetic model in humans (IV); high distribution in liver, spleen, kidney; minimal distribution in brain/adipose; oxidative metabolism to matrine N-oxide; urinary excretion as main route	Oral bioavailability: 19.4% (dog), 31.6-47% (rat)<br>Intramuscular bioavailability: 104.43%<br>IV dose (humans): 6 mg/kg<br>$T_{1/2}\alpha$: 19.21 min (humans, IV)<br>$T_{1/2}\beta$: 184.22 min (humans, IV)<br>Urinary excretion: 52.75% (within 32 h)<br>Renal clearance: 143.79 mL/min	Dose-dependent hepatotoxicity; zebrafish toxicity; nausea, dizziness, injection-site pain in clinical IV use; acute poisoning risk with high crude drug dose	Hepatocyte IC_{50}: 5.3 mmol/L<br>Zebrafish LC_{50}: 0.41 mmol/L<br>Crude drug dose for acute poisoning risk: > 10 g	(You et al. 2020)
Transporter and microbial metabolism dependent type	EGCG	Low oral bioavailability; rapid absorption, extensive tissue distribution, brisk metabolism, renal excretion; stability in gastric acid (pH < 3), degradation in small intestine alkalinity; gut-microbial conversion to valerolactones; hepatic phase II metabolism; intake conditions (fasting/food)	Oral bioavailability: 0.1-0.3%<br>Gastric acid stability: pH < 3<br>Intake effect: Fasting increases bioavailability/absorption; food reduces exposure	Dose-dependent risk of hepatotoxicity, dizziness, anemia; higher risk in individuals with NQO1 C609T polymorphism; narrow therapeutic window (especially fasting)	Dose for toxicity: ≥ 800 mg/day (hepatotoxicity, dizziness, anemia)<br>Polymorphism associated with risk: NQO1 C609T	(Andreu Fernández et al. 2020; Capasso et al. 2025; Pervin et al. 2019; Hazimeh et al. 2023; Kim and Kim 2013)

Category	Representative Metabolite	Pharmacokinetic Characteristics	Pharmacokinetic parameters	Toxicity Manifestations	Toxicological parameters	References
		influencing exposure; inter-individual variability				
	Punicalagin	Extremely low direct plasma exposure (poor bioavailability/rapid clearance); hydrolysis to ellagic acid (EA) (absorbed and extensively conjugated); gut microbiota (e.g., Gordonibacter)-mediated conversion of EA to urolithins (UA predominant); conjugated UA peak time; high inter-individual variability dependent on microbiota composition ("metabotypes")	Conjugated UA peak time: ~ 24 h<br>No quantitative bioavailability data specified	Metabolite-induced perturbation of hepatic iron handling and renal function at high doses; amelioration of tumor-induced intestinal barrier dysfunction	No quantitative toxicity data specified	(Hu et al. 2020; Y-H Wang et al. 2023)
	Baicalin	Hydrolysis to active aglycone baicalein by intestinal β-glucuronidase; route-dependent absorption and bioavailability (oral: slower absorption, lower bioavailability; IV: rapid distribution, faster elimination);	No quantitative bioavailability/half-life data specified	Selective cytotoxicity toward cancer cells; low toxicity to normal cells; favorable animal tolerability; hepatoprotective effects in some models; potential	No quantitative toxicity data specified	(Hu et al. 2022)

Category	Representative Metabolite	Pharmacokinetic Characteristics	Pharmacokinetic parameters	Toxicity Manifestations	Toxicological parameters	References
		relatively long half-life supporting sustained exposure		dependence risk with long-term use		
Blood-brain barrier penetrating type	Apigenin	Poor oral bioavailability; extensive first-pass metabolism; enterohepatic recirculation; efficient brain entry and prolonged retention; concurrent serum/brain T_{max} in mice (IP); longer brain elimination half-life than serum; brain accumulation ratio (AR); metabolism via CYP1A1/2B/2E1 to hydroxylated products then glucuronidation; extrahepatic metabolism suggested by apparent CL/F	Oral bioavailability: Poor<br>IP dose (mice): 50 mg/kg<br>T_{max}: 0.25 h (concurrent serum/brain, mice, IP)<br>Elimination $T_{1/2}$: 48.2 h (brain), 12.86 h (serum, mice, IP)<br>AR: 3.8<br>Apparent CL/F: 19.33 L/h/kg (mice)	Low acute toxicity; non-fatal at high oral doses in preparations; transient platelet inhibition at high acute doses; preserved hematologic, hepatic, renal indices; no motor deficits after 14-day IP dosing (suggesting neuroprotection)	IP: 25-150 mg/kg (low toxicity)<br>Oral dose in preparations: Up to 2000 mg/kg (non-fatal)<br>IP dosing duration for no motor deficits: 14 days	(Socała et al. 2025; Alelign et al. 2020)
	Echinacoside	Negligible parent exposure orally; low absolute oral bioavailability; T_{max} post oral dose in rats; gut-microbial conversion to aglycone then verbascoside; rapid phase II conjugation limiting circulating	Absolute oral bioavailability: ~ 0.83%<br>Oral dose (rats): 100 mg/kg<br>T_{max}: 15 min (rats, oral)	High safety in acute/long-term studies; no mutagenicity, carcinogenicity, or reproductive toxicity	No quantitative toxicity data specified	(Liu et al. 2018)

Category	Representative Metabolite	Pharmacokinetic Characteristics	Pharmacokinetic parameters	Toxicity Manifestations	Toxicological parameters	References
		parent to nM range; BBB penetration under cerebral ischemia (limited under physiology); prolonged half-life in Parkinson's Disease models (reduced CL); main renal excretion	$T_{1/2}$: 74.4 min (normal rats), 121.6 min (Parkinson's Disease models)<br>Circulating parent concentration: nM range			
Protein binding dominant type	Sanggenon C	Poor aqueous solubility (pH-dependent, low at pH 7.4); broad pKa range; moderate lipophilicity (logP); solubility decline in multicomponent extracts; low systemic exposure, rapid clearance, no accumulation in mice; minimal lung levels, no splenic signal; liver recovery suggesting tissue binding/accelerated biotransformation; uptake via passive diffusion, poor transepithelial passage in Calu-3 cells	Aqueous solubility: Poor (low at pH 7.4)<br>pKa range: 2.64-12.05<br>logP: 3.48<br>Serum concentration (mice): 8-124 ng/mL (1h), < 2.77 ng/mL (24 h)<br>Liver recovery: 26%<br>Calu-3 cell IC_{50}: 47.8 μg/mL	Maximum tolerated dose (MTD) defined in mice (weight loss threshold); higher *in vitro* cytotoxicity than stereoisomer sanggenon D (due to higher lipophilicity)	MTD (mice): > 10% weight loss at 300 mg/kg oral MA60 extract	(Langeder et al. 2023; Schwarzinger et al. 2024)

Category	Representative Metabolite	Pharmacokinetic Characteristics	Pharmacokinetic parameters	Toxicity Manifestations	Toxicological parameters	References
	Rosmarinic Acid	Low oral absorption fraction despite favorable in silico drug-likeness; low intestinal permeability (mainly paracellular transport); extensive binding to plasma proteins (e.g., HSA); gut microbiota-mediated degradation to simpler phenolic acids; hepatic phase II metabolism; rapid plasma appearance and T_{max}; food effects on C_{max} and T_{max}; predominant renal excretion (rapid clearance); specific pharmacokinetic parameters in rats	Oral absorption fraction: ~ 1%<br>Renal clearance: Cleared within 6h, traces at 24-48 h<br>$T_{1/2}$: 0.75 h (rats)<br>MRT: 0.32 h (rats)<br>CL: 1.02 L/h/kg (rats)	Good tolerability in clinical trials and animal studies at reasonable exposures; potential hepatic metabolism strain in susceptible individuals at high doses or prolonged use	No quantitative toxicity data specified	(Nunes et al. 2015; Yehya et al. 2019Nunes et al. 2015; Hitl et al. 2020)
	Ginsenoside Rg1	Very low oral bioavailability (affected by intestinal first-pass metabolism, P-gp efflux, limited solubility); rapid absorption; specific T_{max} and relative bioavailability in rats; wide distribution (liver, kidney,	Oral bioavailability: 1.9-20%<br>Absorption: Detection in plasma at 15 min<br>Oral dose (rats): 300 mg/kg<br>T_{max}: 0.5 h (rats)	Enhancement of reproductive parameters in rodents; ovarian function and embryonic development impairment at high doses; mild, reversible events at customary	No quantitative toxicity dose data specified	(Shi et al. 2013; Inam et al. 2017; Ni et al. 2015; Ma et al. 2011; Gao et al. 2017)

Category	Representative Metabolite	Pharmacokinetic Characteristics	Pharmacokinetic parameters	Toxicity Manifestations	Toxicological parameters	References
		heart); biphasic kinetics; metabolism to active derivatives; minor CYP450 hydroxylation; mass balance with feces as main excretion route	Relative bioavailability (rats): ~ 2.5% (300 mg/kg oral)<br>Active derivative half-life (PPT): ~ 24 h<br>Mass balance: ~ 72% recovered in feces (40.11% parent), 6.88% biliary, 0.04% urinary<br>CYP450 hydroxylation: < 5% parent levels	exposures; hypertension/hypoglycemia risk with prolonged/high-dose use		
	Berberine	Low oral bioavailability (preclinical) is imposed by poor cationic transcellular permeability, P-gp efflux, gut-microbial reduction and extensive hepatic first-pass metabolism; absorption is dose-dependent. Distribution is broad (tissue conc. > plasma) with a rat-specific Vz/F. The metabolite undergoes sequential Phase I (CYP2D6/3A4/1A2/2E1/2C19)	Oral bioavailability: 0.37 ± 0.11% (preclinical)<br>Vz/F: 12-38 L/kg (rats)<br>Fecal excretion (M1): 18.6%<br>Total excretion recovery: 41.2%	Dose-dependent multisystem toxicity; cardiotoxicity (hERG K^+ channel blockade, QT prolongation, torsades de pointes risk); GI irritation, neuromuscular signs, gastric injury; subacute toxicity (reduced spleen weight, CD^{4+}/CD^{8+} counts, ALT/AST↑); chronic toxicity (UVA phototoxicity, pro-	hERG K^+ channel blockade IC_{50}: 3.1-80 μM (IV)<br>Subacute dose for spleen/immune effects: 10 mg/kg (IP)<br>Subacute dose for ALT/AST↑:	(Feng et al. 2021; Rad et al. 2017)

Category	Representative Metabolite	Pharmacokinetic Characteristics	Pharmacokinetic parameters	Toxicity Manifestations	Toxicological parameters	References
		→ berberrubine (M1), demethyleneberberine (M2), jatrorrhizine (M3) followed by predominant Phase II metabolism (glucuronidation M4, M6, M7, M9; sulfation M5, M8) yielding higher exposure than Phase I products. Excretion is via urine (glucuronidates), feces (M1) and bile (low).		atherogenic effects); reproductive toxicity; neonatal hyperbilirubinemia risk (G6PD deficiency); normal cell toxicity (ROS, mitochondrial apoptosis, DNA damage) at high conc	≥ 150 mg/kg (oral)<br>Reproductive toxicity LOAEL: 531 mg/kg (rat)<br>Normal cell toxicity concentration: > 100 μM	
Tissue accumulation type	Salidroside	Favorable absorption in rats; rapid absorption-distribution in dogs (short T_{max}, appreciable C_{max}, brief $T_{1/2}$); higher concentration in skeletal muscle than plasma; lower concentration in brain, liver, adipose; metabolism via enzymatic hydrolysis to tyrosol; urinary excretion as main route (higher than fecal/biliary)	No quantitative absorption/bioavailability/half-life data specified	High LD_{50}; well tolerated in animals (no significant toxicities with prolonged use); no genotoxicity in standard assays	LD_{50}: > 5.63 ± 0.33 g/kg	(Zhang et al. 2021; Chen et al. 2017)

Category	Representative Metabolite	Pharmacokinetic Characteristics	Pharmacokinetic parameters	Toxicity Manifestations	Toxicological parameters	References
	Ferulic Acid	Rapid absorption; extensive tissue distribution; low oral bioavailability; brisk metabolism; fast excretion; transdermal delivery-induced marked exposure improvement compared with oral delivery	Absorption: T_{max}; 9min (rats)<br>Plasma $AUC_{0\text{-}t}$ (transdermal vs oral): 953.90 vs 281.47 min·mg/L<br>Relative bioavailability: 57.9% (plasma), 463.4% (skin microdialysates)	Concentration-dependent anti-inflammatory effects; absence of cytotoxicity at therapeutic levels	Anti-inflammatory concentration range: 10-100 μM (in macrophages)	(Gong et al. 2022; Yan et al. 2020)
	Gastrodin	Rapid oral absorption; wide distribution to liver, kidney, and heart; conversion to active metabolite HBA; short half-life; low plasma-protein binding; hepatic metabolism generating multiple products; dominant renal excretion; minor biliary elimination	Oral absorption: T_{max}: 1-4 h	Favorable safety margin; no mortality or major adverse reactions in acute studies; no perturbed growth, development, or organ function in chronic administration; absence of overt toxicity in primary metabolites	No quantitative toxicity dose data specified	(H Gao et al. 2025; R Li et al. 2021)
	Chicoric Acid	Slow oral absorption; slow elimination; specific pharmacokinetic parameters (V/F, CL/F, MRT); higher tissue levels in liver, lung, and kidney than in brain (modest	Oral absorption slow, $T_{1/2}$: 4.53 ± 1.44 h (rat)<br>V/F = 17.94 ± 5.28 L/kg<br>CL/F = 2.80 ± 0.46 $L\cdot kg^{-1}\cdot h^{-1}$<br>MRT = 18.58 ± 4.43 h	Limited toxicological data; good tolerance at customary doses in available *in vitro*/animal studies	No quantitative toxicity dose data specified	(Wang et al. 2016)

Category	Representative Metabolite	Pharmacokinetic Characteristics	Pharmacokinetic parameters	Toxicity Manifestations	Toxicological parameters	References
		CNS penetration); high plasma protein binding	Plasma protein binding: 98.3% (rat), 96.9% (human), 96.6% (BSA)			
	Luteolin	Rapid absorption; slow elimination; ~ linear kinetics; low oral bioavailability; higher systemic exposure via IV administration; large Vd supporting broad tissue partitioning (liver, kidney, lung); Phase II metabolism (glucuronidation/sulfation) followed by biliary/urinary excretion	No quantitative absorption/bioavailability/half-life data specified	High acute safety; negligible hemolysis risk at therapeutic concentrations; no significant alteration of growth, organ indices, or histopathology in mice under chronic exposure	No quantitative toxicity dose data specified	(Vongthip et al. 2024)
	Neohesperidin Dihydrochalcone (NHDC)	Rapid oral absorption in rats; modest bioavailability; fit to one-compartment model; rapid distribution to heart, liver, and kidney via IV administration; route-dependent half-life; low aqueous solubility limiting absorption	Oral absorption (rats): Rapid; T_{max}: 0.167 h<br>Bioavailability: Modest (21.75%)<br>IV distribution: Rapid to heart, liver, kidney (peak at 0.083 h)<br>$T_{1/2}$: 4.7 h (oral), 0.69 h (IV)	Absence of carcinogenicity, teratogenicity, and genotoxicity (including 15-year USDA program); specified EU ADI; low cytotoxicity of most derivatives at certain concentrations; higher safety of halogen/hydroxyl substituents compared with	EU ADI = 5 mg/kg<br>Cytotoxicity of derivatives:<br>Low at ≤ 400 μM (most derivatives)	(Pérez Gutierrez et al. 2019; Liu et al. 2023; Xiao et al. 2017)

Category	Representative Metabolite	Pharmacokinetic Characteristics	Pharmacokinetic parameters	Toxicity Manifestations	Toxicological parameters	References
				methyl/methoxy substituents		
Active metabolite generating type	Artemisinin (ARS) & Derivatives (ART, DHA, ARM)	Poor aqueous solubility; bioavailability improvement by derivatives (water-soluble artesunate (ART), oil-soluble artemether/arteether); iron-dependent activation (Fe^{2+} cleaves endoperoxide bridge); targeting ability to transferrin receptor-overexpressing cells; non-P-gp substrate property; P-gp function inhibitory ability (reverses MDR)	No quantitative absorption/bioavailability/half-life data specified	Wide therapeutic window; low toxicity to normal cells; embryotoxicity during organogenesis in pregnant mice; iron-dependent ROS generation counterbalanced by antioxidant enzyme induction; high-dose DHA-induced ROS elevation (mitigated by scavengers/chelators); high-dose-induced reticulocytopenia	No quantitative toxicity dose data specified	(Mancuso et al. 2020)
	Paeonol	Rapid absorption; extensive distribution after oral dosing (highest in liver); swift systemic clearance; substantial tissue level decline by 3h; metabolism via multiple pathways (demethylation, glucuronidation, hydroxylation, gut-microbial hydrolysis)	Oral distribution: Extensive; liver peak = 3460 ng/g at 10 min (heart, spleen, kidney, lung, brain also distributed)<br>Systemic clearance: Swift; $T_{1/2}$: 0.68 h<br>Tissue level decline: Substantial by 3 h	Potential hepatotoxicity and nephrotoxicity of metabolites at high doses; high-dose-induced interference with iron metabolism; amelioration of tumor-induced intestinal barrier dysfunction	No quantitative toxicity dose data specified	(Hu et al. 2020; Chen et al. 2018; Y-H Wang et al. 2023)
